# Supplementary material for: Generation of human appetite-regulating neurons and tanycytes from pluripotent stem cells
Source: Cell Stem Cell. 2026 Jul 2;33(7):1174–1190.e11. doi: 10.1016/j.stem.2026.05.005 (PMC13353052; doi:10.1016/j.stem.2026.05.005)
Supplement: Document S1. Figures S1–S7 and Tables S1–S6 [file mmc1.pdf]

Cell Stem Cell, Volume *33*

Supplemental Information

Generation of human appetite-regulating neurons and tanycytes from pluripotent stem cells

Zehra Abay-Nørgaard, Anika K. Mueller, Erno Hänninen, Dylan Rausch, Louise Piilgaard, Lucía Sena Trujillo, Lorenzo Fedrizzi, Jens Bager Christensen, Alison Salvador, Alrik L. Schörling, Noah Wulff Mottelson, Qiuyu Qin, Shruthi Sampath, Bob Hersbach, Jonas Henkenjohann, Sofie Peeters, Viktoriia Nikulina, Charlotte Høy Kruse, Yuan Li, Kavitha Chinnaiya, Marysia Placzek, Janko Kajtez, Tune H. Pers, and Agnete Kirkeby

Supplementary figures

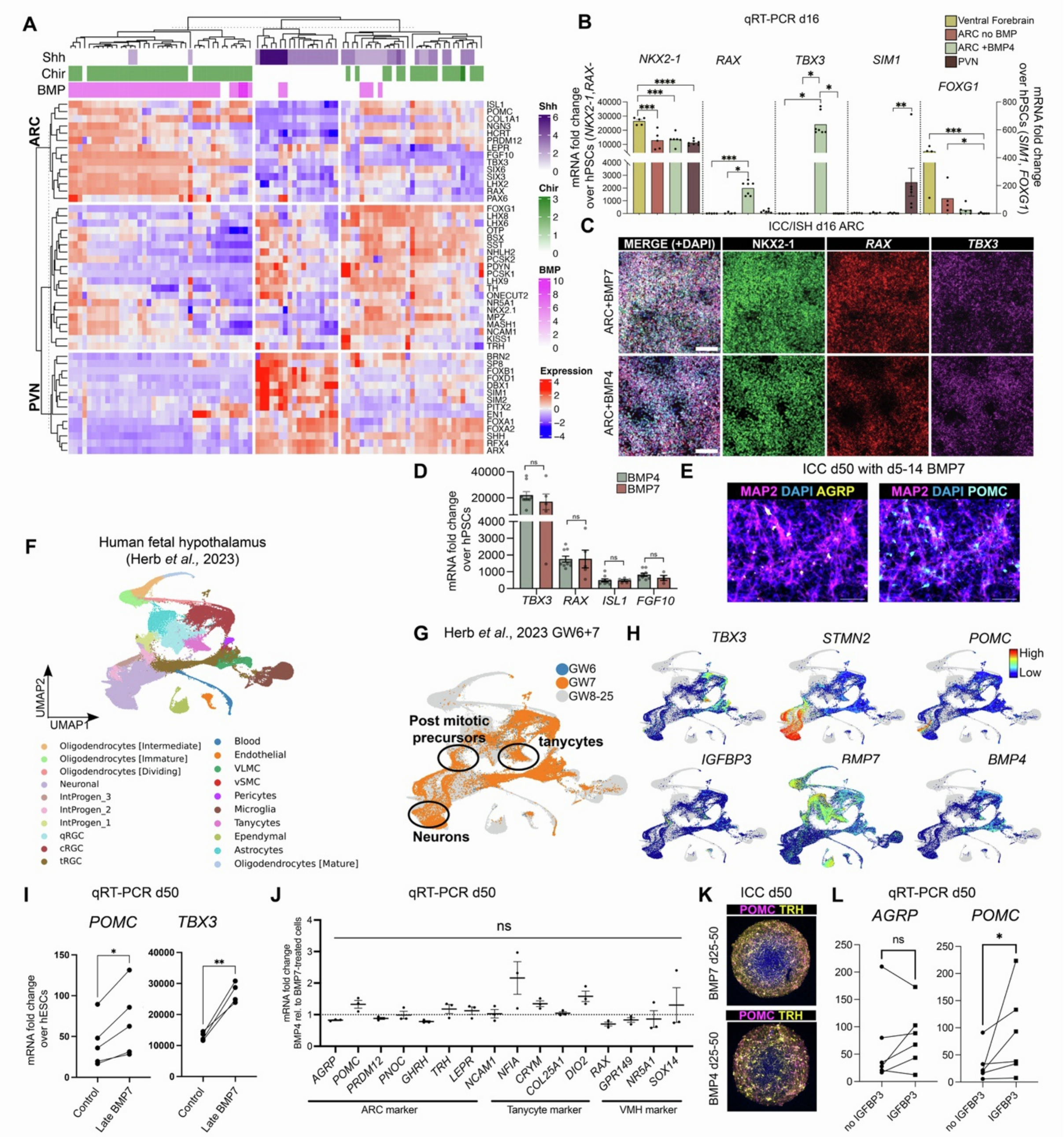

Supplementary Figure 1. BMP4 modulates ARC and PVN differentiation profiles *in vitro*, related to Figure 1.
(A) Unsupervised clustering of heatmap of d16 qRT-PCR of conditions with different morphogen timings of SHH, CHIR (Wnt agonist) and BMP (related to Figure 1B).
(B) qRT-PCR of d16 differentiations comparing PVN and ARC conditions with or without BMP4, and a ventral forebrain (VFB) differentiation as a reference, depicted as mean.
(C) ICC/ISH d16 of ARC cultures under BMP4 versus BMP7 treatment. Scale bars: 100 µm.
(D) qRT-PCR of d16 ARC treated with BMP4 or BMP7, mean ± SEM.
(E) ICC in d50 ARC cultures patterned with BMP7.
(F) UMAP of human fetal hypothalamic reference with annotated clusters[1].
(G) UMAP of scRNA-seq from gestational weeks 6–25 showing the contribution from early (GW6–7) samples[1].
(H) Feature plots of key hypothalamic markers from fetal human reference data[1].
(I) qRT-PCR of d50 ARC cultures ± BMP7.
(J) qRT-PCR and (K) ICC of d50 ARC with d25-50 BMP4 or BMP7 (n=3 RC17 hESC). Dotted line is average of BMP7-treated samples, mean ± SEM.

(L) qRT-PCR of day 50 ARC cultures ± IGFBP3.

Statistics: (B) *NKX2-1* (One-way ANOVA with Tukey's test): VFB vs. No BMP p = 0.0002; VFB vs. BMP4 p = 0.0003; VFB vs. PVN p < 0.0001, *RAX* (Kruskal-Wallis with Dunn's test): VFB vs. BMP p = 0.0006; No BMP vs. BMP p = 0.0156, *TBX3*: VFB vs. BMP p = 0.0280; No BMP vs. BMP p = 0.0341; BMP vs. PVN p = 0.0225, *SIM1*: BMP4 vs. PVN p = 0.0079. *FOXG1*: VFB vs. PVN p = 0.0010; No BMP vs. PVN p = 0.0466

(D) BMP4 vs. BMP7: Unpaired t-test; ns, not significant (I) *POMC*: Paired t-test, p = 0.0154; n = 5, TBX3: Paired t-test, p = 0.0043; n = 4 (J) Wilcoxon matched-pairs signed rank test with two-stage step up of Benjamini, Krieger and Yekutieli; all ns. (L) *AGRP*: Wilcoxon matched-pairs signed rank test, n = 6, p = 0.0563 POMC: p = 0.0312

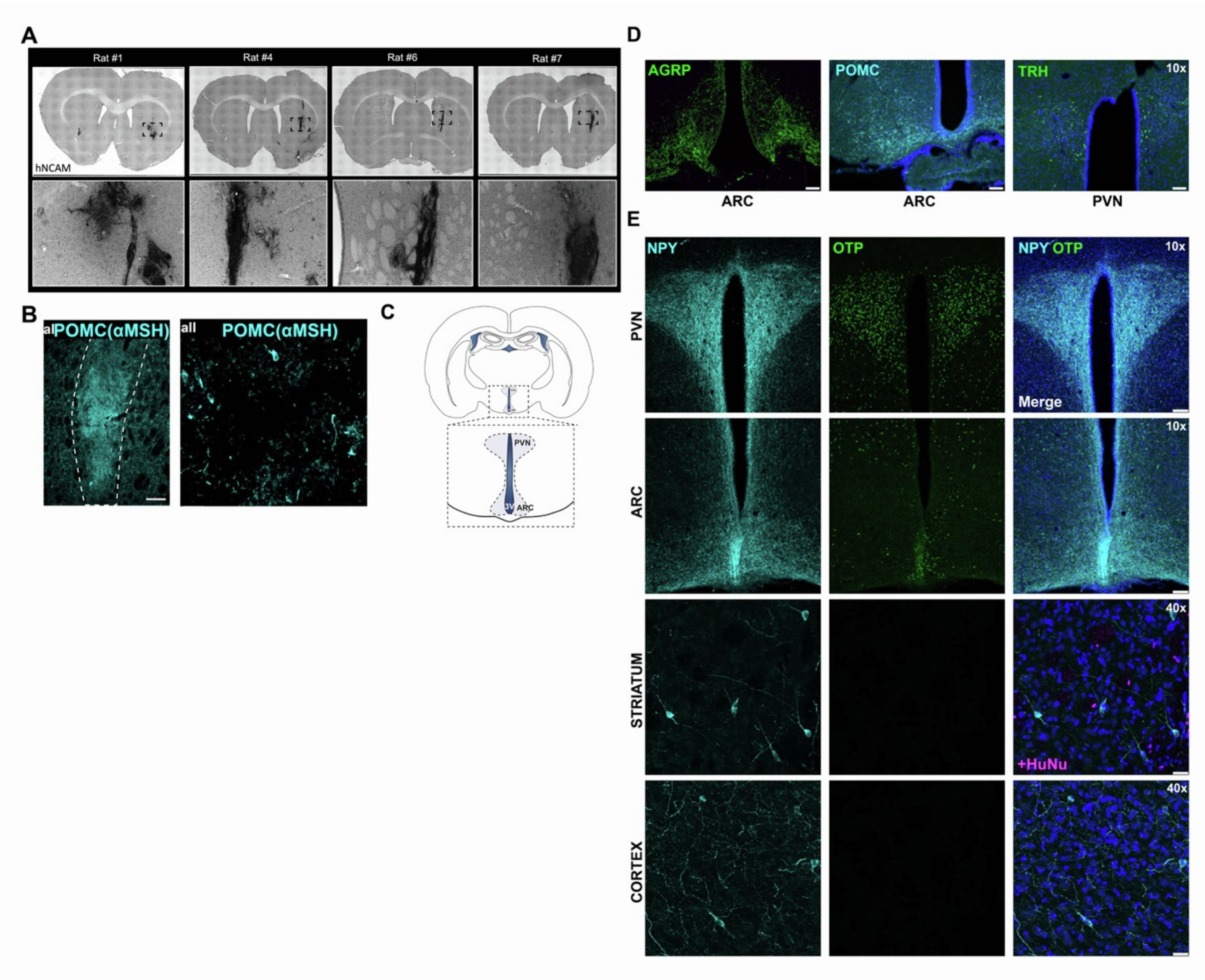

Supplementary Figure 2. Validation of ARC graft identity and NPY expression *in vivo*, related to Figure 1.

(A) Xenografts fixed for IHC from n=4 animals showing hNCAM labelling human neurons in the rat striatum.

(B) ICC showing POMC (αMSH) cells within the xenograft (aI) and at higher magnification (aII). Dotted line denotes graft boundary. Scale bars: 200 μm (aI), 25 μm (aII).

(C) Schematic of a coronal rat brain section depicting the anatomical positions of the ARC and PVN.

(D) Antibody staining for AGRP, POMC, and TRH in native rat hypothalamic sections to validate antibody specificity. Scale bars: 100 μm.

(E) Antibody staining for NPY and OTP in rat brain sections. ARC neurons show distinct NPY localization and morphology compared to NPY
interneurons in the striatum and cortex. Scale bars: 200 μm (ARC, PVN), 50 μm (striatum, cortex)

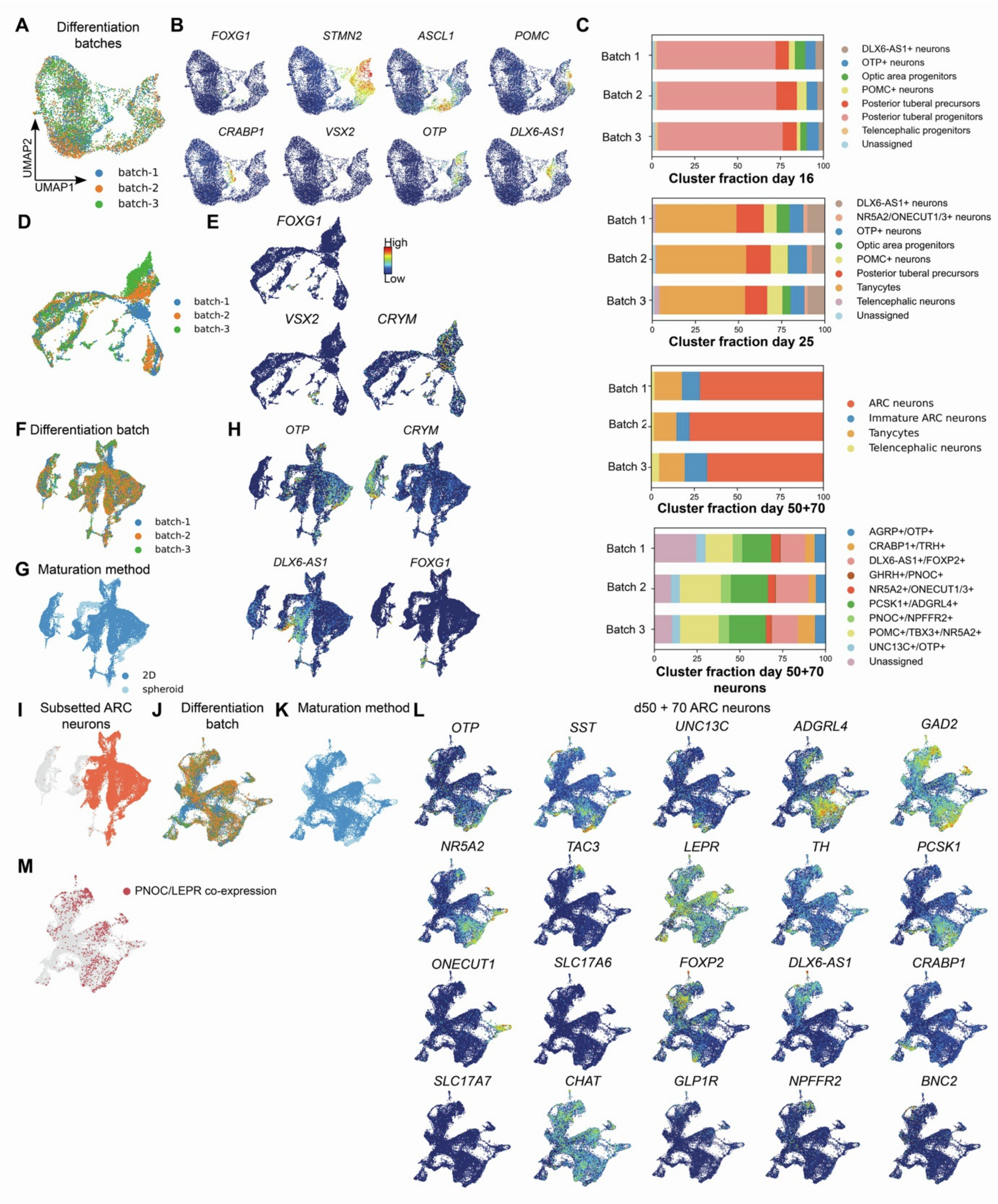

Supplementary Figure 3. Single-cell and single-nucleus profiling of ARC cultures across developmental timepoints, related to Figure 2.
(A) UMAP of scRNA-seq data from d16 ARC cultures (n = 3; RC17) colored by replicates (batches).
(B) Feature plots of key marker genes in the d16 dataset.
(C) Bar plots showing the distribution of cell types across d16 (top), and later timepoints (d25, d50+70, day 50+70 neurons; bottom) (n = 3; RC17).
(D) UMAP of d25 snRNA-seq data colored by differentiation batch (n = 3; RC17).
(E) Feature plots of markers for tanycytes, telencephalic, and eye field progenitors.
(F) UMAP of d50+70 snRNA-seq data colored by differentiation batch (n = 3; RC17).
(G) UMAP of d50+70 snRNA-seq data colored by 2D versus 3D maturation conditions.
(H) Feature plots of key markers from d50+70 ARC snRNA-seq data.
(I) UMAP showing ARC neuron cluster subsetted from d50+70 dataset.
(J) UMAP of ARC neurons at d50+70 colored by differentiation batch (n = 3; RC17).

(K) UMAP of ARC neurons at day 50+70 colored by maturation condition (2D vs. 3D).
(L) Feature plots of selected marker genes in day 50+70 ARC neurons.
(M) UMAP of neuronal cells co-expressing *PNOC* and *LEPR* in the day 50+70 dataset.

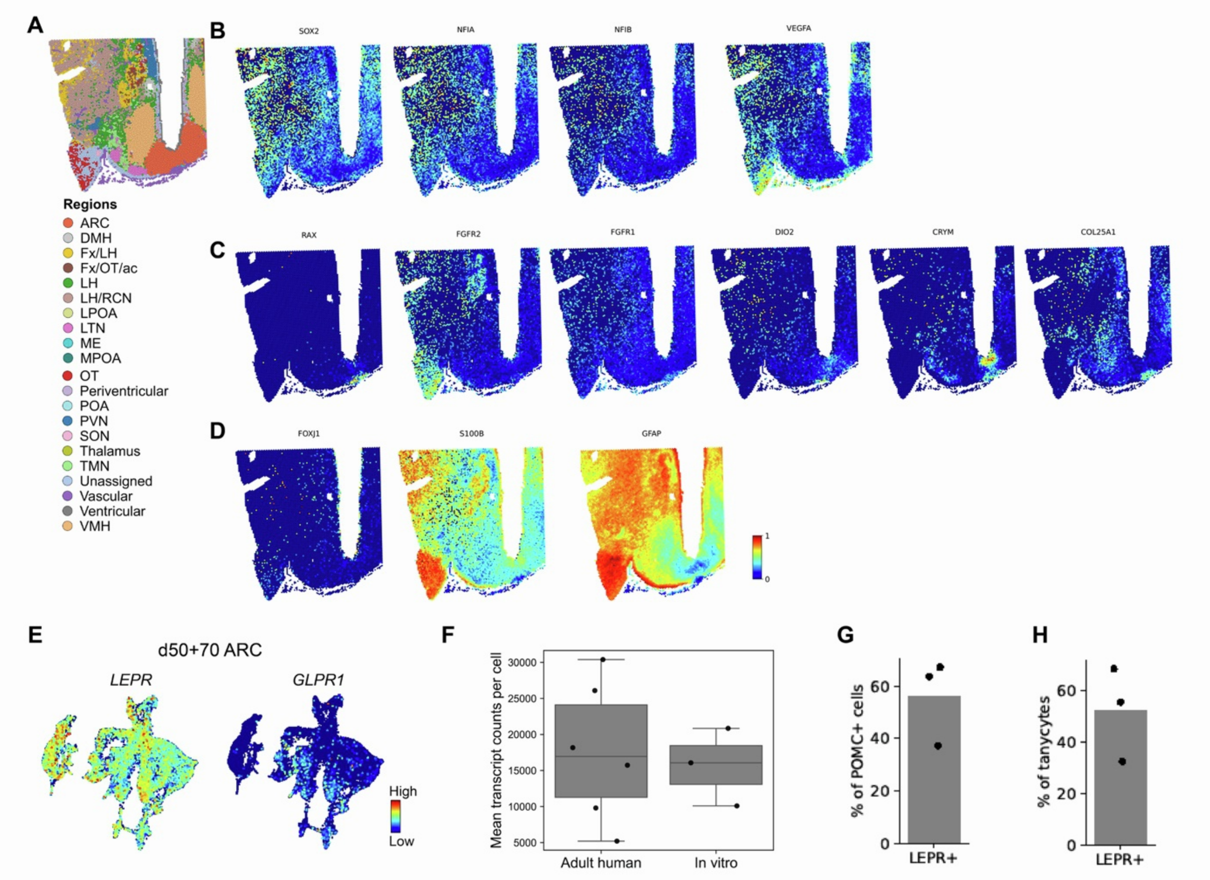

Supplementary Figure 4. Spatial transcriptomics of tanycyte marker and cluster-specific *LEPR* expression, related to Figure 4.

(A) Spatial transcriptomics tissue-section plot from adult human HYPOMAP[2] showing regional annotations.

(B-D) Spatial HYPOMAP[2] showing expression patterns for general (B), β (C), and α (D) tanycyte markers.

(E) UMAPs of *LEPR* and *GLP1R* at d50+70, annotations in Figure 2E.

(F) Tukey's box plot comparing mean transcript counts per cell between adult human HYPOMAP[2] and d50+70 ARC scRNA-seq data (from Figure 2E). Each dot represents a donor in HYPOMAP data and a differentiation batch in *in vitro data.*

(G) Bar plot quantifying co-expression of POMC and LEPR in POMC+ neurons (data from Figure 2G). Each dot represents a differentiation batch.

(H) Bar plot quantifying LEPR expression in tanycytes (data from Figure 2E). Each dot represents a differentiation batch.

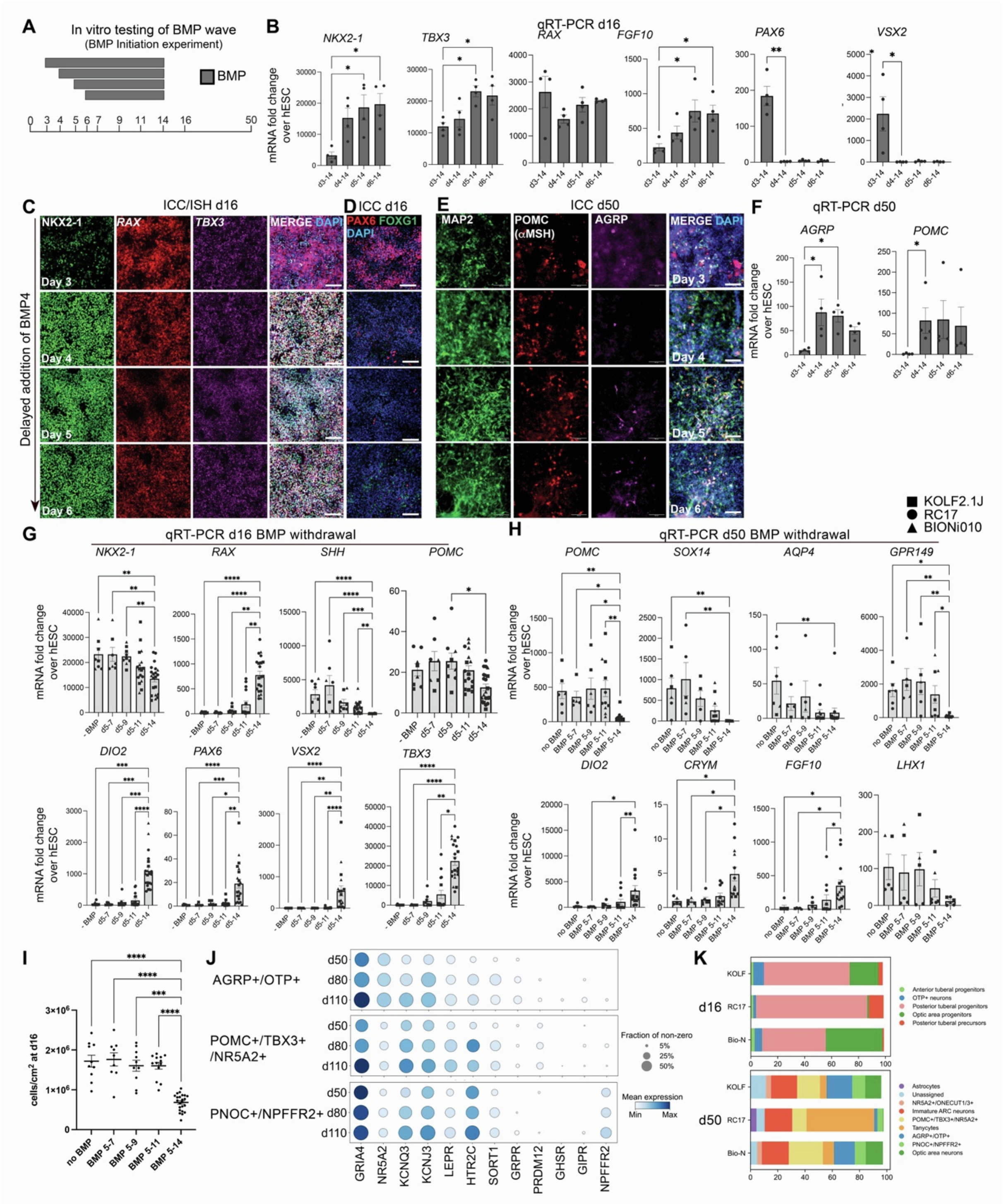

Supplementary Figure 5. Early BMP4 exposure timing modulates ARC patterning and generated neurons, related to Figure 5.

(A) Experimental timeline of BMP4 addition from either day 3, 4, 5, or 6 until day 14.

(B) qRT-PCR analysis of day 16 cultures (n = 4) patterned with different BMP4 start times.

(C) ICC for NKX2-1 and ISH for *RAX* and *TBX3* on day 16 ARC cultures exposed to BMP4 at different initiation points. Scale bar: 100 µm.

(D) ICC of FOXG1 and PAX6 expression at day 16 under various BMP4 exposure timings. Scale bar: 100 µm.

(E) ICC of POMC (αMSH), AGRP, and MAP2 expression in day 50 ARC cultures treated with different BMP4 start times. Scale bar: 100 µm.

(F) qRT-PCR of day 50 ARC cultures (n = 4) treated with BMP4 from day 3–6 until day 14.

(G) qRT-PCR of three cell lines at day 16 with BMP4 added from day 5 to day 7, 9, 11, or 14.

(H) qRT-PCR of three cell lines at day 50 under same BMP4 conditions.

(I) Cell counts of differentiations (RC17, KOLF2.1J, BIONi010) after dissociation at d16 treated with BMP4 for different timepoints.
(J) Dot plots showing trend towards increased expression of selected genes in ARC clusters over a time course spanning three timepoints from day 50 to 110 (n=1, RC17 hESC, BMP4 5-14).
(K) Bar plots showing distribution of cell types across cell lines in BMP4 5-14 snRNA-seq data (from Figure 5F-G) at d16 and d50.
Statistics: All bar plots and dot plots represent mean ± SEM. (B) One-way ANOVA with Tukey's test: NKX2-1: d3–14 vs. d5–14 p = 0.0211; d3–14 vs. d6–14 p = 0.0141. *TBX3*: d3–14 vs. d5–14 p = 0.0208; d3–14 vs. d6–14 p = 0.0432. *FGF10*: d3–14 vs. d5–14 p = 0.0293; d3–14 vs. d6–14 p = 0.0437. Kruskal-Wallis: *RAX*: ns; PAX6: d3–14 vs. d4–14 p = 0.0050; *VSX2*: d3–14 vs. d4–14 p = 0.0140 (F) *AGRP* (ANOVA): d3–14 vs. d4–14 p = 0.0190; d3–14 vs. d5–14 p = 0.0332.*POMC* (Kruskal-Wallis): d3–14 vs. d4–14 p = 0.0227. (G) *NKX2-1* (ANOVA): No BMP vs. d5–14 p = 0.0038; d5–7 vs. d5–14 p = 0.0069; d5–9 vs. d5–14 p = 0.0067 Kruskal-Wallis: *RAX*: No BMP vs. d5–14 p < 0.0001; d5–7 vs. d5–14 p = 0.0004; d5–9 vs. d5–14 p = 0.0055; d5–11 vs. d5–14 p = 0.0074. *SHH*: No BMP vs. d5–14 p < 0.0001; d5–7 vs. d5–14 p < 0.0001; d5–9 vs. d5–14 p = 0.0006; d5–11 vs. d5–14 p = 0.0016. *POMC*: d5–9 vs. d5–14 p = 0.0273. *DIO2*: No BMP vs. d5–14 p = 0.0002; d5–7 vs. d5–14 p = 0.0001; d5–9 vs. d5–14 p = 0.0002; d5–11 vs. d5–14 p < 0.0001. *PAX6*: No BMP vs. d5–14 p < 0.0001; d5–7 vs. d5–14 p = 0.0004; d5–9 vs. d5–14 p = 0.0352; d5–11 vs. d5–14 p = 0.0018. *VSX2*: No BMP vs. d5–14 p < 0.0001; d5–7 vs. d5–14 p = 0.0013; d5–9 vs. d5–14 p = 0.0011; d5–11 vs. d5–14 p < 0.0001. (H) Kruskal-Wallis: *POMC*: No BMP vs. d5–14 p = 0.0068; d5–7 vs. d5–14 p = 0.0205; d5–9 vs. d5–14 p = 0.0124; d5–11 vs. d5–14 p = 0.0012. *SOX14*: No BMP vs. d5–14 p = 0.0025; d5–7 vs. d5–14 p = 0.0045. *AQP4*: No BMP vs. d5–14 p = 0.0060. *GPR149*: No BMP vs. d5–14 p = 0.0108; d5–7 vs. d5–14 p = 0.0045; d5–9 vs. d5–14 p = 0.0078; d5–11 vs. d5–14 p = 0.0454. *DIO2*: d5–7 vs. d5–14 p = 0.0268; d5–11 vs. d5–14 p = 0.0070. *CRYM*: No BMP vs. d5–14 p = 0.0140; d5–7 vs. d5–14 p = 0.0354; d5–9 vs. d5–14 p = 0.0364. *FGF10*: No BMP vs. d5–14 p = 0.0139; d5–7 vs. d5–14 p = 0.0362; d5–11 vs. d5–14 p = 0.0229. (I) Kruskal-Wallis: no BMP bs. BMP5-14 p<0.0001; BMP5-7 vs. BMP5-14 p<0.0001; BMP5-9 vs. BMP5-14 p = 0.0003; BMP5-11 vs. BMP5-14 p<0.0001.

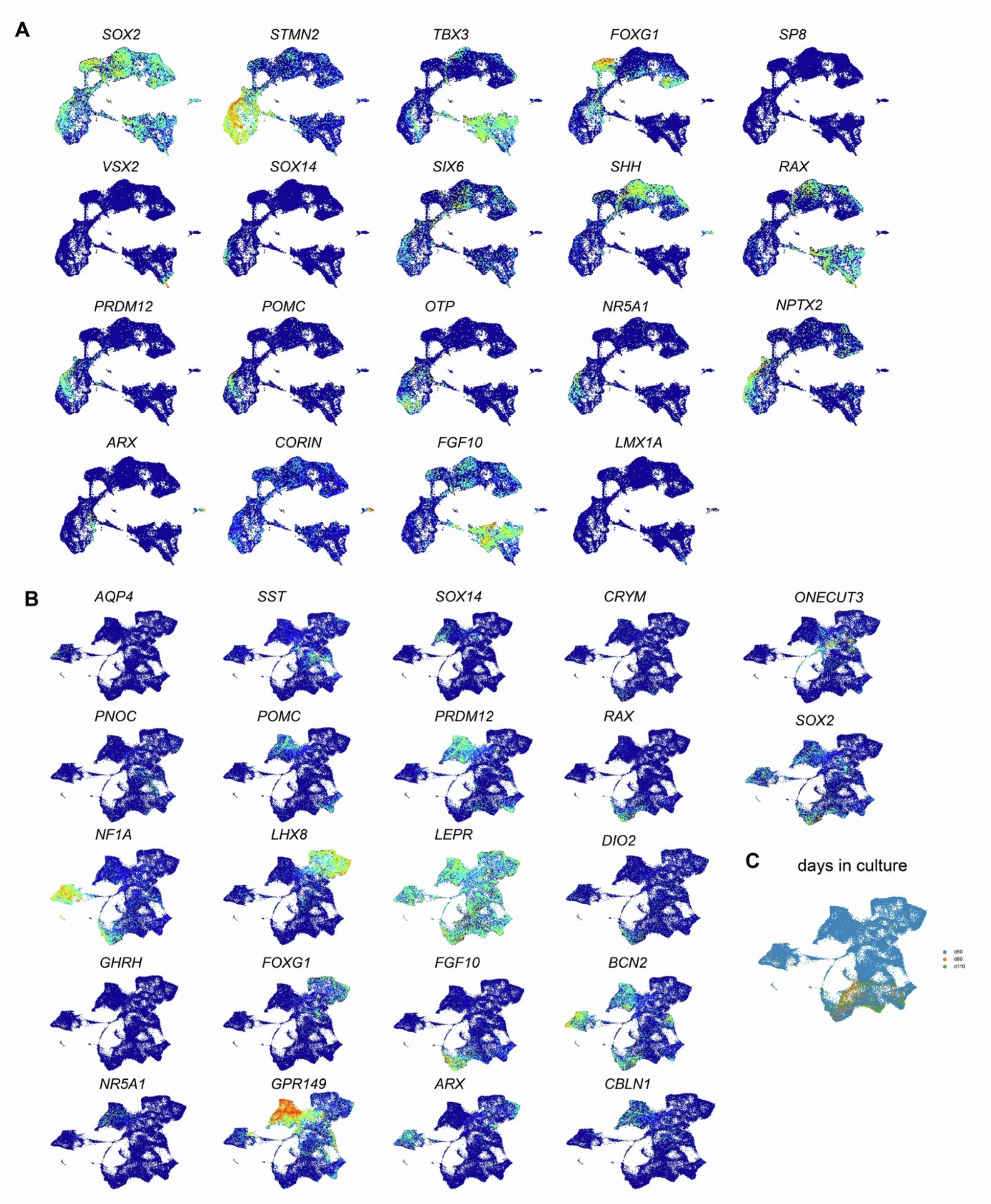

Supplementary Figure 6. Single-cell transcriptomic analysis of BMP4 withdrawal on ARC cultures, related to Figure 5.
(A) Feature plots of key hypothalamic patterning markers in the d16 dataset under different BMP4 conditions.
(B) Feature plots of relevant hypothalamic and lineage-specific markers in d50+80+110 ARC cultures patterned under different BMP4 regimens.
(C) UMAP of d50+80+110 cultures colored by timepoint.

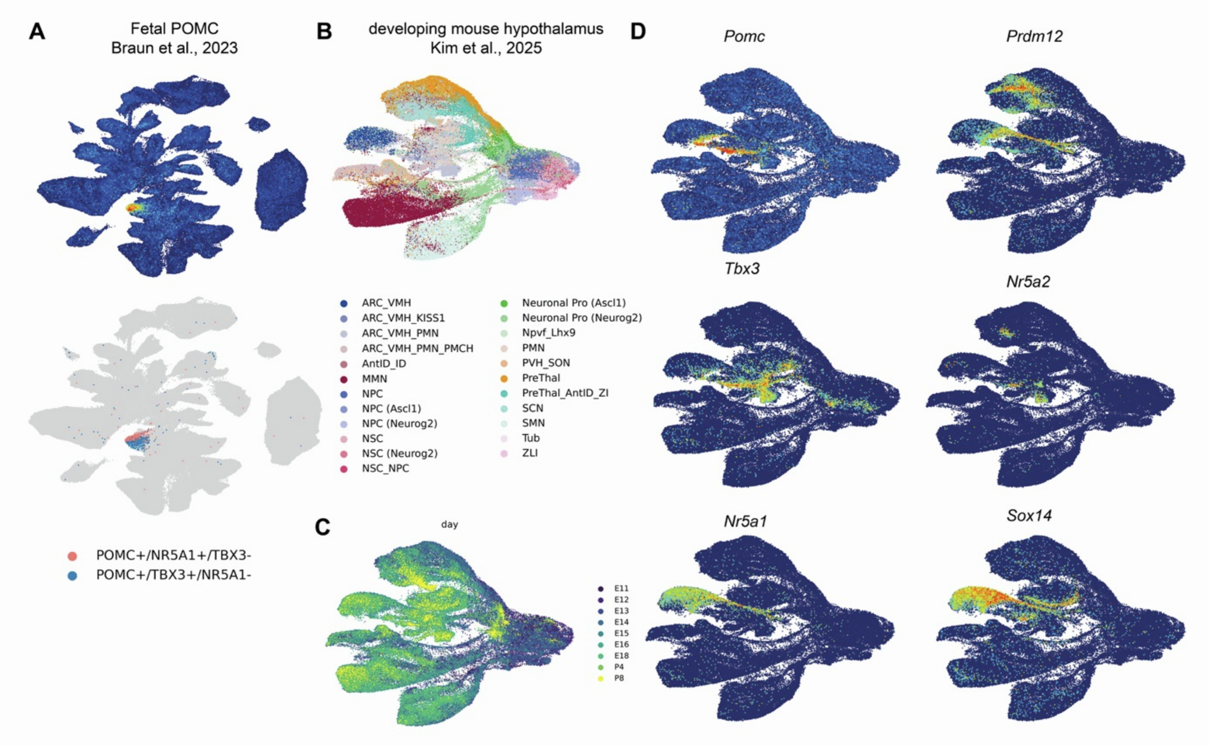

Supplementary Figure 7. Developmental origin of distinct POMC clusters, related to Figure 6.
(A) Top: UMAP of the developing human brain atlas (trimester 1) from Braun *et al.*, 2023[3] showing spatial expression of POMC across early brain regions. Bottom: UMAP showing two distinct POMC-expressing clusters identified by co-expression analysis in the fetal brain dataset, corresponding to ARC- and VMH-like populations.
(B) UMAP showing cluster annotations in the developing mouse hypothalamus[3].
(C) UMAP showing developmental time points in the developing mouse hypothalamus.
(D) UMAP showing gene expression patterns of POMC and regional ARC and VMH markers in the developing mouse hypothalamus.

Supplementary tables

Gene symbol	Gene name	Forward primer sequence	Reverse primer sequence
ACTB	Actin beta	ATGTGGCCGAGGACTTTGATTG	ATGGCAAGGGACTTCCTGTAAC
AGRP	Agouti related neuropeptide	TCCCTGTCCTGTGGAAATTTGT	TCTCCAATTTGGGGTGAGGTTT
AQP4	Aquaporin 4	TTGGACCTGCAGTTATCATGGG	CTATGATGGGCCCAACCCAATA
ARX	Aristaless related homeobox	CCTGAGCACTTTCCTCGGAGCG	TGGAAAAGAGCCTGCCGAATGCC
BRN2	POU3F2, POU class 3 homeobox 2	ATGCGCGGCTCCTTTAACCGG	TTAGACGCTGCGGTCGCCATG
BSX	Brain specific homeobox	GAGAAGAGGTTCGAGATCCAGC	GTTCTGGAACCACGTTTTCACC
CART	CART prepropeptide	GCGTCCATTCTCCTCCATACAT	GTTGCTTAAGCCAAACTCCAGG
CRH	Corticotropin releasing hormone	CTGTACCATAGCGCTGCTCTTA	TTGTGCATGCTAAGTAAGGGGT
CITED1	Cbp/p300 interacting transactivator with Glu/Asp rich carboxy-terminal domain 1	AGTGGATGAGGAAGTGCTGATG	AAGTCAAACTCATTCTGCCCCA
COL1A1	Collagen type I alpha 1 chain	CTTATGAAACCCCAATGCTGCC	GGGAGACAGATTTGGGAAGGAG
COL25A1	Collagen type XXV alpha 1 chain	GACCCAGGTATGACAGGTGAAAA	CATTCCATTGGCTCCCGGTAA
CRYM	Crystallin mu	ACAGAGCCCATTTTGTTTGGTG	CATCCAGTTCTCTCCAGTCAGG
DBX1	Developing brain homeobox 1	GAAGTTTGGAGTGAACGCCATC	GACCCTTCGAAGTAGGGAAAGG
DIO2	Iodothyronine Deiodinase 2	GTTATAAGGCAACCCCCGGTAT	AACCTCAGCTAATGGGACCAAG
DLX2	Distal-less homeobox 2	ACCAGACCTCGGGATCCGCC	CTGCGGGGTCTGAGTGGGGT
EN1	Engrailed homeobox 1	CGTGGCTTACTCCCCATTTA	TCTCGCTGTCTCTCCCTCTC
FEZF1	FEZ family zinc finger 1	GGTACATTCCACATTCGTGAGC	TCACGTGCAATAATCAAAACCA
FGF10	Fibroblast growth factor 10	GTTGCTGTTCTTGGTGTCTTCC	TGACACCATGTCCTGACCAAG
FOXA1	Forkhead box A1	GGGCAGGGTGGCTCCAGGAT	TGCTGACCGGGACGGAGGAG
FOXA2	Forkhead box A2	CCGTTCTCCATCAACAACCT	GGGGTAGTGCATCACCTGTT
FOXB1	Forkhead box B1	GTGGTCGGACTTAAGCACCTT	GTGGTCGGACTTAAGCACCTT
FOXD1	Forkhead box D1	CCTGTCCAGTGTCGAGAACTTT	AACCACCAAGACGAGAAAAGGA
FOXG1	Forkhead box G1	TCAACGGCATCTACGAGTTCAT	AAGCACTTGTTGAGGGACAGAT

GAPDH	Glyceraldehyde-3-phosphate dehydrogenase	TTGAGGTCAATGAAGGGGTC	GAAGGTGAAGGTCGGAGTCA
GBX2	Gastrulation brain homeobox 2	GTTCCCGCCGTCGCTGATGAT	GCCGGTGTAGACGAAATGGCCG
GHRH	Growth hormone releasing hormone	AGGAACTCCCAGGGATGAAGAT	CAGTTGCATTTTGGCTACAGGT
GRH	Growth hormone receptor	ACTAGCAATGGTGGTACAGTGG	TCAGTAAAGTCCAGTTGAGGGC
GPR149	G protein-coupled receptor 149	GACTGGGAGTGGTGTAGGAGTA	GGCATAACCGGAACGCTGAC
HCRT	Hypocretin neuropeptide predursor	CCTCAAGGTTCCTGGCTTTTTG	GGAAGGAAGGTTCATGGTGTCT
ISL1	ISL LIM homeobox 1	AAGCGCAGGAAGAGAGACTG	CCAAGAGACCCAGGATTTCA
KISS1	KiSS-1 metastasis suppressor	TGAACTTCAGACCCCAAAGGAG	TCTTTTATTGCCTCGGGTTGGA
LEPR	Leprin receptor	TACTGTTACGGTTCTGGCCATC	TGCTCATAGGCCATGAAAAGGT
LHX2	LIM homeobox 2	GGGCGACCACTTCGGCATGAA	CGTCGGCATGGTTGAAGTGTGC
LHX6	LIM Homeobox 6	AGGCAAGAACATCTGCTCCAG	GCCAGATGAGGTTGTTGACCTT
LHX8	LIM Homeobox 6	AGGCAAGAACATCTGCTCCAG	GCCAGATGAGGTTGTTGACCTT
LHX9	LIM Homeobox 9	TGCCTGAAGTGCTGTGAATGTA	TTGCAGTAAATGCTACCGTCCT
MASH1	ASCL1, achaete-scute family bHLH transcription factor 1	CTAAAGATGCAGGTTGTGCG	GGAGCTTCTCGACTTCACCA
MC4R	Melancortin 4 receptor	CTTTTTCATCTGCAGCTTGGCT	TGTGAAACTCTGTGCATCCGTA
MCH	Melanin concentrating hormone	TACATTCAGGTTGGGGAAAGGC	CCAGGGAAGGAGCAATAACTGA
MPZ	Myelin protein zero	CTCAGGTCACGCTGTATGTCTT	GAACCACGTAGAAAAGCAGCAG
NCAM1	Neural cell adhesion molecule 1	GTCAGAGGCCACCGTCAACGTG	CTTCCCCCTCCCGGAACTCCTG
NGN3	Neurogenin 3	CTGAACTTGGCGACCAGAAGC	TTGAGGCGTCATCCTTTCTACC
NHLH2	Nescient helix-loop-helix 2	ACCCACTGGAGACTTTGAGTTC	GCATACTCTGAACTTCTGCCCT
NKX2.1	NK2 homeobox 1	AGGGCGGGGCACAGATTGGA	GCTGGCAGAGTGTGCCCAGA
NPY	Neuropeptide Y	GAAAATGTTCCCAGAACTCGGC	TAGGAAAAGGCCAGAGAGCAAG
NR5A1	Nuclear receptor subfamily 5 group A member 1	GGAACAAGTTTGGGCCGATGTA	GTGCCTTCTTCTGCTGTTTCAG
NROB1	Nuclear receptor subfamily 0 group B member 1	GCCATCAAGTGCTTTCTTTCCA	TAGGCGTACTCCTTGGTACTGA
NTS	Homo sapiens neurotensin	GCTCAAAGTACTACAGCAAAGCC	GGCGCTATTACTTTGTTTTGGGT
ONECUT2	One cut homeobox 2	AAGGGGTAGAGCTGGTGTATCT	TGTTGTTTCAGGGGTGACTTGA

OTP	Orthopedia homeobox	TAGAAGGGAAGGC TTCTCAGGA	GTCAGATCACCTCTTC CTCGTC
OTX2	Orthodenticle homeobox 2	ACAAGTGGCCAAT TCACTCC	GAGGTGGACAAGGGA TCTGA
PAX6	Paired box 6	TGGTATTCTCTCC CCCTCCT	TAAGGATGTTGAACGG GCAG
PCSK1	Proprotein convertase subtilisin/kexin type 1	GCAATGCCCGTAA TGCTTAGAG	TTCCAAGGACAGAGT GATTCCG
PCSK2	Proprotein convertase subtilisin/kexin type 2	GATTGACTATCTCC ACCCGGAC	TCTGTGTACCGAGGG TAAGGAT
PDYN	Prodynorphin	CTCATTCCCAGGC ACTCTCTTT	TTTCCTCTCCTATCCA GCCTCA
PITX2	Paired like homeodomain 2	AACTCTATGAACG TCAACCCCC	CGACATGCTCATGGA CGAGATA
POMC	Proopiomelanocortin	TTTCATGACCTCC GAGAAGAGC	GATGATGGCGTTTTTG AACAGC
PNOC	Prepronociceptin	AGTGTGTTCAGCA GTTGTCAGA	ACCTTCTCTTCACACT CGAGGA
PRDM12	PR/SET domain 12	GTGGGAGGTGTTC AATGAGGAT	GTTCGTTACGTGCACA CTTGAT
PVALB	Parvalbumin	GGACAAGGACAAA AGTGGCTTC	CAGCCATCAGCATCTT GGTTTC
RAX	Retina and anterior neural fold homebox	CCTCTCAGTTCAC CAAGCAGAT	TGATCAACCTTGGGT GTTAGGG
RFX4	Regulatory factor X4	ACCTTGCCATCTG TCTTGTCAT	ATAGGGATGGTACCAC CAGGAA
SHH	Sonic hedgehog	CCAATTACAACCC CGACATC	AGTTTCACTCCTGGC CACTG
SIM1	Single-minded family bHLH transcription factor 1	AAAGGGGGCCAA ATCCCGGC	TCCGCCCCACTGGCT GTCAT
SIM2	SIM bHLH transcription factor 2	GGCTACTTGAAGA TCAGGCAGT	ATCTGGTAGCAGGAG TCGTACA
SIX3	SIX homeobox 3	ACCGGCCTCACTC CCACACA	CGCTCGGTCCAATGG CCTGG
SIX6	SIX homeobox 6	CTCAACAAGAATG AGTCGGTGC	ACTCCTTGGTGAACTT GTGGTT
SOX14	SRY-box 14	CATACATCGATGAA GCCAAGCG	CTGTCCTTCTTGAGCA GGTTCT
SP8	Sp8 transcription factor	CCTGTCTGTCCGG ACTTCAA	AGGGGCAGAAACAGA AAGAGAC
SP9	Sp9 transcription factor	TCTGGCCCCAACG ACTCTTAG	CTCGTTCGTTCTCGGT GTCTC
SST	Somatostatin	GGAACCTGAAGAT CTGTCCCAG	ATAGCCGGGTTTGAGT TAGCAG
TBX3	T-box transcription factor 3	GGGGGTAGGAGTT CCAACATTT	GCACTGAGGGAGATG TCTTTGA
TH	Tyrosine hydroxylase	CGGGCTTCTCGGA CCAGGTGTA	CTCCTCGGCGGTGTA CTCCACA
TRH	Thyrotropin releasing hormone	TCCTGGATGACCT GAGTAGGAG	TCAGGGAAAACTGGG TTCACTC
VSX2	Unc-5 netrin receptor D	CCTCTGCCCTCTG TAAATGTGT	AGAGACCTCTGCGAG AACTTTG

Supplementary Table 1: List of qRT-PCR primers, related to STAR methods.

Antigen	Host species	Dilution	Manufacturer (#Cat)	Reacts with human?	Validated in rat in-house?
NKX2-1	Rabbit	1:100-200	Abcam (#ab1333737)	yes	
PAX6	Mouse	1:1000	Sigma-Merck (#AMAb91372)	yes	
AGRP	Rabbit	1:1000	Phoenix Pharma (#H-003-53) – discontinued	yes	yes
AGRP	Goat	1:100-200	R&D Systems (#AF634)		yes
POMC (aMSH)	Sheep	1:1000	Millipore (#AB5087)	Unknown, “mammals”	yes
MAP2	Mouse	1:1000	Sigma (#M1406)	yes	
TRH	Rabbit	1:1000	Thermo Fisher (PA5-57331)	yes	yes
TH	Mouse	1:1000	Immunostar (173-22941)	yes	yes
GHRH	Rabbit	1:200	Abcam (ab18751)	yes	
OTP	Rabbit	1:500	GeneTex (GTX119601)	yes	yes
FOXG1	Rabbit	1:500	Abcam (#ab18259)	yes	
CRH	Rabbit	1:1000	Proteintech (10944-1-AP)	yes	
AQP4	Rabbit	1:1000	Sigma-Merck (#HPA014784)	yes	yes
hNCAM1	Mouse	1:1000	Santa Cruz Biotechnology #SC-106	yes	
HuNu	Mouse	IF: 1:1000 ISH: 1:200	Millipore #AB1281	yes	yes
NPY	Sheep	1:500	Millipore #AB1583		yes
S100b	Mouse	1:1000	Sigma (#S2532)	yes	yes
Somatostatin	Mouse coupled-Alexa Fluor 488	1:500	BD Biosciences (#566032)	yes	yes
NFIA	Rabbit	1:200	Abcam (#ab228897)	yes	yes
Vimentin	Chicken	1:500	Millipore (#AB5733)	yes	
NR5A1	Mouse	1:1000	Thermo Fisher (#434200)	yes	yes

HuC/D	Mouse	1:400	Invitrogen (A-21271)	yes
bMSH	Rabbit		LSBio #LS-C183969-50)	yes

Supplementary Table 2: List of primary antibodies, related to STAR methods.

ANTIGEN	FLUOROPHORE	DILUTION	MANUFACTURER (#CAT)
RABBIT	Alexa488	1:200-500	Jackson Imm. Res. (#711-545-152)
MOUSE	Alexa647	1:200-500	Jackson Imm. Res. (#711-605-151)
SHEEP	Alexa Cy3	1:200-500	Jackson Imm. Res. (#713-165-147)
MOUSE	Alexa Cy3	1:200	Jackson Imm. Res. (#715-165-151)
MOUSE	Alexa 488	1:200-500	Jackson Imm. Res. (#715-545-150)
RABBIT	Alexa CY3	1:200	Jackson Imm. Res. (#711-165-152)
GOAT	Alexa488	1:200-500	Jackson Imm. Res. (#705-545-147)
SHEEP	Alexa647	1:200-500	Jackson Imm. Res. (#713-605-147)
GOAT	Alexa647	1:200	Jackson Imm. Res. (#705-605-147)
MOUSE	Biotinylated	1:500	Vector Laboratories #BA9200
DAPI	-	1:500	Thermo (#D3571)

Supplementary table 3. List of secondary antibodies, related to STAR methods.

Probe	Host species	Dilution	Manufacturer (#Cat)
Hs-RAX-C1	Homo sapiens	Ready-to-use	ACD Bio-Techne (#579951)
Hs-TBX3-C2	Homo sapiens	1:50	ACD Bio-Techne (#557441)
Hs-PNOC-C2	Homo sapiens	1:50	ACD Bio-Techne (#1045241)
ISL1	Chicken	4-10nM	Molecular Instruments
SIX6	Chicken	4-10nM	Molecular Instruments
POMC	Chicken	4-10nM	Molecular Instruments
**Fluorophore**			
Opal *480*	1:500	Akoya Bio FP1500001KT	
Opal *570*		1:500	Akoya Bio FP1488001KT
Opal *690*		1:500	Akoya Bio FP1497001KT
HCR v3.0 amplifier B1			Molecular Instruments Alexa Fluor 488
HCR v3.0 amplifier B3			Molecular Instruments Alexa Fluor 647
HCR v3.0 amplifier B4			Molecular Instruments Alexa Fluor 546

Supplementary table 4. List of RNAscope probes and fluorophores, related to STAR methods.

10X reaction ID	Approach	Sample	Day	Cells before filtering	Cells after filtering
D4	scRNA-seq	BATCH-0 ARC d16, BATCH-1 ARC d16, BATCH-2 ARC d16, BATCH-3 ARC d16	day 16	10939	8392
D1	snRNA-seq	BATCH-0_D25, BATCH-1_D25, BATCH-2_D25, BATCH-3_D25, BATCH-0_D50, BATCH-0_D70, BATCH-0_D50_organoid, BATCH-0_D70_organoid	day 25, 50, 70	5458	3639
E1	snRNA-seq	BATCH-0_D25, BATCH-1_D25, BATCH-2_D25, BATCH-3_D25, BATCH-0_D50, BATCH-0_D70, BATCH-0_D50_organoid, BATCH-0_D70_organoid	day 25, 50, 70	6036	3905
F1	snRNA-seq	BATCH-0_D25, BATCH-1_D25, BATCH-2_D25, BATCH-3_D25, BATCH-0_D50, BATCH-0_D70, BATCH-0_D50_organoid, BATCH-0_D70_organoid	day 25, 50, 70	5534	3646
G1	snRNA-seq	BATCH-1_D50_CNT, BATCH-1_D50_control1,	day 50	7523	4637

		BATCH-1_D50_control2, BATCH-1_D50_control3, BATCH-1_D50_control4, BATCH-1_D50_FGF1, BATCH-1_D50_organoid			
H1	snRNA-seq	BATCH-1_D50_CNT, BATCH-1_D50_control1, BATCH-1_D50_control2, BATCH-1_D50_control3, BATCH-1_D50_control4, BATCH-1_D50_FGF1, BATCH-1_D50_organoid	day 50	7231	4763
C2	snRNA-seq	BATCH-1_D50_CNT, BATCH-1_D50_control1, BATCH-1_D50_control2, BATCH-1_D50_control3, BATCH-1_D50_control4, BATCH-1_D50_FGF1, BATCH-1_D50_organoid	day 50	6838	4348
D2	snRNA-seq	BATCH-2_D50_CNT, BATCH-2_D50_control1, BATCH-2_D50_control2, BATCH-2_D50_control3, BATCH-2_D50_control4, BATCH-2_D50_FGF1, BATCH-2_D50_organoid	day 50	5771	3232
E2	snRNA-seq	BATCH-2_D50_CNT,	day 50	6620	3041

		BATCH-2_D50_control1, BATCH-2_D50_control2, BATCH-2_D50_control3, BATCH-2_D50_control4, BATCH-2_D50_FGF1, BATCH-2_D50_organoid			
F2	snRNA-seq	BATCH-2_D50_CNT, BATCH-2_D50_control1, BATCH-2_D50_control2, BATCH-2_D50_control3, BATCH-2_D50_control4, BATCH-2_D50_FGF1, BATCH-2_D50_organoid	day 50	6432	3514
G2	snRNA-seq	BATCH-3_D50_CNT, BATCH-3_D50_control1, BATCH-3_D50_control2, BATCH-3_D50_control3, BATCH-3_D50_control4, BATCH-3_D50_FGF1, BATCH-3_D50_organoid	day 50	5173	1691
H2	snRNA-seq	BATCH-3_D50_CNT, BATCH-3_D50_control1, BATCH-3_D50_control2, BATCH-3_D50_control3, BATCH-3_D50_control4, BATCH-3_D50_FGF1, BATCH-3_D50_organoid	day 50	4194	1557

A6	snRNA-seq	BATCH-1_D70_CNT, BATCH-1_D70_control1, BATCH-1_D70_control2, BATCH-1_D70_control3, BATCH-1_D70_control4, BATCH-1_D70_FGF1, BATCH-1_D70_organoid	day 70	4845	1043
A7	snRNA-seq	BATCH-1_D70_CNT, BATCH-1_D70_control1, BATCH-1_D70_control2, BATCH-1_D70_control3, BATCH-1_D70_control4, BATCH-1_D70_FGF1, BATCH-1_D70_organoid	day 70	4075	1018
A9	snRNA-seq	BATCH-1_D70_CNT, BATCH-1_D70_control1, BATCH-1_D70_control2, BATCH-1_D70_control3, BATCH-1_D70_control4, BATCH-1_D70_FGF1, BATCH-1_D70_organoid	day 70	3910	958
A11	snRNA-seq	BATCH-2_D70_CNT, BATCH-2_D70_control1, BATCH-2_D70_control2, BATCH-2_D70_control3, BATCH-2_D70_FGF1, BATCH-2_D70_organoid	day 70	2683	1018

B5	snRNA-seq	BATCH-2_D70_CNT, BATCH-2_D70_control1, BATCH-2_D70_control2, BATCH-2_D70_control3, BATCH-2_D70_FGF1, BATCH-2_D70_organoid	day 70	2449	970
B6	snRNA-seq	BATCH-2_D70_CNT, BATCH-2_D70_control1, BATCH-2_D70_control2, BATCH-2_D70_control3, BATCH-2_D70_FGF1, BATCH-2_D70_organoid	day 70	2461	1184
B7	snRNA-seq	BATCH-3_D70_CNT, BATCH-3_D70_control1, BATCH-3_D70_control2, BATCH-3_D70_control3, BATCH-3_D70_FGF1, BATCH-3_D70_organoid	day 70	3330	231
B8	snRNA-seq	BATCH-3_D70_CNT, BATCH-3_D70_control1, BATCH-3_D70_control2, BATCH-3_D70_control3, BATCH-3_D70_FGF1	day 70	2277	192
B10	snRNA-seq	BATCH-3_D70_control1, BATCH-3_D70_control2, BATCH-3_D70_control3, BATCH-3_D70_FGF1	day 70	995	89

Supplementary table 5. Overview of scRNAseq and snRNAseq 10x experiments with RC17 as cell line and BMP4 5-14 as BMP timing, related to STAR methods**.**

Sample	Approach	Bmp timing	Cell line	Day	Cells before filtering	Cells after filtering
AM30E_d16	snRNA-seq	BMP4 5-7	RC17	day 16	4885	3755
AM30C_d16	snRNA-seq	BMP4 5-11	RC17	day 16	4579	3499
AM34E_d16	snRNA-seq	BMP4 5-7	KOLF	day 16	3495	2909
AM30D_d16	snRNA-seq	BMP4 5-9	RC17	day 16	3504	2781
AM34D_d16	snRNA-seq	BMP4 5-9	KOLF	day 16	3294	2713
AM30B_d16	snRNA-seq	BMP4 5-14	RC17	day 16	3343	2678
AM34C_d16	snRNA-seq	BMP4 5-11	KOLF	day 16	2936	2403
AM34K_d16	snRNA-seq	BMP4 5-7	BIOi010	day 16	2864	2317
AM34H_d16	snRNA-seq	BMP4 5-11	BIOi010	day 16	2817	2290
AM34F_d16	snRNA-seq	no BMP4	BIOi010	day 16	2814	2189
AM34B_d16	snRNA-seq	BMP4 5-14	KOLF	day 16	2639	2167
AM34I_d16	snRNA-seq	BMP4 5-9	BIOi010	day 16	2623	2138
AM34G_d16	snRNA-seq	BMP4 5-14	BIOi010	day 16	2477	2015
AM34A_d16	snRNA-seq	no BMP4	KOLF	day 16	1951	1614
AM30A_d16	snRNA-seq	no BMP4	RC17	day 16	1021	804
AM34D_d60	snRNA-seq	BMP4 5-9	KOLF	day 50	5053	3747
XAM11_1_B_d50	snRNA-seq	no BMP4	RC17	day 50	4877	3744
AM34K_d50	snRNA-seq	BMP4 5-7	BIOi010	day 50	5055	3681
AM34B_d50	snRNA-seq	BMP4 5-14	KOLF	day 50	4529	3311
XAM11_2_A_d50	snRNA-seq	no BMP4 or 7	KOLF	day 50	4406	3240
AM34G_d50	snRNA-seq	BMP4 5-14	BIOi010	day 50	3762	2986
AM34I_d50	snRNA-seq	BMP4 5-9	BIOi010	day 50	3978	2979
AM34E_d60	snRNA-seq	BMP4 5-7	KOLF	day 50	3745	2912
XAM11_2_B_d50	snRNA-seq	no BMP4 or 7	RC17	day 50	3951	2910
XAM11_2_C_d50	snRNA-seq	no BMP4 or 7	BIOi010	day 50	3863	2892
AM30D_d50	snRNA-seq	BMP4 5-9	RC17	day 50	3559	2689
AM30C_d50	snRNA-seq	BMP4 5-11	RC17	day 50	5357	2683

AM34H_d50	snRNA-seq	BMP4 5-11	BIOi010	day 50	3547	2660
AM30E_d50	snRNA-seq	BMP4 5-7	RC17	day 50	3242	2491
AM30B_d80	snRNA-seq	BMP4 5-14	RC17	day 80	3049	2319
AM39_d110	snRNA-seq	BMP4 5-14	RC17	day 110	2742	2253
XAM11_1_A_d50	snRNA-seq	no BMP4	KOLF	day 50	2687	2098
AM34C_d50	snRNA-seq	BMP4 5-11	KOLF	day 50	2572	1908
AM30B_d50	snRNA-seq	BMP4 5-14	RC17	day 50	2046	1174
XAM11_1_C_d50	snRNA-seq	no BMP4	BIOi010	day 50	706	529

Supplementary table 6. Overview of snRNAseq Parse Biosciences experiments, related to STAR methods**.**

Supplemental references

1. Herb, B.R., Glover, H.J., Bhaduri, A., Colantuoni, C., Bale, T.L., Siletti, K., Hodge, R., Lein, E., Kriegstein, A.R., Doege, C.A., et al. (2023). Single-cell genomics reveals region-specific developmental trajectories underlying neuronal diversity in the human hypothalamus. Sci. Adv. *9*, eadf6251. https://doi.org/10.1126/sciadv.adf6251.
2. Tadross, J.A., Steuernagel, L., Dowsett, G.K.C., Kentistou, K.A., Lundh, S., Porniece, M., Klemm, P., Rainbow, K., Hvid, H., Kania, K., et al. (2025). A comprehensive spatio-cellular map of the human hypothalamus. Nature *639*, 708–716. https://doi.org/10.1038/s41586-024-08504-8.
3. Braun, E., Danan-Gotthold, M., Borm, L.E., Lee, K.W., Vinsland, E., Lönnerberg, P., Hu, L., Li, X., He, X., Andrusivová, Ž., et al. (2023). Comprehensive cell atlas of the first-trimester developing human brain. Science (1979). *382*. https://doi.org/10.1126/science.adf1226.
