# Supplementary material for: Generation of human appetite-regulating neurons and tanycytes from pluripotent stem cells
Source: Cell Stem Cell. 2026 Jul 2;33(7):1174–1190.e11. doi: 10.1016/j.stem.2026.05.005 (PMC13353052; doi:10.1016/j.stem.2026.05.005)
Supplement: Document S2. Article plus supplemental information [file mmc3.pdf]

Cell Stem Cell

Article

# Generation of human appetite-regulating neurons and tanycytes from pluripotent stem cells

## Graphical abstract

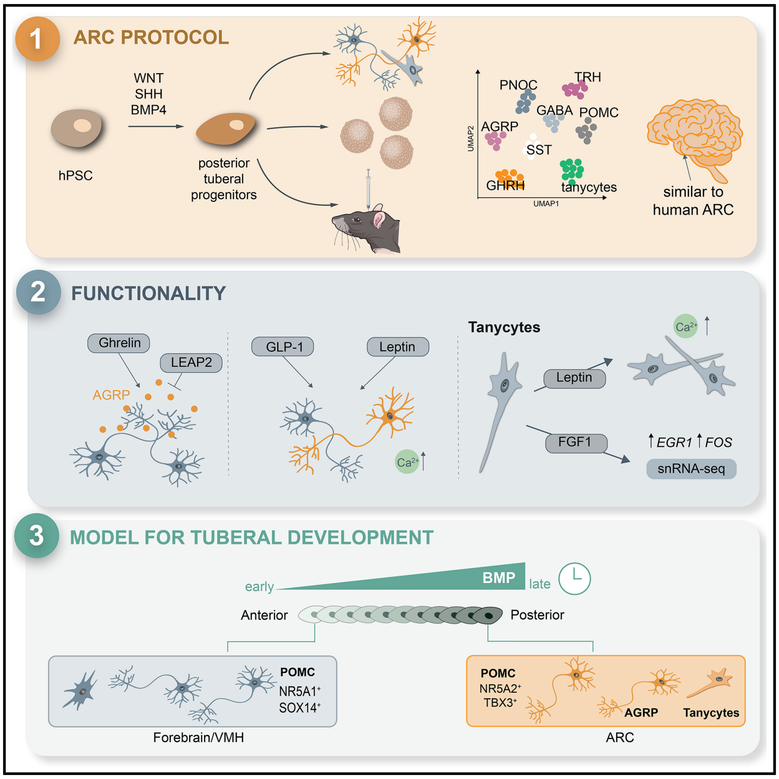


## Highlights

- ARC-specific cell types from NKX2.1$^+$/RAX$^+$/FGF10$^+$/TBX3$^+$ posterior tuberal progenitors
- ARC neurons and tanycytes functionally respond to leptin, ghrelin, LEAP2, FGF1, and GLP-1
- ARC versus VMH fate is controlled by a temporal wave of BMP
- POMC neurons exist in two subtypes—one of which is not associated to the ARC


## Authors

Zehra Abay-Nørgaard,
Anika K. Mueller, Erno Hänninen, ...,
Janko Kajtez, Tune H. Pers,
Agnete Kirkeby

## Correspondence

agnete.kirkeby@sund.ku.dk



## In brief

Abay-Nørgaard, Mueller, Hänninen, et al. generate cultures of the hypothalamic arcuate nucleus from hPSCs. They show transcriptional similarity to human ARC and response to appetite-regulating cues. BMP duration regulates ARC versus neighboring VMH fate and reveals the identification of divergent *in vitro* satiety-promoting POMC neurons of arcuate and non-arcuate origin.


Check for updates

Abay-Nørgaard et al., 2026, Cell Stem Cell *33*, 1174–1190
July 2, 2026 © 2026 The Author(s). Published by Elsevier Inc.
https://doi.org/10.1016/j.stem.2026.05.005

CellPress

CellPress
OPEN ACCESS

Cell Stem Cell

Article

# Generation of human appetite-regulating neurons and tanycytes from pluripotent stem cells

Zehra Abay-Nørgaard,[1,5] Anika K. Mueller,[1,5] Erno Hänninen,[1,5] Dylan Rausch,[2] Louise Piilgaard,[1] Lucía Sena Trujillo,[1] Lorenzo Fedrizzi,[1] Jens Bager Christensen,[1] Alison Salvador,[1] Alrik L. Schörling,[1] Noah Wulff Mottelson,[2] Qiuyu Qin,[1] Shruthi Sampath,[1] Bob Hersbach,[1] Jonas Henkenjohann,[1] Sofie Peeters,[1] Viktoriia Nikulina,[3] Charlotte Høy Kruse,[2] Yuan Li,[3] Kavitha Chinnaiya,[4] Marysia Placzek,[4] Janko Kajtez,[1] Tune H. Pers,[2] and Agnete Kirkeby[1,6,*]

[1]Novo Nordisk Foundation Center for Stem Cell Medicine (reNEW), Department of Biomedical Sciences, Faculty of Health and Medical Sciences, University of Copenhagen, 2200 Copenhagen, Denmark

[2]Novo Nordisk Foundation Center for Basic Metabolic Research (CBMR), Faculty of Health and Medical Sciences, University of Copenhagen, 2200 Copenhagen, Denmark

[3]Department of Experimental Medical Sciences, Wallenberg Centre for Molecular Medicine (WCMM) and Lund Stem Cell Centre, Lund University, 221 84 Lund, Sweden

[4]School of Biosciences, University of Sheffield, Sheffield S10 2TN, UK

[5]These authors contributed equally

[6]Lead contact

*Correspondence: agnete.kirkeby@sund.ku.dk

https://doi.org/10.1016/j.stem.2026.05.005

## SUMMARY

The arcuate nucleus (ARC) and ventromedial hypothalamus (VMH) are highly specialized hypothalamic nuclei controlling appetite and energy expenditure. Here, we demonstrate that human VMH and ARC neurons can be generated from pluripotent stem cells by fine-tuned timing and duration of bone morphogenetic protein (BMP) exposure. We identified $SHH^{-}$/$NKX2.1^{+}$/$FGF10^{+}$/$RAX^{+}$/$TBX3^{+}$ posterior tuberal progenitors as the source of ARC cell types, including agouti-related peptide (AGRP)-, prepronociceptin (PNOC)-, growth-hormone-releasing hormone (GHRH)-, and thyrotropin-releasing hormone (TRH)-expressing neurons and β2-tanycytes. Differentiated ARC cultures showed high transcriptomic similarity to human ARC and responded to energy homeostasis-regulatory peptides including leptin, glucagon-like peptide 1 (GLP-1), ghrelin, and fibroblast growth factor 1 (FGF1). In contrast, anterior tuberal $TBX3^{-}$ progenitors generated VMH-associated neurons expressing NR5A1, *SOX14*, and *GPR149*. Strikingly, two transcriptionally distinct pro-opiomelanocortin (POMC) subpopulations emerged from these lineages, mapping spatially to either the ARC ($POMC^{+}$/$TBX3^{+}$/$NR5A2^{+}$) or the VMH ($POMC^{+}$/$SOX14^{+}$/$NR5A1^{+}$). This model provides a cellular platform to study human hypothalamic subtype specification and pathways involved in central appetite regulation.

## INTRODUCTION

Food intake and energy expenditure are predominantly governed by hypothalamic circuits, particularly by first-order arcuate nucleus (ARC) neurons, agouti-related peptide (AGRP), and pro-opiomelanocortin (POMC). These neurons integrate and relay information on the body's fed state to second-order neurons within the hypothalamus and other brain regions.[1–3] The neighboring ventromedial hypothalamus (VMH) similarly functions as a satiety center by sensing glucose levels and suppressing food intake.[4,5] In addition, specialized radial glial-like cells lining the third ventricle known as tanycytes play a key role in transporting hormones into the hypothalamus.[6–8] Consequently, impairment of hypothalamic populations is associated with metabolic diseases such as obesity and type 2 diabetes (T2D).[8,9] Recent blockbuster obesity drugs acting through the glucagon-like peptide 1 receptor (GLP1R, i.e., semaglutide and tirzepatide)[10] are hypothesized to influence feeding behavior through hypothalamic circuits.[11–14] However, despite valuable animal models, the direct molecular actions of glucagon-like peptide 1 (GLP-1) and other appetite-regulating peptides on individual cell types of the hypothalamus are still unclear.[9,15] *In vitro* models that recapitulate specialized human hypothalamic cell types can serve as a powerful tool for cellular and molecular pathway investigation. However, the generation of specific hypothalamic populations from human pluripotent stem cells (hPSCs) remains a challenge due to the high anatomical and cellular complexity of the hypothalamus.[16–20] Furthermore, the lineage trajectories and anatomical relationships between hypothalamic progenitors and their corresponding adult subtypes remain highly debated, and this has been further fueled by a recent study showing that topological relationships become distorted through anisotropic growth during early hypothalamic development.[21–25] Since 2015, several studies have successfully

 © 2026 The Author(s). Published by Elsevier Inc.
This is an open access article under the CC BY license (http://creativecommons.org/licenses/by/4.0/).

generated hPSC-derived hypothalamic cultures, some with high yields of appetite-suppressing POMC neurons.[26–30] Yet, these protocols often fail to generate other subtypes associated with the ARC and VMH, such as AGRP, prepronociceptin (PNOC), growth-hormone-releasing hormone (GHRH) neurons, and tanycytes,[15,26–28,31–33] and the reasons for this discrepancy remain unclear.

A recent study in the developing chick embryo revealed that a temporal wave of bone morphogenetic protein (BMP) signaling directs the early specification of tuberal hypothalamic progenitors into anterior and posterior fates.[34,35] Given that the tuberal hypothalamus gives rise to the ARC, the VMH, and the dorsomedial hypothalamic nucleus (DMH), we hypothesized that temporally confined BMP signaling may also play an important role in the sub-regionalization of these nuclei in the human hypothalamus.

Here, we show that the temporal BMP wave identified in the chick can be recreated *in vitro* and that it is a key player in the regional patterning of human tuberal hypothalamic nuclei and their associated neuronal populations. Analogous to the events identified in the chick embryo,[34] the timing of BMP was crucial for derivation of posterior versus anterior tuberal progenitors in a human stem cell model. Upon maturation, the posterior progenitors gave rise to ARC-related POMC, PNOC, thyrotropin-releasing hormone (TRH), and AGRP neurons as well as tanycytes, whereas anterior progenitors gave rise to VMH-related NR5A1 and POMC neurons. Distinct POMC transcriptional subtypes were derived from anterior versus posterior progenitor populations, providing important insights into hypothalamic subtype development. The functionality of the ARC cultures was validated through AGRP secretion and functional responsiveness to fibroblast growth factor 1 (FGF1), leptin, GLP-1, and ghrelin. In summary, these human *in vitro* models of the ARC and VMH reveal insights into human tuberal hypothalamic development and enable a platform for the molecular dissection of appetite control.

## RESULTS

### Signaling factors required for generation of human ARC *in vitro*

The secreted signals WNT, BMP, and Sonic hedgehog (SHH) guide regionalization of the neural tube, both *in vivo*[36] and *in vitro*[37] (Figure 1A, top). To generate human neurons of the tuberal hypothalamus, we subjected a human embryonic stem cell (hESC) line (RC17) to a multitude of neural differentiation paradigms with varied timing and concentrations of molecules targeting key neurodevelopmental pathways (Figure 1A, bottom).[30] Each hPSC differentiation ($n = 113$) was analyzed by quantitative reverse transcription PCR (RT-qPCR) using a primer panel of 64 genes that mark hypothalamic nuclei and surrounding regions (Figure S1A). To identify combinations of factors that produced distinct hypothalamic fates, we performed a principal-component (PC) analysis, grouping the differentiation outcomes based on their gene expression profiles. PC1 appeared to separate samples based on the expression of canonical ARC (*POMC*, *RAX*, *FGF10*, and *TBX3*) versus paraventricular nucleus (PVN; *BRN2*, *SIM1*, and *SIM2*) genes (Figure 1B). We investigated which differentiation conditions drove regionalization toward ARC and PVN fates (i.e., along the PC1 axis) using a covariant analysis (Figure 1C). This showed that early addition of CHIR99021 (a canonical WNT activator) at day 0 and late addition of SHH at day 6 favored PVN fates, while addition of CHIR from day 2 favored ARC specification, but only when combined with addition of BMP4 (Figures 1C and S1B). While all conditions expressed the ventral forebrain marker *NKX2-1*, only the predicted PVN patterning condition induced expression of the PVN marker *SIM1*, whereas predicted ARC conditions upregulated the posterior tuberal markers *RAX* and *TBX3* in day 16 progenitor cultures (Figures 1D and S1B). Posterior tuberal identity and AGRP neuron generation were dependent on BMP4 addition, here added from days 5 to 14, whereas POMC neurons could be generated both with and without BMP4 (Figure 1D, bottom). BMP7, in addition to BMP4, has been implicated in hypothalamic development in animal models.[34,35,38] We found no significant difference in the key ARC markers in BMP4- versus BMP7-treated cultures (Figures S1C–S1E), indicating that they can act redundantly in ARC fate induction. Mining of a transcriptomic dataset of human fetal hypothalamus[39] showed that whereas *BMP4* was mainly expressed in transitioning radial glial cells, *BMP7* was expressed in both progenitors and postmitotic *TBX3*$^+$ ARC neuronal precursors (Figures S1F–S1H). In line with this, BMP7 during the maturation phase (days 25–50) induced both *TBX3* and *POMC* at day 50 (Figure S1I), and substitution with BMP4 generated comparable results (Figures S1J and S1K). ARC precursors in the fetal hypothalamus were also found to express the developmentally secreted protein *IGFBP3* (insulin-like growth-factor-binding protein 3), presenting this as another potential specification factor for our *in vitro* differentiation protocol (Figures S1F–S1H). Indeed, IGFBP3 from days 6 to 14 of differentiation enriched for POMC neurons (Figure S1L). Early-stage BMP4, IGFBP3, and late-stage BMP7 were therefore included in the ARC differentiation protocol (Figure 1E).

To test the differentiation potential of ARC progenitors, we matured the progenitors *in vitro* in 2D and 3D (Figure 1E). Immunocytochemistry (ICC) at day 50 revealed AGRP$^+$, POMC$^+$, TH$^+$, TRH$^+$, Neuropeptide Y (NPY)$^+$, and GHRH$^+$ neurons (Figures 1F and 1G). As expected from mouse data, the AGRP neurons co-expressed $\gamma$-aminobutyric acid (GABA) and somatostatin (SST)[40] and a subset of AGRP$^+$ cells co-expressed the hyperphagia-inducing *PNOC* gene.[41,42] POMC neurons expressed the appetite-regulating neuropeptides $\alpha$-melanocyte-stimulating hormone ($\alpha$-MSH) and $\beta$-MSH (Figure 1H)—both are derived from the POMC transcript, the latter only exists in humans.[43,44] Quantification of AGRP and POMC neurons in ARC spheroids from three different hPSC lines (RC17 hESC, KOLF2.1 hiPSC, and BIONi010 hiPSC) revealed an average of 4.53% ± 0.69% (mean ± SEM) AGRP$^+$ neurons and 5.90% ± 1.19% $\alpha$-MSH$^+$ neurons relative to DAPI$^+$ nuclei (Figure 1I). Other protocols have reported POMC$^+$ proportions ranging from 0.1% to ~50%,[26,28] and AGRP+ proportions from 0.1% to 0.2%.[28,45]

To validate the lineage commitment of the ARC progenitors, we further performed xenotransplantation into the striatum of adult immunodeficient rats, allowing maturation in an ectopic *in vivo* environment without exogenous growth factors (Figure 1J). The absence of hypothalamic peptidergic neurons in the striatum allowed us to unambiguously identify human-derived ARC

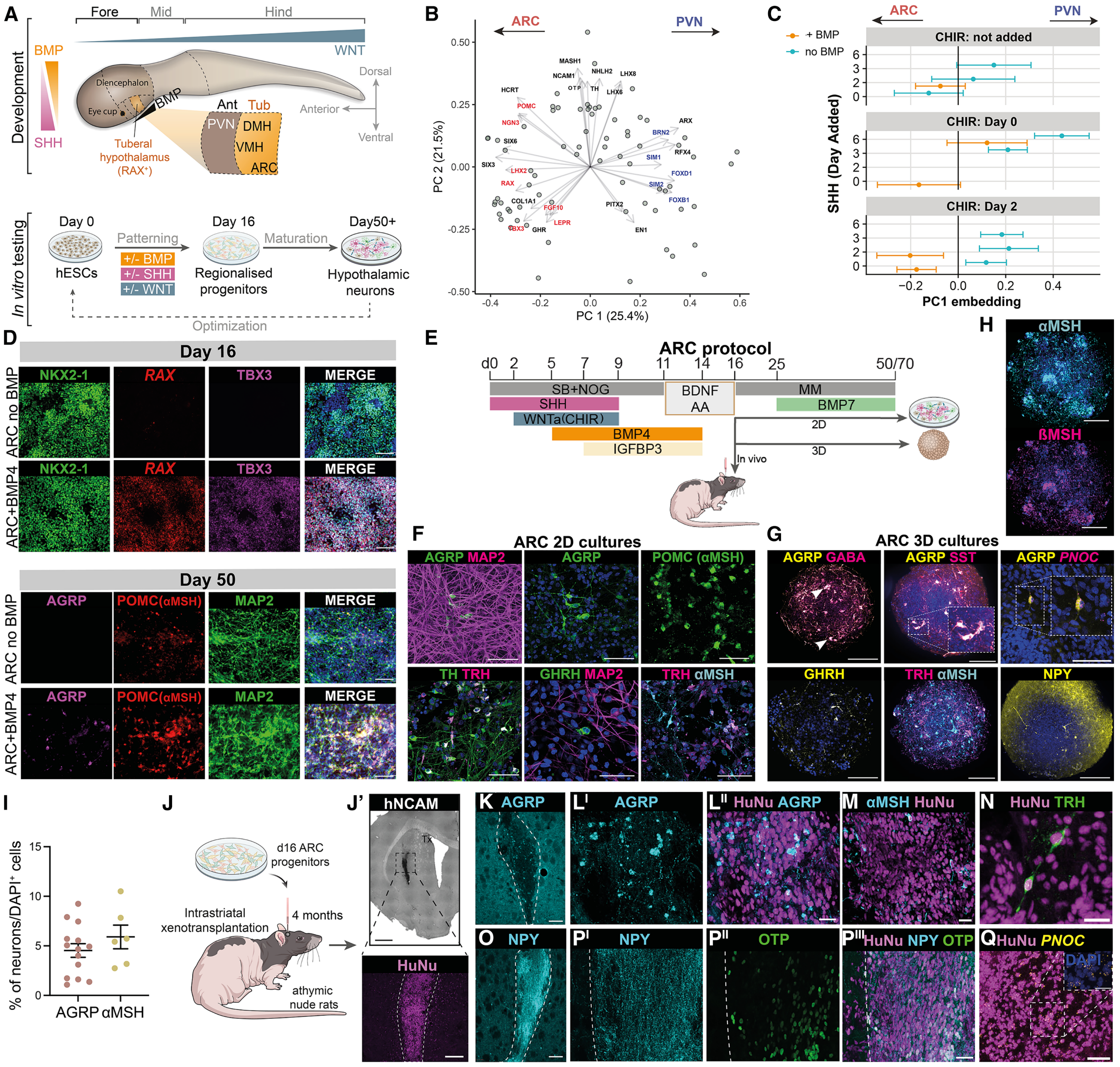


**Figure 1. Signaling factors required for generation of human ARC *in vitro***

(A) Hypothalamic development *in vivo* and *in vitro* differentiation of hPSC into hypothalamic cells.

(B) Principal-component analysis (PCA) of day 16 RT-qPCR data (RC17), clustering according to ARC- and PVN-associated genes. Each dot represents one differentiation batch (*n* = 113).

(C) Estimated marginal means assessing the effect of factors on PC1, adjusted for covariates.

(D) ICC/ISH at days 16 and 50 for ARC protocol with or without BMP4. Scale bars: 100 μm.

(E) ARC differentiation protocol and maturation options.

(F–H) ICC of day 50 in 2D (F) and 3D (G and H), scale bars: 50 and 100 μm.

(I) Quantification of day 50 3D spheroids (AGRP: *n* = 14, 4.53% ± 0.69%, mean ± SEM; αMSH: *n* = 6, 5.90% ± 1.19%).

(J) Xenotransplantation of day 16 ARC progenitors into nude rats.

(J′) Top: ICC for human neural cell adhesion molecule (NCAM) highlights graft site, scale bar: 2 mm. Bottom: IF for human nuclear antigen (HuNu), scale bar: 200 μm.

(K–Q) IF for ARC neuronal markers in grafts. Scale bars: 100 μm (K and O), 50 μm (N), 25 μm (L, M, P, and Q).

subtypes in the xenografts using immunofluorescence (IF) and *in situ* hybridization (ISH). At 4 months post-transplantation, we confirmed that the progenitors survived (Figure S2A) and matured into AGRP-, POMC-, TRH-, OTP-, and *PNOC*-expressing neurons, thereby validating our observations *in vitro* (Figures 1K–1Q and S2B). We also observed strong NPY fiber staining in the

CellPress
OPEN ACCESS

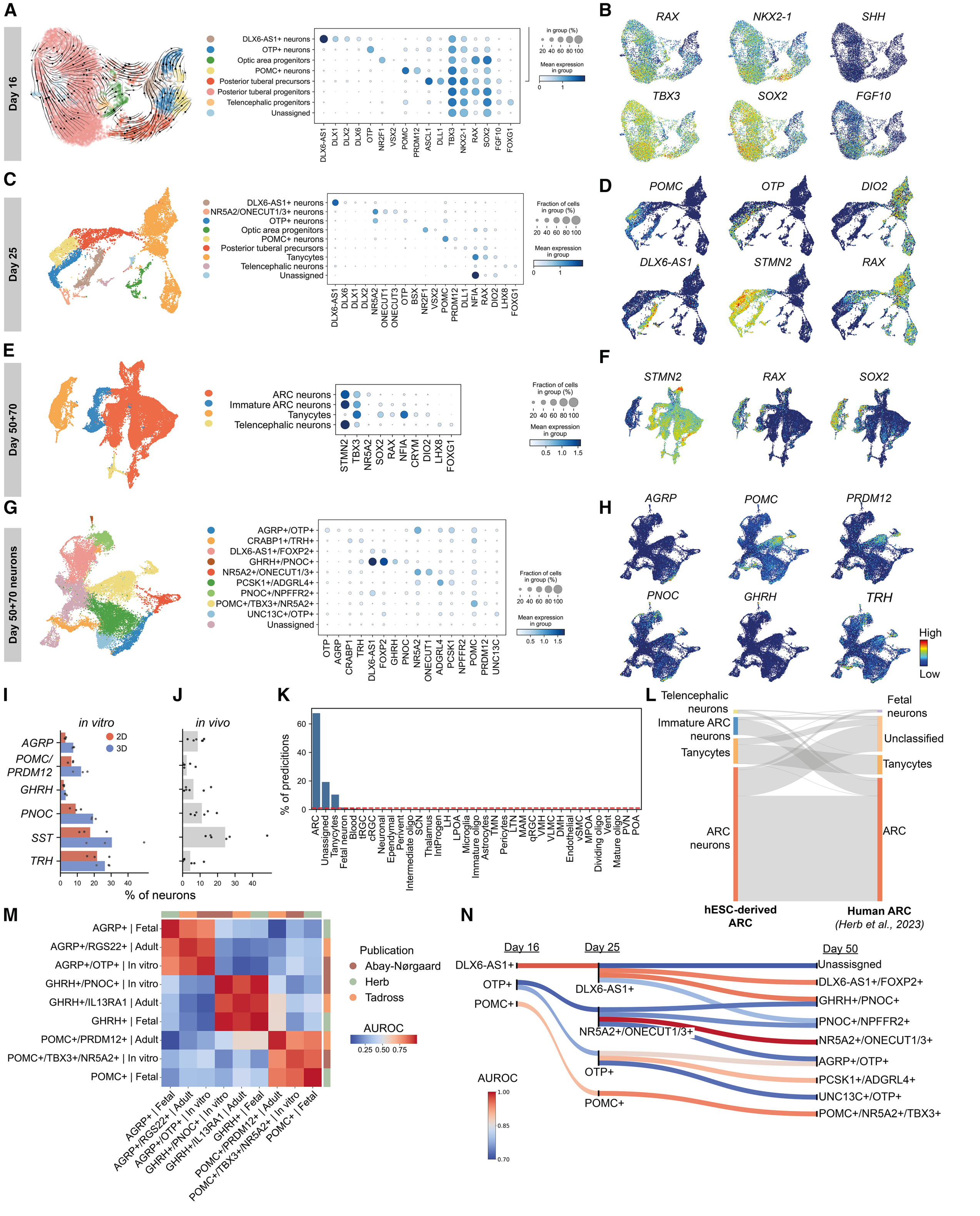


**Figure 2. *In vitro*-derived ARC cultures show transcriptional similarity to the human ARC**

(A–H) Uniform manifold approximation and projections (UMAPs) of sc/snRNA-seq data with annotations, dot plot of marker genes and feature plots of representative genes at day 16 (A and B), day 25 (C and D), days 50 + 70 (E and F) and days 50 + 70 neuronal clusters (G and H) (*n* = 3; RC17).

(I) % cells expressing neuropeptide transcripts in days 50 + 70 neurons. Each dot = biological replicate (*n* = 3).

(legend continued on next page)

grafts, closely resembling the diffuse innervation found from OTP+/NPY+ neurons in the endogenous rat ARC and PVN, and morphologically distinct from the OTP−/NPY+ interneuron cell bodies found in the cortex and striatum (Figures 1O, 1P, and S2C–S2E). These results confirmed that a combination of the developmental signaling factors WNT, SHH, BMP4/7, and IGFBP3 could drive hPSC toward a committed ARC fate by day 16, and these progenitors showed capacity to produce ARC-related neuron upon *in vitro* and *in vivo* maturation.

## *In vitro*-derived ARC cultures show transcriptional similarity to the human ARC

To characterize the composition of our ARC cultures, we performed single-cell and single-nucleus RNA sequencing (sc/snRNA-seq) of three biological replicates of RC17-derived ARC cells at days 16, 25, 50, and 70 (Figures 2A–2H; Table S7). Early-stage day 16 cultures were primarily composed of posterior tuberal progenitors and precursors (79.8 ± 2.0%, mean ± SD) and some *STMN2*+ hypothalamic neurons (13.6 ± 1.4%) (Figures 2A, 2B, and S3A–S3C). The *STMN2*+ fraction contained populations expressing *POMC*, *OTP*, and *DLX6-AS1* (Figures 2A and S3B), indicative of early ARC lineage specification as previously described in the mouse.[23] In day 25 dataset, we identified four neuronal clusters—OTP+, DLX6-AS1+, POMC+, and NR5A2+/ONECUT1/3+ cluster—as well as an NFIA+/DIO2+/RAX+ tanycyte cluster (Figures 2C, 2D, and S3C–S3E). After 50–70 days in culture, the majority of cells consisted of ARC neurons (72.4 ± 4.2%, mean ± SD) and tanycytes (14.7 ±1.1 %) (Figures 2E, 2F, and S3F–S3H).

Next, we subsetted all neurons from the days 50 + 70 dataset and annotated them into ten ARC-associated clusters (Figures 2G, 2H, and S3I–S3L). Consistent with our ICC data showing expression of AGRP and α-MSH (Figures 1F and 1G), we identified AGRP+/OTP+ and POMC+/TBX3+/NR5A2+ clusters. *PNOC* was expressed in the AGRP+/OTP+ cluster (Figure 2H), as well as two other clusters co-expressing either *GHRH*+ or *NPFFR2*+, corresponding to populations previously identified in the mouse.[33,46] PNOC neurons have been shown to be leptin sensitive and regulate feeding behavior in mice.[41,42,47] Indeed, 15.3 ± 1.5% (mean ± SD) of our GHRH+/PNOC+ and 12.6 ± 5.6% of PNOC+/NPFFR2+ neurons co-expressed *PNOC* and leptin receptor (*LEPR*), comparable to the 20% *Lepr*+ PNOC neurons reported in the mouse[41] (Figure S3M). Within the DLX6-AS1+/FOXP2+ cluster, we identified a population of *BNC2*+/*LEPR*+ neurons (Figure S3L), which have been identified in the mouse ARC as acute suppressors of food intake.[48] The ARC cultures also contained a CRABP1+/TRH+ cluster that co-expressed *GAD2* and *LEPR*, possibly corresponding to the recently described *CRABP1*+/*LEPR*+ GABAergic neurons that promote feeding in mice.[49] While these cells have been reported in the mouse,[17] they have not yet been characterized in human. Most neuronal subtypes were found in both 2D and 3D conditions; however, 3D notably enhanced the generation of *AGRP*+ (7.6% ± 0.4%, mean ± SD), *POMC*+/*PRDM12*+ (12.2% ± 3.6%), and *GHRH*+ neurons (3.1% ± 0.5%) (Figure 2I). Comparison of our data with a hypothalamic snRNA-seq dataset showed that the proportions of neuropeptidergic populations in the hPSC-derived ARC cultures were similar to the adult human ARC,[20] with the exception of *TRH*+ cells, which were higher *in vitro* compared with *in vivo* (21.6% ± 5.5% in 2D, 26.3% ± 3.5% in 3D versus 4.3% ± 4.3% *in vivo*, mean ± SD) (Figure 2J). The proportions of cell types were highly reproducible in three biological replicates of RC17-derived ARC across all sequenced time points, highlighting the robustness of the protocol (Figure S3C).

We further assessed the similarity of our *in vitro*-derived cells to human fetal and adult hypothalamic tissue through sc/snRNA-seq datasets.[20,39] When mapping our data to a fetal human hypothalamus reference from the first and second trimester,[39] our cells specifically mapped to the human ARC (67% ± 3.7%, mean ± SD) and tanycyte (10% ± 0.6%) populations (Figure 2K) over all other hypothalamic populations. Fewer than 1.4% of the hPSC-derived ARC cells mapped to other hypothalamic nuclei or unrelated non-neuronal cell types in the human dataset. Notably, the *in vitro* tanycytes mapped almost exclusively to "tanycytes" in the human reference, and not to other non-neuronal cells including astrocytes, pericytes, ependymal cells, and endothelial cells (Figure 2L). When comparing our *in vitro*-derived AGRP, POMC, and GHRH neurons to their fetal and adult human counterparts[20,39] through MetaNeighbor analysis,[50] they showed highest transcriptional similarity to their respective *in vivo* subtype counterparts (Figure 2M). RNA velocity of day 16 progenitors indicated the posterior tuberal progenitors as the precursors of neuronal ARC lineages (Figure 2A). MetaNeighbor-based cluster similarity analysis of all postmitotic ARC neuronal clusters from days 16, 25, and 50 + 70 indicated that lineages leading to mature AGRP, POMC, and GHRH neurons were already specified in the postmitotic cluster emerging at day 16 (Figure 2N). POMC neurons only showed similarity with *POMC*+ progenitors from earlier time points. Likewise, the analysis indicated lineage trajectories from *OTP*+ day 16 progenitors to *NR5A2*+ and *AGRP*+ cells as well as from *DLX* progenitors to GHRH+/PNOC+ cells, which is in line with findings in the developing mouse.[51,52]

## *In vitro*-derived tanycytes map to the β2 subtype and respond to FGF1

Tanycytes are specialized glucose-sensing glial cells around the third ventricle of the hypothalamus.[8] scRNA-seq of the mouse have classified tanycytes into α1 and α2 subtypes located dorsally along the third ventricle and β1 and β2 subtypes located ventrally in the median eminence.[40] To characterize the subtype identity and developmental trajectories of the tanycytic lineage, we integrated all cells from the day 16 scRNA-seq dataset with the tanycyte-annotated cluster from days 25 and 50 + 70 (Figures 3A and 3B). Pseudotime analysis showed bidirectional

(J) % of cells expressing neuropeptide transcripts in the adult human ARC.[20] Each dot = donor (*n* = 6).
(K) % predicted cell types from days 50 + 70 dataset to human fetal hypothalamus.[39] Red dashed line marks 1% threshold.
(L) Sankey diagram between ARC culture and reference,[39] including only clusters representing >1% of predictions from (K).
(M) MetaNeighbor cluster similarity scores between ARC subtypes and subtypes in human fetal and adult datasets.[20,39]
(N) Sankey plot of subtype cluster similarities across days 16, 25, and 50 + 70.

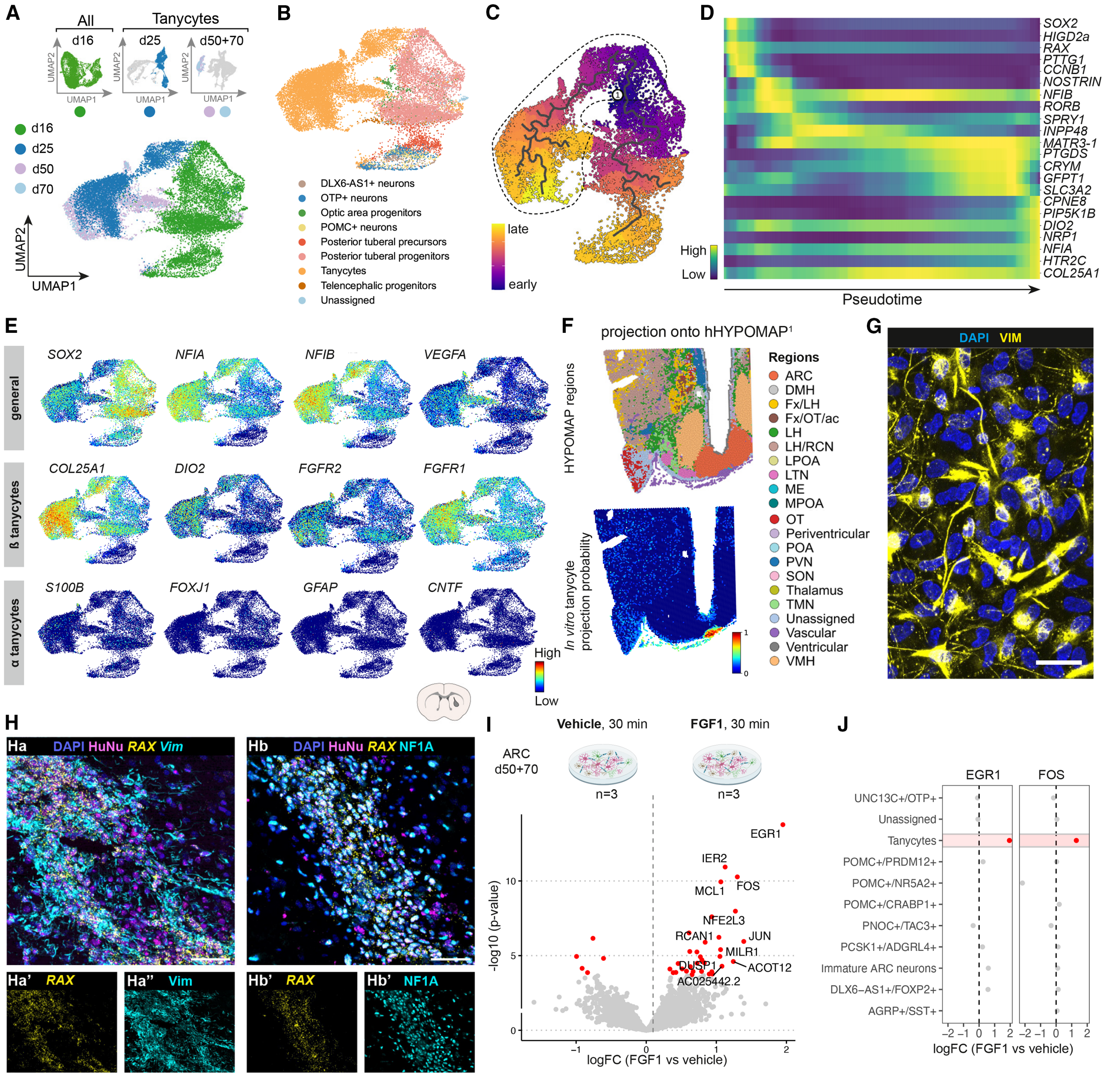

**Figure 3. *In vitro*-derived tanycytes map to the b2 subtype and respond to FGF1**

(A–C) (A) Integration of day 16 data with "tanycytes" cluster from days 25 and 50 + 70 datasets, (B) annotated clusters, and (C) pseudotime trajectory (monocle 3), (1) indicates trajectory root.

(D) Gene expression dynamics of selected markers of dotted outline from (C) along pseudotime.

(E) Feature plots of key tanycyte markers.

(F) Mapping spatial location of the days 50 + 70 tanycyte cluster onto spatial transcriptomic reference of human hypothalamus.[20]

(G) ICC on 2D ARC, scale bar: 25 μm.

(H) ICC/ISH on xenografts, scale bars: 50 μm.

(I) Volcano plot of DEG in tanycytes after 30min stimulation of 50 ng/mL FGF1 in days 50 + 70 cultures (*n* = 3 each, RC17). Benjamini-Hochberg corrected *p* values.

(J) DE of *EGR1* and *FOS* across annotated cell types. Significant upregulation in tanycytes shown in red.

maturation trajectories from tuberal progenitors toward either neurons or tanycytes (Figure 3C). According to the pseudotime trajectory, the RAX$^+$/SOX2$^+$ progenitors transitioned toward a mature identity that expressed *CRYM* and the pan-glial marker *NFIA* (Figure 3D). The mature cluster also expressed the tanycytic marker *VEGFA*, which plays an important role in regulating capillary fenestration in the median eminence[53] (Figure 3E). While the tanycytes highly expressed β-tanycyte markers (*COL25A1* and *FGFR1*[54,55]), α-tanycyte markers were absent or only lowly expressed (Figure 3E). Mapping to a spatial

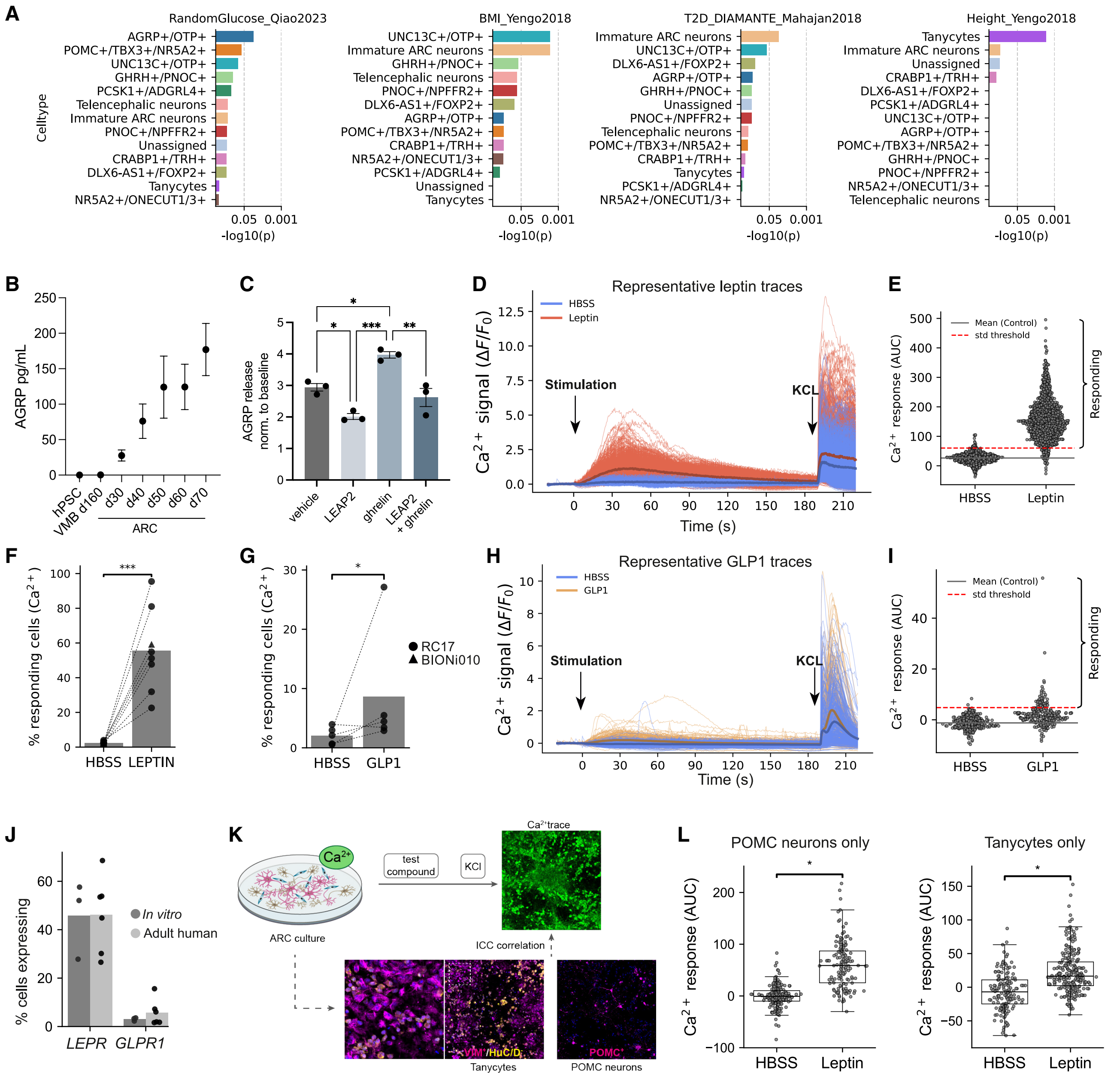


**Figure 4. *In vitro*-derived ARC neurons show responsiveness to leptin and GLP-1**

(A) CELLECT[58] of days 50 + 70 clusters (Figure 2E) with GWAS traits, including BMI,[61] T2D,[62] and random glucose,[59] height as a negative control.[61] Bonferroni-adjusted CELLECT *p* value.

(B) AGRP ELISA of ARC culture medium ($n = 3$, RC17) compared with hPSC and ventral midbrain organoid (VMB); mean ± SEM.

(C) AGRP ELISA of culture medium of stimulated ARC at day 143 with 100 nM ghrelin and/or 2 μM LEAP2 ($n = 2$ BIONi010, $n = 1$ SCTi003-A). Mean ± SEM. One-way ANOVA with Tukey's test: vehicle (V) versus ghrelin $p = 0.0118$; V versus LEAP2 $p = 0.0209$; ghrelin versus LEAP2 $p = 0.0002$; ghrelin versus LEAP2 + ghrelin $p = 0.0023$.

(D) Representative calcium imaging trace of ARC days 50–71 (RC17) upon addition of 100 ng/mL leptin or Hank's balanced salt solution (HBSS) as control (0 s) and KCl (190 s). Thin lines represent single cells, thick lines represent average signal of all annotated cells.

(E) Calcium area under the curve (AUC) values for leptin including mean (gray line) and response threshold (two SD, red dashed line). Each dot = single cell.

(F and G) % of days 50–71 cells responsive for leptin (F; $n = 8$, RC17 hESC and BIONi010 hiPSC) and GLP-1 (G; $n = 5$, RC17). Paired sample *t* test ($p = 0.0004$) in (F) and paired sample *t* test on log-transformed data $p = 0.046$ in (G).

(H) Representative calcium trace of ARC days 50–71 (RC17) upon addition of 100 nM GLP-1 or HBSS.

(I) Calcium AUC values for GLP-1.

(legend continued on next page)

transcriptomic atlas of the adult human hypothalamus[20] confirmed the *in vitro*-derived tanycytes were most similar to β2-tanycytes (Figures 3F and S4A–S4D). Tanycytes showed elongated morphology *in vitro* (Figure 3G) and were also found to be present in 4-month-old xenografts, confirmed by *RAX*, VIM, and NFIA expression (Figure 3H). To functionally analyze our *in vitro*-derived tanycytes, we investigated responses to FGF1, a growth factor known to induce robust transcriptional changes in tanycytes and astrocytes to mediate sustained remission of diabetic hyperglycemia following injection into the rodent brain.[56,57] We treated ARC cultures with FGF1 for 30 min following snRNA-seq analysis (Figures 3I and 3J). The treated cultures showed an FGF1 response exclusively in the tanycyte population as evidenced by strong induction of early response genes *FOS*, *IER2*, and *EGR1* compared with the vehicle control. This aligned with the expression of *FGFR2* in the tanycytes (Figure 3E). In summary, we concluded that the ARC protocol produced functionally responsive human β2-like tanycytes derived from posterior tuberal progenitors.

## *In vitro*-derived ARC neurons show responsiveness to ghrelin, leptin, and GLP-1

To address whether the cell types we generated can potentially inform variant-to-function studies of cardiometabolic traits, we used CELLECT[58] to integrate our snRNA-seq data with relevant genome-wide association study (GWAS) datasets. We found genes associated with random blood glucose levels[59] enriched mostly in the AGRP$^+$/OTP$^+$ cluster (Figure 4A), aligning with rodent findings.[60] Interestingly, the most enriched clusters for body mass index[61] (BMI) and T2D[62] were UNC13C$^+$/OTP$^+$ and immature ARC neurons. UNC13$^+$/OTP$^+$ could plausibly correspond to the orexigenic Sst/Unc13c subcluster of arcuate Sst-expressing neurons identified by Campbell et al.[40] Height[61] was included as negative control and showed significant enrichment only in tanycytes, which are part of the hypothalamic-pituitary-somatotropic axis controlling body growth.[63]

We next assessed the functionality of the hPSC-derived ARC neurons by analyzing peptide production and response. ARC cultures progressively increased AGRP secretion over time, up to 177.1 ±3 6.7 pg/mL at day 70 (mean ± SEM, Figure 4B). To test functional regulation of AGRP, we stimulated ARC spheroids with the orexigenic peptide ghrelin[64] and detected increased levels of AGRP in the medium after 4 h (Figure 4C). Both baseline and ghrelin-induced AGRP release were inhibited by the ghrelin receptor antagonist liver-expressed antimicrobial peptide 2 (LEAP2).[65] To analyze immediate responses of ARC cultures to relevant anorexigenic peptides, we stimulated the cultures with either leptin or GLP-1 (7–37)[66] and analyzed their calcium response. For calcium recordings, peptides were added after a baseline recording followed by a depolarizing stimulus of potassium chloride to confirm the electrophysiological response potential (Figures 4D–4I). Across several biological replicates, an average of 55.5% ± 23.8% (mean ± SD) of cells were responsive to leptin and 8.7% ± 10.4% responded to GLP-1 (Figures 4F and 4G). These numbers were in line with the proportions of cells expressing the relevant receptors according to our snRNA-seq data, i.e., *LEPR* (45.8% ±12.9% of all cells, mean ± SD) and *GLP1R* (3.0% ± 0.6% of all cells) (Figures 4J and S4E). Comparison to the adult human ARC reference with a comparable total transcript count showed similar proportions of receptor-expressing cells (Figures 4J and S4F). To identify cell-type-specific calcium responses, we correlated the calcium traces with ICC and detected significant responses to leptin in both POMC$^+$ neurons and VIM$^+$ tanycytes (Figures 4K and 4L), both of which express *LEPR* in ~50% within their respective snRNA-seq clusters (Figures S4G and S4H). In summary, our data indicate that the hPSC-derived ARC cultures produce relevant neuropeptides and respond to key appetite-regulating peptides.

## Duration of BMP stimulation controls ARC versus VMH identity

Previous data from the chick has shown that a BMP wave in the early embryo is responsible for specifying the tuberal hypothalamus into an anterior RAX$^+$/SHH$^+$/POMC$^+$ domain and a posterior FGF10$^+$/TBX3$^+$/RAX$^+$ domain[34] (Figure 5A). We therefore investigated whether this developmental phenomenon could be recapitulated in our human *in vitro* model by changing the exposure to BMP (Figures 5B and S5A). We initiated BMP4 treatment of the cells at either days 3, 4, 5, or 6 and kept it until day 14 (Figure S5A). While *RAX* was highly expressed in all conditions at day 16, only the delayed addition of BMP4 at days 5 or 6 induced high levels of the posterior tuberal markers *TBX3* and *FGF10* (Figure S5B). Early BMP4 addition at day 3 by contrast caused a shift toward the *PAX6*$^+$/*VSX2*$^+$ eye field (Figures S5B–S5D), which is located anteriorly to the tuberal hypothalamus *in vivo*.[67] Comparison of the BMP conditions after day 50 showed that while all tuberal progenitors could generate POMC neurons, only the cultures stimulated with BMP4 later than day 3 could generate AGRP neurons (Figures S5E and S5F). We concluded that *FGF10*$^+$/*TBX3*$^+$ progenitors are required for generating AGRP neurons, and that initiation BMP on day 5 was optimal for obtaining these cells without inducing contaminating eye-field cells.

To manipulate BMP withdrawal, we removed BMP at different time points (days 7, 9, 11, or 14) (Figure 5B) after adding it from day 5. While long BMP4 exposure (days 11 and 14) resulted in progenitors expressing *RAX*, *TBX3*, and *FGF10*, short BMP4 exposure (days 7 or 9) caused significantly lower expression of posterior tuberal markers. Instead, the anterior marker *SHH* was upregulated together with the VMH markers NR5A1 and *SOX14* (Figures 5C, 5D, and S5G). These anterior progenitors did not give rise to AGRP neurons or tanycytes but were positive for characteristic VMH markers (NR5A1, *SOX14*, and *GPR149*) (Figures 5C, 5E, and S5H). Prolonged BMP4 exposure caused a gradual decrease in cell proliferation, which correlates with findings in the chick[34]; exposure beyond day 14 (i.e., >9 days

(J) % of cells expressing receptors in days 50 + 70 cultures ($n$ = 3) and adult ARC reference[20] ($n$ = 6).
(K) Experimental workflow for calcium imaging and ICC correlation.
(L) AUC for leptin-stimulated POMC$^+$ neurons ($n$ = 5; $n$ = 4 RC17, $n$ = 1 BIONi010) and VIM$^+$/HuC/D$^-$ tanycytes ($n$ = 3; RC17). Each data point = single cell. Tukey's boxplot, paired sample $t$ test on mean AUC per replicate (POMC$^+$ $p$ = 0.031, tanycytes $p$ = 0.047).

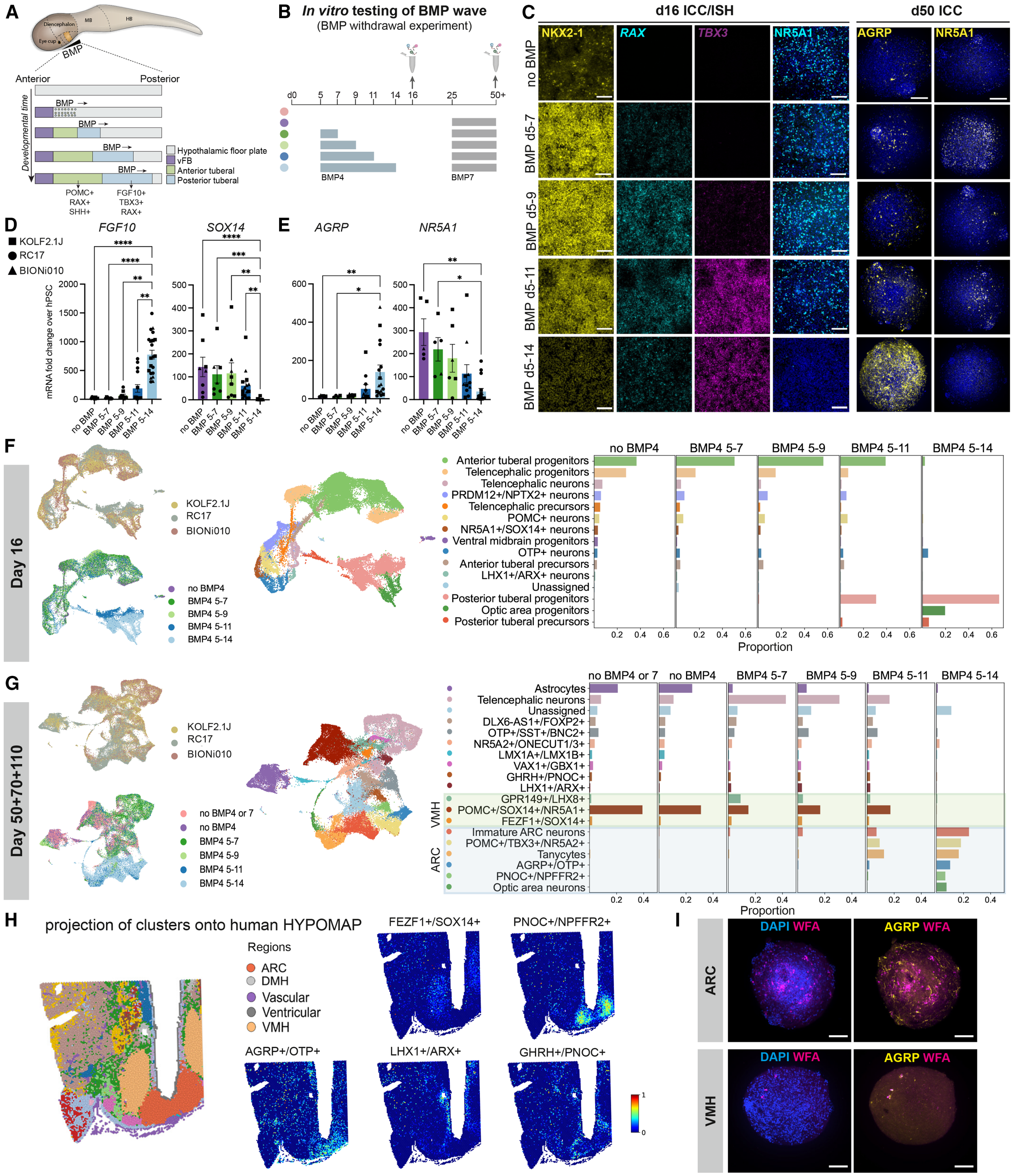


**Figure 5. Duration of BMP stimulation controls ARC versus VMH identity**

(A) BMP signaling in the developing chick tuberal hypothalamus. Adapted from Chinnaiya et al.[34] vFB, ventral forebrain.

(B) Experimental design to simulate BMP withdrawal.

(C) ICC/ISH for days 16 and 50 across BMP4 withdrawal conditions. Scale bar: 100 μm.

(D) Day 16 RT-qPCR (RC17, KOLF2.1J, BIONi010) across BMP4 withdrawal conditions. Mean ± SEM, Kruskal-Wallis with Dunn's multiple comparisons test: *FGF10*: −BMP versus days 5–14 $p < 0.0001$; days 5–7 versus days 5–14 $p < 0.0001$; days 5–9 versus days 5–14 $p = 0.0040$; days 5–11 versus days 5–14 $p = 0.013$; *SOX14*: −BMP versus days 5–14 $p < 0.0001$; days 5–7 versus days 5–14 $p = 0.0004$; days 5–9 versus days 5–14 $p = 0.0011$; days 5–11 versus days 5–14 $p = 0.0020$.

(legend continued on next page)

continuous stimulation) caused a collapse of the cultures (Figure S6I). snRNA-seq of the different BMP4 paradigms at days 16 and 50/80/110 allowed a more detailed view of the culture composition (Figures 5F, 5G, and S6). Already at day 16, cells treated with no or only short BMP4 exposure were transcriptionally different and contained anterior tuberal progenitors together with telencephalic cells and hypothalamic neurons with markers specific for the VMH (Figure 5F; Table S7). These anterior tuberal progenitors did not mature into the ARC-specific clusters, but into FEZF1$^{+}$/SOX14$^{+}$ and GPR149$^{+}$/LHX8$^{+}$ clusters (Figure 5G). The posterior tuberal progenitors, by contrast, gave rise to ARC-specific subtypes such as tanycytes and AGRP$^{+}$/OTP$^{+}$ neurons—clusters that were absent under short BMP conditions. When treated with no or short BMP during patterning, progenitors gave rise to astrocytes instead of tanycytes, independently of late-stage addition of BMP7 (Figures 5B and 5G). In the neuronal subclusters, increasing expression of action-potential-associated ion channels, neurotransmitter, and neuropeptide receptors could be detected in the cultures from days 50 to 110 (Figure S6J). Notably, both anterior and posterior progenitors gave rise to *POMC*$^{+}$ cells, although the anterior POMC cluster co-expressed *SOX14* and *NR5A1*. Spatial mapping to the human scRNA-seq reference dataset HYPOMAP[20] indicated that the FEZF1$^{+}$/SOX14$^{+}$ and LHX1$^{+}$/ARX$^{+}$ clusters from short BMP conditions located to VMH and DMH identities, respectively, whereas clusters from long BMP conditions like AGRP$^{+}$/OTP$^{+}$ or PNOC$^{+}$/NPFFR2$^{+}$ predominantly mapped to the ARC (Figure 5H). We also detected evidence of perineural nets (PNNs), an extracellular matrix structure that forms postnatally in the ARC,[68] in ARC but not VMH spheroids (Figure 5I). We confirmed that the temporal effect of BMP was robust across multiple hPSC lines, including RC17 hESC and two hiPSC lines, KOLF2.1 and BIONi010 (Figure S5K).

## Different POMC subtypes are dependent on BMP timing

We next investigated the identity of the two transcriptionally distinct POMC clusters, POMC$^{+}$/SOX14$^{+}$/NR5A1$^{+}$ derived from anterior tuberal progenitors with short BMP4 exposure and POMC$^{+}$/TBX3$^{+}$/NR5A2$^{+}$ derived from posterior tuberal progenitors with long BMP exposure (Figure 6A). To map their transcriptomic and spatial identity, we compared them with clusters expressing either *POMC* or VMH markers in several hypothalamic reference datasets[20,39] (Figures 6B and S7A). In addition, we incorporated two *POMC*$^{+}$ clusters from a human fetal brain dataset into the mapping; one positive for *TBX3* and the other for *NR5A1*.[69] When comparing their transcriptomic similarity,[50] the POMC$^{+}$/SOX14$^{+}$/NR5A1$^{+}$ cluster was most similar to fetal and adult VMH and the fetal POMC$^{+}$/NR5A1$^{+}$/TBX3$^{-}$ cluster (Figure 6C). The POMC$^{+}$/TBX3$^{+}$/NR5A2$^{+}$ cluster instead showed highest similarity to fetal POMC$^{+}$/TBX3$^{+}$/NR5A1$^{-}$ cells followed by adult POMC$^{+}$/PRDM12$^{+}$ neurons. To accurately assign our two *POMC* clusters to the spatial identity of hypothalamic nuclei, we used a spatial adult hypothalamic reference.[20] Generally, both ARC-associated (*AGRP*, *TBX3*, and *NR5A2*) and VMH-associated (*NR5A1* and *SOX14*) genes showed restricted expression in the spatial dataset while *POMC* was found mainly in the ventral ARC with some expression in the dorsal region extending into the VMH (Figure 6D). Out of all differentially expressed genes (DEGs) in the POMC$^{+}$/SOX14$^{+}$/NR5A1$^{+}$ cluster, 35 were enriched in the VMH in the spatial reference, while the POMC$^{+}$/TBX3$^{+}$/NR5A2$^{+}$ cluster expressed markers enriched in the ARC[70] (Figure 6E; Table S7). Accordingly, when mapped onto the spatial dataset, the POMC$^{+}$/SOX14$^{+}$/NR5A1$^{+}$ cells predominantly mapped to more dorsolateral regions of the hypothalamus, including the VMH, while POMC$^{+}$/TBX3$^{+}$/NR5A2$^{+}$ cells primarily co-localized with the ARC in the most medial-ventral part of the hypothalamus (Figure 6F).

Our BMP timing experiment indicated that the VMH-like subtype of POMC neurons could arise from cultures biased toward telencephalic fates, whereas the ARC-like subtype of POMC neurons was found only in BMP-patterned ARC cultures. In line with this, we detected large proportions of POMC$^{+}$/SOX14$^{-}$ $^{+}$/NR5A1$^{+}$ neurons ($\sim$40%) in forebrain-patterned cultures, which had not been exposed to any BMP (Figure 5G). To interrogate the developmental origins of POMC neurons in an *in vivo* system, we turned toward the chick as a model organism, which is easily accessible during early development. Indeed, the developing chick brain contains two spatially distinct developing *POMC*$^{+}$ populations, one located in the anterior ventral *ISL1*$^{+}$/*SIX6*$^{+}$ domain and another in the posterior-lateral tuberal hypothalamus (Figure 6G). These populations were observed at both Hamburger-Hamilton (HH) stages 17 and 20 providing support for the existence of at least two developmentally distinct origins of hypothalamic POMC neurons. Consistent with this, scRNA-seq data from the developing mouse hypothalamus[71] spanning between E11 and P8 revealed *Pomc* expression overlapping with regional markers of both the ARC and VMH (Figures S7B–S7D). Based on our stem cell model, we hypothesize a lineage model in which two different subtypes of POMC neurons arise from the anterior versus posterior tuberal domain and that only the posterior *POMC*$^{+}$ population corresponds to the ARC-like POMC subtypes found *in vivo* (Figure 7).

## DISCUSSION

The generation of stem-cell-derived *in vitro* models of the hypothalamus has been hindered by limited resolution of subregional lineage trajectories and the absence of definitive markers for lineage-committed progenitors. Although selected hypothalamic lineages have been delineated through fate-mapping,[72,73] the developmental origins of most neuronal subtypes remain unresolved, with progenitor-descendant relationships inferred largely from static anatomical gene expression

(E) Day 50 RT-qPCR (RC17, KOLF2.1J, BIONi010) across BMP4 withdrawal conditions. Mean ± SEM, Kruskal-Wallis with Dunn's test: *AGRP*: −BMP versus days 5–14 $p = 0.0080$; days 5–7 versus days 5–14 $p = 0.0269$ *NR5A1*: −BMP versus days 5–14 $p = 0.0016$; days 5–7 versus days 5–14 $p = 0.0162$.

(F and G) UMAPs of day 16 (F) and days 50 + 80 + 110 (G) snRNA-seq ($n = 3$; RC17, KOLF2.1J, BIONi010) and annotated clusters with proportional representation. ARC- and VMH-specific clusters are marked with a box.

(H) Spatial projection probability of days 50 + 80 + 110 clusters onto human adult reference.[20]

(I) 3D staining for Wisteria floribunda agglutinin (WFA), scale bar: 100 μm.

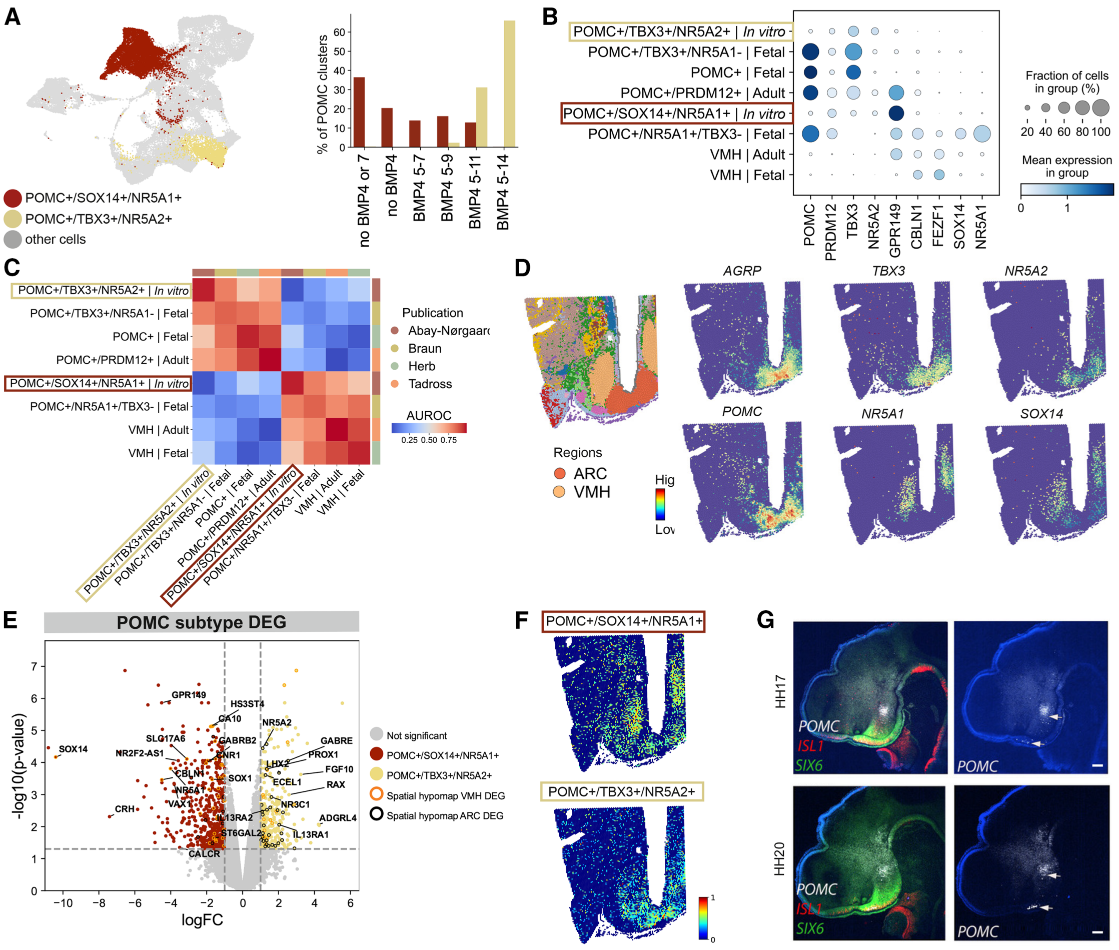


**Figure 6. Different POMC subtypes are dependent on BMP timing**

(A) UMAP of days 50 + 80 + 110 snRNA-seq data from Figure 5G with POMC clusters and their % across BMP4 exposure timings.

(B) Key marker genes across POMC$^{+}$ ARC and VMH clusters in *in vitro* dataset and human fetal and adult reference datasets.[20,39,69]

(C) MetaNeighbor cluster similarity analysis comparing *in vitro* POMC$^{+}$ clusters to hypothalamic reference datasets.[20,39,69]

(D) Expression of ARC/VMH-related markers on spatial transcriptomics reference.[20]

(E) DEG between *in vitro* POMC$^{+}$ subtypes, spatial DEGs from HYPOMAP[20] are highlighted. Benjamini-Hochberg corrected *p* values.

(F) Prediction probabilities from spatial mapping of *in vitro* POMC clusters to HYPOMAP.[20]

(G) Hybridization chain reaction (HCR) of chick embryo heads at HH17 and HH20, *n* = 7–10. Scale bar: 100 μm.

domains. Recent *in vivo* fate-mapping in the chick has reshaped prevailing concepts of forebrain organization, culminating in the proposal of a "tripartite hypothalamus" model.[25] This new model demonstrates that pronounced anisotropic growth distorts early topological relationships and obscures the spatial linkage between hypothalamic progenitors and their differentiated progeny.

Stem-cell-based *in vitro* systems offer a powerful complementary platform to address this knowledge gap, enabling longitudinal lineage resolution in the absence of the anatomical folding and interregional cell movements that characterize *in vivo* hypothalamic development. Here, we show through stem cell differentiation that the derivation of the human ARC lineage was highly dependent on the timing of BMP stimulation, comparable to the anterior-to-posterior BMP wave, which has been identified in the chick tuberal hypothalamus.[34] As in the chick,[34,35,38] we found that the induction of *TBX3* by BMP was essential for downregulation of *SHH* and upregulation of *FGF10* to obtain correctly patterned posterior tuberal hypothalamic fates. While the study in the chick did not uncover the resulting adult hypothalamic nuclei or neuronal subtypes arising from each tuberal progenitor domain, our human *in vitro* system demonstrates that the two tuberal progenitor domains are likely to underlie the developmental origins of the VMH and the ARC, respectively.

In-depth snRNA-seq analysis of our cultures revealed that while AGRP neurons and tanycytes were dependent on long BMP exposure, POMC neurons could be generated independent from BMP stimulation. More interestingly, different durations of BMP exposure yielded different POMC subtypes, one transcriptionally similar to ARC and the other to VMH. Previous groups have reported POMC neurons as a heterogeneous population,[17,20,74] and the *POMC* gene has been reported to be developmentally expressed not only in POMC neurons but also in other ARC neurons such as AGRP or Kisspeptin.[24,75,76] While both our *in vitro*-derived POMC populations expressed *PRDM12*, we found the main distinguishing markers to be *NR5A2*/*TBX3* for

CellPress
OPEN ACCESS

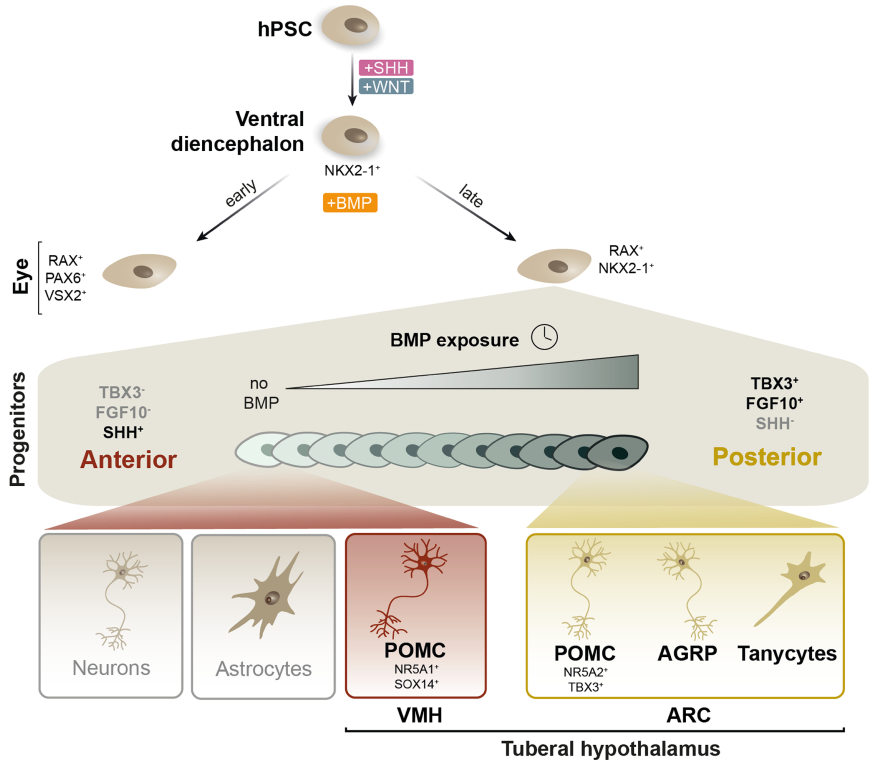


**Figure 7. BMP signaling controls antero-posterior patterning of the human tuberal hypothalamus**

Dual SMAD inhibition for neural induction combined with WNT and SHH signaling directs hPSC toward a ventral diencephalic identity. Early BMP induction specifies optic progenitors, while late BMP promotes NKX2-1+/RAX+ hypothalamic progenitors. The BMP duration determines anterior-posterior identity within the tuberal hypothalamus: short BMP exposure yields anterior progenitors with high SHH and low RAX/TBX3/FGF10, whereas prolonged BMP exposure promotes posterior identity with elevated RAX, TBX3, and FGF10 and reduced SHH. Anterior progenitors give rise to telencephalic (forebrain) neurons and astrocytes and NR5A1+/SOX14+ POMC neurons with a VMH identity, while posterior progenitors generate AGRP neurons, tanycytes and NR5A2+/TBX3+ POMC neurons with an ARC identity.

ARC-derived POMC and *NR5A1/SOX14/GPR149* for VMH-derived POMC.[77] Transcriptional mapping against human single-cell and spatial datasets suggests that the ARC-POMC is located medially around the median eminence, resembling the *PRDM12/ANXA2*-expressing POMC population previously described in human and mouse.[17,20,40] In contrast, the POMC+/SOX14+/NR5A1+ cluster correlates with the VMH and is located more laterally around the 3rd ventricle. This population possibly represent a previously uncharacterized POMC population although it remains unclear whether POMC expression is maintained in these cells into adulthood. A recent study reported *Pomc*-expressing cells in the developing mouse that express *Nr5a1* and are located within the VMH, suggesting a developmental POMC/NR5A1 subtype.[52] Our findings on different developmental origins of POMC neurons could potentially explain why previous protocols without BMP stimulation[26,27,32] could generate POMC neurons but lacked the presence of other important ARC-derived cell types such as AGRP, GHRH, and PNOC neurons. These previous protocols have been based on diencephalic patterning through high concentrations of SHH agonists,[29] in some cases combined with WNT inhibition to target anterior neural populations.[26–28] While these differentiation protocols have yielded high purity of NKX2-1+ progenitors, they have been heterogeneous in their subregional fates with the highest reported purity being 35% for RAX[27] and 10% for TBX3,[26] and evidence for regional identity through spatial mapping has not been provided. This emphasizes the importance of in-depth profiling of stem cell models and their progenitors to recapitulate the true developmental trajectory of specific cell types.

Upon maturation, our ARC cultures released AGRP and responded to key hormones including leptin, GLP-1, and ghrelin. Leptin, which in the adult is known to increase firing by depolarizing POMC neurons[77,78] and to cause intracellular calcium influx in tanycytes,[79] was found to cause a prolonged increase in calcium levels in our *in vitro*-generated POMC neurons and tanycytes. Interestingly, we were not able to detect leptin-induced phosphorylation of signal inducer and activator of transcription 3 (STAT3) despite repeated attempts (data not shown). We speculate that the cells *in vitro* may have not yet undergone the postnatal shift in leptin responsiveness, which is required for STAT3 phosphorylation,[80] and that truncated, developmental isoforms of the LEPR may be mediating the calcium transients.[81] The GWAS analysis for metabolic traits revealed enrichment in immature hypothalamic neurons, supporting previous studies suggesting that obesity may be partly caused by abrogated neurodevelopment of the hypothalamus.[82–84] Thereby, our model is not only relevant for physiological appetite regulation but also as a potential tool to link disease-linked variants to cellular or functional changes in hypothalamic development.

In summary, we here developed protocols by screening *in vitro* differentiations with transcriptomic profiling to dissect how the different morphogens affect the cellular identity. This framework can generate invaluable understanding of how individual components of the protocol shape the identity of the cultures to optimize differentiation efforts. Our resulting VMH and ARC protocols and proof-of-concept functional experiments provide a foundation for investigating human hypothalamic development and cellular responses to neuropeptide stimulation. Access to *in vitro* models of this type will enable detailed investigation of the cellular and molecular properties of appetite-regulating cells as well as CRISPR-based perturbation screens of obesity-associated genetic loci to identify and characterize hypothalamic energy homeostatic pathways in humans.

## Limitations of the study

While we have extensively characterized the hPSC-derived cultures in this study through single-cell transcriptomics, ICC,

ELISA, RNAscope, and functionality testing, there were also validations we were not able to perform. PNOC expression was validated only at RNA level as we could not identify a functioning antibody or ELISA for this target. Likewise, we could not identify ELISA kits that were able to validate release of TRH, α-MSH, and GHRH in cell culture medium although several kits were tested. NPY expression *in vitro* was variable and not found in all differentiation replicates. Finally, we did not detect induction of STAT3 phosphorylation upon leptin stimulation despite repeated attempts. Generally, stem-cell-derived neural cultures are likely to be more similar to fetal than adult brain, and this may be particularly important in the case of the hypothalamus, given that postnatal hormone surges are known to affect hypothalamic function and maturation.

## RESOURCE AVAILABILITY

### Lead contact

Further information and requests for resources and reagents should be directed to and will be fulfilled by the lead contact, Agnete Kirkeby (agnete.kirkeby@sund.ku.dk).

### Materials availability

This study generated no new unique reagents beyond the hPSC-derived forebrain progenitors, neurons, astrocytes, and tanycytes described here. Unique materials generated in this study are available from the lead contact upon reasonable request and completion of a material transfer agreement.

### Data and code availability

All data necessary for the conclusions of the study are provided with the article. sc/snRNA-seq data are available at GEO under SuperSeries accession number GEO: GSE271235 (ARC protocol) and GSE296407 (BMP timing). DEG lists are available in Table S7. The scripts used to analyze the sc/snRNA-seq data are available at GitHub (https://github.com/kirkebylab/In_vitro_ARC_VMH_analysis) and Zenodo (https://doi.org/10.5281/zenodo.20118475).

## ACKNOWLEDGMENTS

We want to thank Helle Lilja-Fischer, Kristoffer L. Egerod, and SCOP at CBMR for technical support on scRNA-seq experiments; the Microscopy and Genomics platform at reNEW Copenhagen and especially Jonas Viswalingam Bagge and Arun Thiruvalluvan for help with imaging; Antonios Georgantzoglou for support setting up the image quantifications; Jette Pia Larsen, Theofania Tsitsopoulou, and Sonia Araujo for technical assistance; Oksana Dmytriyeva for help with WFA staining; Novo Nordisk A/S for advice and for providing LEAP2 peptide as a gift; Gaurav Rathore for advice on bioinformatic analysis; and Lennart V. Schneider for designing graphics. This study was supported by the Danish Research Council (grant no. 0169-00073B to A.K., 8045-00091B to T.H.P.), the European Union (H2020, NSC-Reconstruct GA no. 874758), the Novo Nordisk Foundation (NNF23SA0088590 to A.K. and NNF16OC0021496 to T.H.P.), the Wellcome Trust (grant nos. [212247/Z/18/Z and 303188/Z/23/Z to M.P.), the Lundbeck Foundation (R350-2020-963 and R380-2021-1267 to A.K. and R190-2014-3904 to T.H.P.), and the Knut and Alice Wallenberg Foundation. reNEW and CBMR are supported by donations from the Novo Nordisk Foundation (grant number NNF21CC0073729 and NNF23SA0084103).

## AUTHOR CONTRIBUTIONS

A.K., Z.A.-N., A.K.M., and E.H. conceived the study. Z.A.-N., A.K.M., L.S.T., J.B.C., V.N., S.S., and S.P. performed the *in vitro* differentiations and downstream analyses. A.K.M., B.H., and J.H. performed Parse sequencing. E.H., D.R., N.W.M., and Y.L. performed general bioinformatic analyses. L.S.T., L.F., and Q.Q. performed image quantifications. A.S. and A.L.S. performed *in vivo* transplantation of cells, and L.P. did analysis of rat brain sections. J.K., L.F., A.K.M., and L.S.T. performed the calcium imaging. K.C. and M.P. performed chick ISH. Z.A.-N., A.K.M., L.S.T., S.P., S.S., J.B.C., and C.H.K. collected the data, and A.K., Z.A.-N., A.K.M., and E.H. wrote the manuscript with input from all authors.

## DECLARATION OF INTERESTS

A.K., Z.A.-N., and J.B.C. are co-inventors on submitted patent applications related to the generation of human hypothalamic cell types from stem cells. Two authors of the study, D.R. and B.H., have after their contribution to the study transferred into positions at the private company Novo Nordisk A/S. A.K. owns a consultancy company Kirkeby Cell Therapy APS, which performs paid consultancy for Somite Therapeutics (now Cellular Intelligence) and CCRM Nordic and which has previously performed consultancy for Novo Nordisk A/S. L.S.T. has been partly funded by a collaboration grant from Novo Nordisk A/S during the study, and T.H.P. and A.K. have received funding from Novo Nordisk A/S for conducting other collaboration research projects. T.H.P. has received consultancy fees from Eli Lilly and Zealand Pharma.

## STAR★METHODS

Detailed methods are provided in the online version of this paper and include the following:

- KEY RESOURCES TABLE
- EXPERIMENTAL MODEL AND STUDY PARTICIPANT DETAILS
  - Human Pluripotent Stem Cell Lines
  - Athymic nude rats
  - Avian Embryos
- METHOD DETAILS
  - Ethics declaration
  - Cell culture and neuronal differentiation
  - Cryopreservation and thawing of progenitors
  - Xenotransplantation of d16 ARC progenitors
  - RNA Extraction and qRT-PCR
  - Expression matrix analysis from qPCR data
  - Immunocytochemistry (ICC)
  - Multiplexed immunohistochemistry
  - ISH on cell cultures
  - ISH/IF on brain sections
  - Chick collection and HCR
  - Imaging and processing
  - AGRP ELISA
  - FGF1 stimulation experiment
  - Calcium Imaging
  - Calcium imaging analysis
  - Single-cell/nucleus isolation for sequencing
  - 10X barcoding for sc/sn-RNAseq
  - Parse barcoding for snRNA-seq
  - Sequence alignment and sample assignment
  - sc/snRNA-seq processing and clustering
  - Trajectory inference
  - Differential gene expression analysis
  - Transcriptomic comparison with human data
  - Analysis of fetal mouse hypothalamic data
  - CELLECT analysis
  - Statistical analysis

## SUPPLEMENTAL INFORMATION

Supplemental information can be found online at https://doi.org/10.1016/j.stem.2026.05.005.

Received: June 26, 2025
Revised: March 4, 2026
Accepted: May 14, 2026
Published: June 8, 2026

## REFERENCES

1. Brüning, J.C., and Fenselau, H. (2023). Integrative neurocircuits that control metabolism and food intake. Science *381*, eabl7398. https://doi.org/10.1126/science.abl7398.
2. Saper, C.B., and Lowell, B.B. (2014). The hypothalamus. Curr. Biol. *24*, R1111–R1116. https://doi.org/10.1016/j.cub.2014.10.023.
3. Myers, M.G., Jr., Affinati, A.H., Richardson, N., and Schwartz, M.W. (2021). Central nervous system regulation of organismal energy and glucose homeostasis. Nat. Metab. *3*, 737–750. https://doi.org/10.1038/s42255-021-00408-5.
4. Zhang, J., Chen, D., Sweeney, P., and Yang, Y. (2020). An excitatory ventromedial hypothalamus to paraventricular thalamus circuit that suppresses food intake. Nat. Commun. *11*, 6326. https://doi.org/10.1038/s41467-020-20093-4.
5. Tu, L., Fukuda, M., Tong, Q., and Xu, Y. (2022). The ventromedial hypothalamic nucleus: watchdog of whole-body glucose homeostasis. Cell Biosci. *12*, 71. https://doi.org/10.1186/s13578-022-00799-2.
6. Benevento, M., Alpár, A., Gundacker, A., Afjehi, L., Balueva, K., Hevesi, Z., Hanics, J., Rehman, S., Pollak, D.D., Lubec, G., et al. (2024). A brainstem–hypothalamus neuronal circuit reduces feeding upon heat exposure. Nature *628*, 826–834. https://doi.org/10.1038/s41586-024-07232-3.
7. Bolborea, M., and Dale, N. (2013). Hypothalamic tanycytes: potential roles in the control of feeding and energy balance. Trends Neurosci. *36*, 91–100. https://doi.org/10.1016/j.tins.2012.12.008.
8. Prevot, V., Dehouck, B., Sharif, A., Ciofi, P., Giacobini, P., and Clasadonte, J. (2018). The Versatile Tanycyte: A Hypothalamic Integrator of Reproduction and Energy Metabolism. Endocr. Rev. *39*, 333–368. https://doi.org/10.1210/er.2017-00235.
9. Johansen, V.B.I., Petersen, J., Lund, J., Mathiesen, C.V., Fenselau, H., and Clemmensen, C. (2025). Brain control of energy homeostasis: Implications for anti-obesity pharmacotherapy. Cell *188*, 4178–4212. https://doi.org/10.1016/j.cell.2025.06.010.
10. Vear, A., Heneka, M.T., and Clemmensen, C. (2025). Incretin-based therapeutics for the treatment of neurodegenerative diseases. Nat. Metab. *7*, 679–696. https://doi.org/10.1038/s42255-025-01263-4.
11. Dong, Y., Carty, J., Goldstein, N., He, Z., Hwang, E., Chau, D., Wallace, B., Kabahizi, A., Lieu, L., Peng, Y., et al. (2021). Time and metabolic state-dependent effects of GLP-1R agonists on NPY/AgRP and POMC neuronal activity in vivo. Mol. Metab. *54*, 101352. https://doi.org/10.1016/j.molmet.2021.101352.
12. Rupp, A.C., Tomlinson, A.J., Affinati, A.H., Yacawych, W.T., Duensing, A.M., True, C., Lindsley, S.R., Kirigiti, M.A., MacKenzie, A., Polex-Wolf, J., et al. (2023). Suppression of food intake by Glp1r/Lepr-coexpressing neurons prevents obesity in mouse models. J. Clin. Investig. *133*, e157515. https://doi.org/10.1172/JCI157515.
13. Sisley, S., Gutierrez-Aguilar, R., Scott, M., D'Alessio, D.A., Sandoval, D.A., and Seeley, R.J. (2014). Neuronal GLP1R mediates liraglutide's anorectic but not glucose-lowering effect. J. Clin. Investig. *124*, 2456–2463. https://doi.org/10.1172/JCI72434.
14. Ludwig, M.Q., Cheng, W., Gordian, D., Lee, J., Paulsen, S.J., Hansen, S.N., Egerod, K.L., Barkholt, P., Rhodes, C.J., Secher, A., et al. (2021). A genetic map of the mouse dorsal vagal complex and its role in obesity. Nat. Metab. *3*, 530–545. https://doi.org/10.1038/s42255-021-00363-1.
15. Jais, A., and Brüning, J.C. (2022). Arcuate Nucleus-Dependent Regulation of Metabolism—Pathways to Obesity and Diabetes Mellitus. Endocr. Rev. *43*, 314–328. https://doi.org/10.1210/endrev/bnab025.
16. Kim, D.W., Place, E., Chinnaiya, K., Manning, E., Sun, C., Dai, W., Groves, I., Ohyama, K., Burbridge, S., Placzek, M., et al. (2022). Single-cell analysis of early chick hypothalamic development reveals that hypothalamic cells are induced from prethalamic-like progenitors. Cell Rep. *38*, 110251. https://doi.org/10.1016/j.celrep.2021.110251.
17. Steuernagel, L., Lam, B.Y.H., Klemm, P., Dowsett, G.K.C., Bauder, C.A., Tadross, J.A., Hitschfeld, T.S., del Rio Martin, A., Chen, W., de Solis, A.J., et al. (2022). HypoMap—a unified single-cell gene expression atlas of the murine hypothalamus. Nat. Metab. *4*, 1402–1419. https://doi.org/10.1038/s42255-022-00657-y.
18. Placzek, M., Chinnaiya, K., Kim, D.W., and Blackshaw, S. (2025). Control of tuberal hypothalamic development and its implications in metabolic disorders. Nat. Rev. Endocrinol. *21*, 118–130. https://doi.org/10.1038/s41574-024-01036-1.
19. Kim, D.W., Washington, P.W., Wang, Z.Q., Lin, S.H., Sun, C., Ismail, B.T., Wang, H., Jiang, L., and Blackshaw, S. (2020). The cellular and molecular landscape of hypothalamic patterning and differentiation from embryonic to late postnatal development. Nat. Commun. *11*, 4360. https://doi.org/10.1038/s41467-020-18231-z.
20. Tadross, J.A., Steuernagel, L., Dowsett, G.K.C., Kentistou, K.A., Lundh, S., Porniece, M., Klemm, P., Rainbow, K., Hvid, H., Kania, K., et al. (2025). A comprehensive spatio-cellular map of the human hypothalamus. Nature *639*, 708–716. https://doi.org/10.1038/s41586-024-08504-8.
21. Puelles, L., and Rubenstein, J.L.R. (2015). A new scenario of hypothalamic organization: rationale of new hypotheses introduced in the updated prosomeric model. Front. Neuroanat. *9*, 27. https://doi.org/10.3389/fnana.2015.00027.
22. Bedont, J.L., Newman, E.A., and Blackshaw, S. (2015). Patterning, specification, and differentiation in the developing hypothalamus. Wiley Interdiscip. Rev. Dev. Biol. *4*, 445–468. https://doi.org/10.1002/wdev.187.
23. Ma, T., Wong, S.Z.H., Lee, B., Ming, G.L. li, and Song, H. (2021). Decoding neuronal composition and ontogeny of individual hypothalamic nuclei. Neuron *109*, 1150–1167.e6. https://doi.org/10.1016/j.neuron.2021.01.026.
24. Padilla, S.L., Carmody, J.S., and Zeltser, L.M. (2010). Pomc-expressing progenitors give rise to antagonistic neuronal populations in hypothalamic feeding circuits. Nat. Med. *16*, 403–405. https://doi.org/10.1038/nm.2126.
25. Manning, E., Chinnaiya, K., Furley, C., Kim, D.W., Blackshaw, S., Placzek, M., and Place, E. (2025). Resolving forebrain developmental organisation by analysis of differential growth patterns. Nat. Commun. *17*, 901. https://doi.org/10.1038/s41467-025-67623-6.
26. Huang, W.-K., Wong, S.Z.H., Pather, S.R., Nguyen, P.T.T., Zhang, F., Zhang, D.Y., Zhang, Z., Lu, L., Fang, W., Chen, L., et al. (2021). Generation of hypothalamic arcuate organoids from human induced pluripotent stem cells. Cell Stem Cell *28*, 1657–1670.e10. https://doi.org/10.1016/j.stem.2021.04.006.
27. Rajamani, U., Gross, A.R., Hjelm, B.E., Sequeira, A., Vawter, M.P., Tang, J., Gangalapudi, V., Wang, Y., Andres, A.M., Gottlieb, R.A., et al. (2018). Super-Obese Patient-Derived iPSC Hypothalamic Neurons Exhibit Obesogenic Signatures and Hormone Responses. Cell Stem Cell *22*, 698–712.e9. https://doi.org/10.1016/j.stem.2018.03.009.
28. Merkle, F.T., Maroof, A., Wataya, T., Sasai, Y., Studer, L., Eggan, K., and Schier, A.F. (2015). Generation of neuropeptidergic hypothalamic neurons from human pluripotent stem cells. Development *142*, 633–643. https://doi.org/10.1242/dev.117978.
29. Wang, L., Meece, K., Williams, D.J., Lo, K.A., Zimmer, M., Heinrich, G., Martin Carli, J., Leduc, C.A., Sun, L., Zeltser, L.M., et al. (2015). Differentiation of hypothalamic-like neurons from human pluripotent stem cells. J. Clin. Investig. *125*, 796–808. https://doi.org/10.1172/JCI79220.
30. Shen, Y., Wong, S.Z.H., Ma, T., Zhang, F., Wang, Q., Kawaguchi, R., Geschwind, D.H., Wang, J., He, C., Ming, G.L. li, et al. (2025). m6A deficiency impairs hypothalamic neurogenesis of feeding-related neurons in mice and human organoids and leads to adult obesity in mice. Cell Stem Cell *32*, 727–743.e8. https://doi.org/10.1016/j.stem.2025.02.011.
31. Miwata, T., Suga, H., Kawaguchi, Y., Sakakibara, M., Kano, M., Taga, S., Soen, M., Ozaki, H., Asano, T., Sasaki, H., et al. (2023). Generation of hypothalamic neural stem cell-like cells in vitro from human pluripotent

stem cells. Stem Cell Rep. *18*, 869–883. https://doi.org/10.1016/j.stemcr.2023.02.006.

32. Sarrafha, L., Neavin, D.R., Parfitt, G.M., Kruglikov, I.A., Whitney, K., Reyes, R., Coccia, E., Kareva, T., Goldman, C., Tipon, R., et al. (2023). Novel human pluripotent stem cell-derived hypothalamus organoids demonstrate cellular diversity. iScience *26*, 107525. https://doi.org/10.1016/j.isci.2023.107525.
33. Torz, L., Niss, K., Lundh, S., Rekling, J.C., Quintana, C.D., Frazier, S.E.D., Mercer, A.J., Cornea, A., Bertelsen, C.V., Gerstenberg, M.K., et al. (2022). NPFF Decreases Activity of Human Arcuate NPY Neurons: A Study in Embryonic-Stem-Cell-Derived Model. Int. J. Mol. Sci. *23*, 3260. https://doi.org/10.3390/ijms23063260.
34. Chinnaiya, K., Burbridge, S., Jones, A., Kim, D.W., Place, E., Manning, E., Groves, I., Sun, C., Towers, M., Blackshaw, S., et al. (2023). A neuroepithelial wave of BMP signalling drives anteroposterior specification of the tuberal hypothalamus. eLife *12*, e83133. https://doi.org/10.7554/eLife.83133.
35. Chinnaiya, K., Groves, I., Manning, E., Place, E., Furley, C., Strawbridge, S.E., Fletcher, A.G., and Placzek, M. (2025). Balancing SHH and BMP/FGF10 to specify tuberal hypothalamic neurons and glia. Dev. Biol. *525*, 259–269. https://doi.org/10.1016/j.ydbio.2025.06.008.
36. Nordström, U., Jessell, T.M., and Edlund, T. (2002). Progressive induction of caudal neural character by graded Wnt signaling. Nat. Neurosci. *5*, 525–532. https://doi.org/10.1038/nn0602-854.
37. Kirkeby, A., Grealish, S., Wolf, D.A., Nelander, J., Wood, J., Lundblad, M., Lindvall, O., and Parmar, M. (2012). Generation of Regionally Specified Neural Progenitors and Functional Neurons from Human Embryonic Stem Cells under Defined Conditions. Cell Rep. *1*, 703–714. https://doi.org/10.1016/j.celrep.2012.04.009.
38. Manning, L., Ohyama, K., Saeger, B., Hatano, O., Wilson, S.A., Logan, M., and Placzek, M. (2006). Regional Morphogenesis in the Hypothalamus: A BMP-Tbx2 Pathway Coordinates Fate and Proliferation through Shh Downregulation. Dev. Cell *11*, 873–885. https://doi.org/10.1016/j.devcel.2006.09.021.
39. Herb, B.R., Glover, H.J., Bhaduri, A., Colantuoni, C., Bale, T.L., Siletti, K., Hodge, R., Lein, E., Kriegstein, A.R., Doege, C.A., et al. (2023). Single-cell genomics reveals region-specific developmental trajectories underlying neuronal diversity in the human hypothalamus. Sci. Adv. *9*, eadf6251. https://doi.org/10.1126/sciadv.adf6251.
40. Campbell, J.N., Macosko, E.Z., Fenselau, H., Pers, T.H., Lyubetskaya, A., Tenen, D., Goldman, M., Verstegen, A.M.J., Resch, J.M., McCarroll, S.A., et al. (2017). A molecular census of arcuate hypothalamus and median eminence cell types. Nat. Neurosci. *20*, 484–496. https://doi.org/10.1038/nn.4495.
41. Solheim, M.H., Stroganov, S., Chen, W., Subagia, P.S., Bauder, C.A., Wnuk-Lipinski, D., Del Río-Martín, A., Sotelo-Hitschfeld, T., Beddows, C.A., Klemm, P., et al. (2025). Hypothalamic PNOC/NPY neurons constitute mediators of leptin-controlled energy homeostasis. Cell *188*, 3550–3566.e22. https://doi.org/10.1016/j.cell.2025.04.001.
42. Jais, A., Paeger, L., Sotelo-Hitschfeld, T., Bremser, S., Prinzensteiner, M., Klemm, P., Mykytiuk, V., Widdershooven, P.J.M., Vesting, A.J., Grzelka, K., et al. (2020). PNOCARC Neurons Promote Hyperphagia and Obesity upon High-Fat-Diet Feeding. Neuron *106*, 1009–1025.e10. https://doi.org/10.1016/j.neuron.2020.03.022.
43. Biebermann, H., Castañeda, T.R., van Landeghem, F., von Deimling, A., Escher, F., Brabant, G., Hebebrand, J., Hinney, A., Tschöp, M.H., Grüters, A., et al. (2006). A role for β-melanocyte-stimulating hormone in human body-weight regulation. Cell Metab. *3*, 141–146. https://doi.org/10.1016/j.cmet.2006.01.007.
44. Harno, E., Gali Ramamoorthy, T.G., Coll, A.P., and White, A. (2018). POMC: The physiological power of hormone processing. Physiol. Rev. *98*, 2381–2430. https://doi.org/10.1152/physrev.00024.2017.
45. Ogawa, K., Suga, H., Ozone, C., Sakakibara, M., Yamada, T., Kano, M., Mitsumoto, K., Kasai, T., Kodani, Y., Nagasaki, H., et al. (2018). Vasopressin-secreting neurons derived from human embryonic stem cells through specific induction of dorsal hypothalamic progenitors. Sci. Rep. *8*, 3615. https://doi.org/10.1038/s41598-018-22053-x.
46. Balthasar, N., Mery, P.F., Magoulas, C.B., Mathers, K.E., Martin, A., Mollard, P., and Robinson, I.C.A.F. (2003). Growth hormone-releasing hormone (GHRH) neurons in GHRH-enhanced green fluorescent protein transgenic mice: A ventral hypothalamic network. Endocrinology *144*, 2728–2740. https://doi.org/10.1210/en.2003-0006.
47. Sotelo-Hitschfeld, T., Minère, M., Klemm, P., Borgmann, D., Wnuk-Lipinski, D., Jais, A., Jia, X., Corneliussen, S., Kloppenburg, P., Fenselau, H., et al. (2024). GABAergic disinhibition from the BNST to PNOCARC neurons promotes HFD-induced hyperphagia. Cell Rep. *43*, 114343. https://doi.org/10.1016/j.celrep.2024.114343.
48. Tan, H.L., Yin, L., Tan, Y., Ivanov, J., Plucinska, K., Ilanges, A., Herb, B.R., Wang, P., Kosse, C., Cohen, P., et al. (2024). Leptin-activated hypothalamic BNC2 neurons acutely suppress food intake. Nature *636*, 198–205. https://doi.org/10.1038/s41586-024-08108-2.
49. Lavoie, O., Turmel, A., Mattoon, P., Desrosiers, W.J., Plamondon, J., Michael, N.J., and Caron, A. (2024). Hypothalamic GABAergic Neurons Expressing Cellular Retinoic Acid Binding Protein 1 (CRABP1) Are Sensitive to Metabolic Status and Liraglutide in Male Mice. Neuroendocrinology *114*, 681–697. https://doi.org/10.1159/000538716.
50. Crow, M., Paul, A., Ballouz, S., Huang, Z.J., and Gillis, J. (2018). Characterizing the replicability of cell types defined by single cell RNA-sequencing data using MetaNeighbor. Nat. Commun. *9*, 884. https://doi.org/10.1038/s41467-018-03282-0.
51. Lee, B., Kim, J., An, T., Kim, S., Patel, E.M., Raber, J., Lee, S.-K., Lee, S., and Lee, J.W. (2018). Dlx1/2 and Otp coordinate the production of hypothalamic GHRH- and AgRP-neurons. Nat. Commun. *9*, 2026. https://doi.org/10.1038/s41467-018-04377-4.
52. Xu, B., Li, L., Chen, M., Wu, Z., Chen, X., Swati, W., Wan, R., Almeida, A.G., Wyler, S.C., and Liu, C. (2026). Developmental reprogramming in melanocortin neurons modulates diet-induced obesity in mice. Neuron *114*, 1210–1218.e4. https://doi.org/10.1016/j.neuron.2025.12.022.
53. Langlet, F., Levin, B.E., Luquet, S., Mazzone, M., Messina, A., Dunn-Meynell, A.A., Balland, E., Lacombe, A., Mazur, D., Carmeliet, P., et al. (2013). Tanycytic VEGF-A Boosts Blood-Hypothalamus Barrier Plasticity and Access of Metabolic Signals to the Arcuate Nucleus in Response to Fasting. Cell Metab. *17*, 607–617. https://doi.org/10.1016/j.cmet.2013.03.004.
54. Kaminskas, B., Goodman, T., Hagan, A., Bellusci, S., Ornitz, D.M., and Hajihosseini, M.K. (2019). Characterisation of endogenous players in fibroblast growth factor-regulated functions of hypothalamic tanycytes and energy-balance nuclei. J. Neuroendocrinol. *31*, e12750. https://doi.org/10.1111/jne.12750.
55. Fong, H., and Kurrasch, D.M. (2023). Developmental and functional relationships between hypothalamic tanycytes and embryonic radial glia. Front. Neurosci. *16*, 1129414. https://doi.org/10.3389/fnins.2022.1129414.
56. Bentsen, M.A., Rausch, D.M., Mirzadeh, Z., Muta, K., Scarlett, J.M., Brown, J.M., Herranz-Pérez, V., Baquero, A.F., Thompson, J., Alonge, K.M., et al. (2020). Transcriptomic analysis links diverse hypothalamic cell types to fibroblast growth factor 1-induced sustained diabetes remission. Nat. Commun. *11*, 4458. https://doi.org/10.1038/s41467-020-17720-5.
57. Brown, J.M., Bentsen, M.A., Rausch, D.M., Phan, B.A., Wieck, D., Wasanwala, H., Matsen, M.E., Acharya, N., Richardson, N.E., Zhao, X., et al. (2021). Role of hypothalamic MAPK/ERK signaling and central action of FGF1 in diabetes remission. iScience *24*, 102944. https://doi.org/10.1016/j.isci.2021.102944.
58. Timshel, P.N., Thompson, J.J., and Pers, T.H. (2020). Genetic mapping of etiologic brain cell types for obesity. eLife *9*, e55851. https://doi.org/10.7554/ELIFE.55851.
59. Qiao, Z., Sidorenko, J., Revez, J.A., Xue, A., Lu, X., Pärna, K., Snieder, H., , et al.; Lifelines Cohort Study, Visscher, P.M., Wray, N.R. (2023). Estimation and implications of the genetic architecture of fasting and

non-fasting blood glucose. Nat. Commun. *14*, 451. https://doi.org/10.1038/s41467-023-36013-1.

60. Gou, Y., Glat, M., Damian, V., Bryan, C.L., Phan, B.A., Faber, C.L., Trivedi, A., Hwang, M.K., Scarlett, J.M., Morton, G.J., et al. (2025). AgRP neuron hyperactivity drives hyperglycemia in a mouse model of type 2 diabetes. J. Clin. Investig. *135*, e189842. https://doi.org/10.1172/JCI189842.

61. Yengo, L., Sidorenko, J., Kemper, K.E., Zheng, Z., Wood, A.R., Weedon, M.N., Frayling, T.M., Hirschhorn, J., Yang, J., Visscher, P.M., et al. (2018). Meta-analysis of genome-wide association studies for height and body mass index in ∼700000 individuals of European ancestry. Hum. Mol. Genet. *27*, 3641–3649. https://doi.org/10.1093/hmg/ddy271.

62. Mahajan, A., Taliun, D., Thurner, M., Robertson, N.R., Torres, J.M., Rayner, N.W., Payne, A.J., Steinthorsdottir, V., Scott, R.A., Grarup, N., et al. (2018). Fine-mapping type 2 diabetes loci to single-variant resolution using high-density imputation and islet-specific epigenome maps. Nat. Genet. *50*, 1505–1513. https://doi.org/10.1038/s41588-018-0241-6.

63. Rodriguez-Cortes, B., Gomez-Martínez, R., Spenle, R., Schwaninger, M., Nogueiras, R., Rasika, S., and Prevot, V. (2025). Tanycytes: bloodhounds of the metabolic brain. Published online November 1, 2025. Trends Endocrinol. Metab. https://doi.org/10.1016/j.tem.2025.10.005.

64. Nakazato, M., Murakami, N., Date, Y., Kojima, M., Matsuo, H., Kangawa, K., and Matsukura, S. (2001). A role for ghrelin in the central regulation of feeding. Nature *409*, 194–198. https://doi.org/10.1038/35051587.

65. Ge, X., Yang, H., Bednarek, M.A., Galon-Tilleman, H., Chen, P., Chen, M., Lichtman, J.S., Wang, Y., Dalmas, O., Yin, Y., et al. (2018). LEAP2 Is an Endogenous Antagonist of the Ghrelin Receptor. Cell Metab. *27*, 461–469.e6. https://doi.org/10.1016/j.cmet.2017.10.016.

66. Secher, A., Jelsing, J., Baquero, A.F., Hecksher-Sørensen, J., Cowley, M.A., Dalbøge, L.S., Hansen, G., Grove, K.L., Pyke, C., Raun, K., et al. (2014). The arcuate nucleus mediates GLP-1 receptor agonist liraglutide-dependent weight loss. J. Clin. Investig. *124*, 4473–4488. https://doi.org/10.1172/JCI75276.

67. Marquardt, T., Ashery-Padan, R., Andrejewski, N., Scardigli, R., Guillemot, F., and Gruss, P. (2001). Pax6 Is Required for the Multipotent State of Retinal Progenitor Cells. Cell *105*, 43–55. https://doi.org/10.1016/S0092-8674(01)00295-1.

68. Mirzadeh, Z., Alonge, K.M., Cabrales, E., Herranz-Pérez, V., Scarlett, J.M., Brown, J.M., Hassouna, R., Matsen, M.E., Nguyen, H.T., Garcia-Verdugo, J.M., et al. (2019). Perineuronal net formation during the critical period for neuronal maturation in the hypothalamic arcuate nucleus. Nat. Metab. *1*, 212–221. https://doi.org/10.1038/s42255-018-0029-0.

69. Braun, E., Danan-Gotthold, M., Borm, L.E., Lee, K.W., Vinsland, E., Lönnerberg, P., Hu, L., Li, X., He, X., Andrusivová, Ž., et al. (2023). Comprehensive cell atlas of the first-trimester developing human brain. Science *382*, eadf1226. https://doi.org/10.1126/science.adf1226.

70. Atkin, S.D., Owen, B.M., Bookout, A.L., Cravo, R.M., Lee, C., Elias, C.F., Elmquist, J.K., Kliewer, S.A., and Mangelsdorf, D.J. (2013). Nuclear receptor LRH-1 induces the reproductive neuropeptide kisspeptin in the hypothalamus. Mol. Endocrinol. *27*, 598–605. https://doi.org/10.1210/me.2012-1371.

71. Kim, D.W., Duncan, L.H., Xu, Z., Chang, M., Sejer, S., Terrillion, C.E., Kanold, P.O., Place, E., and Blackshaw, S. (2025). Decoding gene networks controlling hypothalamic and prethalamic neuron development. Cell Rep. *44*, 115858. https://doi.org/10.1016/j.celrep.2025.115858.

72. Skidmore, J.M., Cramer, J.D., Martin, J.F., and Martin, D.M. (2008). Cre fate mapping reveals lineage specific defects in neuronal migration with loss of Pitx2 function in the developing mouse hypothalamus and subthalamic nucleus. Mol. Cell. Neurosci. *37*, 696–707. https://doi.org/10.1016/j.mcn.2007.12.015.

73. Alvarez-Bolado, G., Paul, F.A., and Blaess, S. (2012). Sonic hedgehog lineage in the mouse hypothalamus: from progenitor domains to hypothalamic regions. Neural Dev. *7*, 4. https://doi.org/10.1186/1749-8104-7-4.

74. Lam, B.Y.H., Cimino, I., Polex-Wolf, J., Nicole Kohnke, S., Rimmington, D., Iyemere, V., Heeley, N., Cossetti, C., Schulte, R., Saraiva, L.R., et al. (2017). Heterogeneity of hypothalamic pro-opiomelanocortin-expressing neurons revealed by single-cell RNA sequencing. Mol. Metab. *6*, 383–392. https://doi.org/10.1016/j.molmet.2017.02.007.

75. Sanz, E., Quintana, A., Deem, J.D., Steiner, R.A., Palmiter, R.D., and McKnight, G.S. (2015). Fertility-Regulating Kiss1 Neurons Arise from Hypothalamic *Pomc*-Expressing Progenitors. J. Neurosci. *35*, 5549–5556. https://doi.org/10.1523/JNEUROSCI.3614-14.2015.

76. Padilla, S.L., Reef, D., and Zeltser, L.M. (2012). Defining POMC neurons using transgenic reagents: Impact of transient Pomc expression in diverse immature neuronal populations. Endocrinology *153*, 1219–1231. https://doi.org/10.1210/en.2011-1665.

77. Cheung, C.C., Kurrasch, D.M., Liang, J.K., and Ingraham, H.A. (2013). Genetic labeling of steroidogenic factor-1 (SF-1) neurons in mice reveals ventromedial nucleus of the hypothalamus (VMH) circuitry beginning at neurogenesis and development of a separate non-SF-1 neuronal cluster in the ventrolateral VMH. J. Comp. Neurol. *521*, 1268–1288. https://doi.org/10.1002/cne.23226.

78. Cowley, M.A., Smart, J.L., Rubinstein, M., Cerdán, M.G., Diano, S., Horvath, T.L., Cone, R.D., and Low, M.J. (2001). Leptin activates anorexigenic POMC neurons through a neural network in the arcuate nucleus. Nature *411*, 480–484. https://doi.org/10.1038/35078085.

79. Duquenne, M., Folgueira, C., Bourouh, C., Millet, M., Silva, A., Clasadonte, J., Imbernon, M., Fernandois, D., Martinez-Corral, I., Kusumakshi, S., et al. (2021). Leptin brain entry via a tanycytic LepR–EGFR shuttle controls lipid metabolism and pancreas function. Nat. Metab. *3*, 1071–1090. https://doi.org/10.1038/s42255-021-00432-5.

80. Frontini, A., Bertolotti, P., Tonello, C., Valerio, A., Nisoli, E., Cinti, S., and Giordano, A. (2008). Leptin-dependent STAT3 phosphorylation in postnatal mouse hypothalamus. Brain Res. *1215*, 105–115. https://doi.org/10.1016/j.brainres.2008.03.078.

81. Bjørbaek, C., Uotani, S., da Silva, B., and Flier, J.S. (1997). Divergent signaling capacities of the long and short isoforms of the leptin receptor. J. Biol. Chem. *272*, 32686–32695. https://doi.org/10.1074/jbc.272.51.32686.

82. Oestreich, A.K., and Moley, K.H. (2017). Developmental and Transmittable Origins of Obesity-Associated Health Disorders. Trends Genet. *33*, 399–407. https://doi.org/10.1016/j.tig.2017.03.008.

83. MacKay, H., Gunasekara, C.J., Yam, K.-Y., Srisai, D., Yalamanchili, H.K., Li, Y., Chen, R., Coarfa, C., and Waterland, R.A. (2022). Sex-specific epigenetic development in the mouse hypothalamic arcuate nucleus pinpoints human genomic regions associated with body mass index. Sci. Adv. *8*, eabo3991. https://doi.org/10.1126/sciadv.abo3991.

84. Saeed, A., McKennan, C., Duan, J., Yang, Y.-N., Kip, K.E., Finegold, D., Vu, M., Swanson, J., Lopez, O.L., Cohen, A., et al. (2025). Mid-life anti-inflammatory metabolites are inversely associated with long-term cardiovascular disease events. EBioMedicine *112*, 105551. https://doi.org/10.1016/j.ebiom.2024.105551.

85. Kaminow, B., Yunusov, D., and Dobin, A. (2021). STARsolo: accurate, fast and versatile mapping/quantification of single-cell and single-nucleus RNA-seq data. Preprint at bioRxiv. https://doi.org/10.1101/2021.05.05.442755.

86. Stoeckius, M., Zheng, S., Houck-Loomis, B., Hao, S., Yeung, B.Z., Mauck, W.M., Smibert, P., and Satija, R. (2018). Cell Hashing with barcoded antibodies enables multiplexing and doublet detection for single cell genomics. Genome Biol. *19*, 224. https://doi.org/10.1186/s13059-018-1603-1.

87. Germain, P.-L., Lun, A., Garcia Meixide, C., Macnair, W., and Robinson, M.D. (2022). Doublet identification in single-cell sequencing data using scDblFinder. F1000Res *10*, 979. https://doi.org/10.12688/f1000research.73600.2.

88. Wolf, F.A., Angerer, P., and Theis, F.J. (2018). SCANPY: Large-scale single-cell gene expression data analysis. Genome Biol. *19*, 15. https://doi.org/10.1186/s13059-017-1382-0.

89. Hao, Y., Hao, S., Andersen-Nissen, E., Mauck, W.M., Zheng, S., Butler, A., Lee, M.J., Wilk, A.J., Darby, C., Zager, M., et al. (2021). Integrated analysis of multimodal single-cell data. Cell *184*, 3573–3587.e29. https://doi.org/10.1016/j.cell.2021.04.048.

90. Bergen, V., Lange, M., Peidli, S., Wolf, F.A., and Theis, F.J. (2020). Generalizing RNA velocity to transient cell states through dynamical modeling. Nat. Biotechnol. *38*, 1408–1414. https://doi.org/10.1038/s41587-020-0591-3.

91. Schindelin, J., Arganda-Carreras, I., Frise, E., Kaynig, V., Longair, M., Pietzsch, T., Preibisch, S., Rueden, C., Saalfeld, S., Schmid, B., et al. (2012). Fiji: An open-source platform for biological-image analysis. Nat. Methods *9*, 676–682. https://doi.org/10.1038/nmeth.2019.

92. Haghverdi, L., Lun, A.T.L., Morgan, M.D., and Marioni, J.C. (2018). Batch effects in single-cell RNA-sequencing data are corrected by matching mutual nearest neighbors. Nat. Biotechnol. *36*, 421–427. https://doi.org/10.1038/nbt.4091.

93. Cao, J., Spielmann, M., Qiu, X., Huang, X., Ibrahim, D.M., Hill, A.J., Zhang, F., Mundlos, S., Christiansen, L., Steemers, F.J., et al. (2019). The single-cell transcriptional landscape of mammalian organogenesis. Nature *566*, 496–502. https://doi.org/10.1038/s41586-019-0969-x.

94. Chen, Y., Chen, L., Lun, A.T.L., Baldoni, P.L., and Smyth, G.K. (2025). edgeR v4: powerful differential analysis of sequencing data with expanded functionality and improved support for small counts and larger datasets. Nucleic Acids Res. *53*, gkaf018. https://doi.org/10.1093/nar/gkaf018.

95. Biancalani, T., Scalia, G., Buffoni, L., Avasthi, R., Lu, Z., Sanger, A., Tokcan, N., Vanderburg, C.R., Segerstolpe, Å., Zhang, M., et al. (2021). Deep learning and alignment of spatially resolved single-cell transcriptomes with Tangram. Nat. Methods *18*, 1352–1362. https://doi.org/10.1038/s41592-021-01264-7.

96. Stringer, C., and Pachitariu, M. (2025). Cellpose3: one-click image restoration for improved cellular segmentation. Nat. Methods *22*, 592–599. https://doi.org/10.1038/s41592-025-02595-5.

97. Nolbrant, S., Heuer, A., Parmar, M., and Kirkeby, A. (2017). Generation of high-purity human ventral midbrain dopaminergic progenitors for in vitro maturation and intracerebral transplantation. Nat. Protoc. *12*, 1962–1979. https://doi.org/10.1038/nprot.2017.078.

98. Sofroniew, N., Lambert, T., Bokota, G., Nunez-Iglesias, J., Sobolewski, P., Sweet, A., Gaifas, L., Evans, K., Burt, A., Doncila Pop, D., et al. (2025). napari: a multi-dimensional image viewer for Python. Zenodo. https://zenodo.org/records/16627702.

99. Hänninen, E., Mueller, A.K., Bagge, J.V., Trujillo, L.S., Fedrizzi, L., Kirkeby, A., and Kajtez, J. (2026). LUMIN: an automated graphical analysis toolbox for high-throughput calcium imaging of in vitro neuronal cultures. Sci. Rep. *16*, 9496. https://doi.org/10.1038/s41598-026-40269-0.

100. Zheng, G.X.Y., Terry, J.M., Belgrader, P., Ryvkin, P., Bent, Z.W., Wilson, R., Ziraldo, S.B., Wheeler, T.D., McDermott, G.P., Zhu, J., et al. (2017). Massively parallel digital transcriptional profiling of single cells. Nat. Commun. *8*, 14049. https://doi.org/10.1038/ncomms14049.

101. Fleming, S.J., Chaffin, M.D., Arduini, A., Akkad, A.D., Banks, E., Marioni, J.C., Philippakis, A.A., Ellinor, P.T., and Babadi, M. (2023). Unsupervised removal of systematic background noise from droplet-based single-cell experiments using CellBender. Nat. Methods *20*, 1323–1335. https://doi.org/10.1038/s41592-023-01943-7.

102. Finucane, H.K., Bulik-Sullivan, B., Gusev, A., Trynka, G., Reshef, Y., Loh, P.R., Anttila, V., Xu, H., Zang, C., Farh, K., et al. (2015). Partitioning heritability by functional annotation using genome-wide association summary statistics. Nat. Genet. *47*, 1228–1235. https://doi.org/10.1038/ng.3404.

## STAR★METHODS

### KEY RESOURCES TABLE

| REAGENT or RESOURCE | SOURCE | IDENTIFIER |
|---|---|---|
| Antibodies | | |
| NKX2-1 (Rabbit) | Abcam | Cat#ab1333737, RRID: AB_2811263 |
| PAX6 (Mouse) | Sigma-Merck | Cat#AMAb91372, RRID: AB_2716656 |
| AGRP (Rabbit) | Phoenix Pharma | Cat#H-003-53 (discontinued), RRID: AB_2313908 |
| AGRP (Goat) | R&D Systems | Cat#AF634, RRID: AB_2273824 |
| aMSH (Sheep) | Millipore | Cat#AB5087, RRID: AB_91683 |
| bMSH (Rabbit | LSBio | Cat# LS-C183969-50 |
| MAP2 (Mouse) | Sigma | Cat#M1406, RRID: AB_477171 |
| TRH (Rabbit) | Thermo Fisher | Cat#PA5-57331, RRID: AB_2648903 |
| TH (Mouse) | Immunostar | Cat#173-22941, RRID: AB_572268 |
| GHRH (Rabbit) | Abcam | Cat#ab18751, RRID: AB_3678595 |
| OTP (Rabbit) | GeneTex | Cat#GTX119601, RRID: AB_11164017 |
| FOXG1 (Rabbit) | Abcam | Cat#ab18259, RRID: 732415 |
| CRH (Rabbit) | Proteintech | Cat#10944-1-AP, RRID: AB_2084279 |
| AQP4 (Rabbit) | Sigma-Merck | Cat#HPA014784, RRID: AB_1844967 |
| hNCAM1 (Mouse) | Santa Cruz Biotechnology | Cat#SC-106, RRID: AB_627128 |
| HuNu (Mouse) | Millipore | Cat#AB1281 |
| NPY (Sheep) | Millipore | Cat#AB1583, RRID: AB_2236176 |
| S100β (Mouse) | Sigma | Cat#S2532, RRID: AB_477499 |
| Somatostatin (Mouse, Alexa Fluor 488 conjugated) | BD Biosciences | Cat#566032, RRID: AB_2739476 |
| NFIA (Rabbit) | Abcam | Cat#ab228897, RRID: AB_2923081 |
| Vimentin (Chicken) | Millipore | Cat#AB5733, RRID: AB_11212377 |
| NR5A1 (Mouse) | Thermo Fisher | Cat#434200, RRID: AB_2532209 |
| POMC (Mouse) | Thermo Fisher | Cat#MA5-38604, RRID: AB_2898516 |
| HuC/D (Mouse) | Invitrogen | A-21271, RRID: AB_221448 |
| Anti-Rabbit Alexa 488 | Jackson ImmunoResearch | Cat#711-545-152, RRID: AB_2313584 |
| Anti-Mouse Alexa 647 | Jackson ImmunoResearch | Cat#711-605-151 |
| Anti-Sheep Cy3 | Jackson ImmunoResearch | Cat#713-165-147, RRID: AB_2315778 |
| Anti-Mouse Cy3 | Jackson ImmunoResearch | Cat#715-165-151, RRID: AB_2315777 |
| Anti-Mouse Alexa 488 | Jackson ImmunoResearch | Cat#715-545-150, RRID: AB_2340846 |
| Anti-Rabbit Cy3 | Jackson ImmunoResearch | Cat#711-165-152, RRID: AB_2307443 |
| Anti-Goat Alexa 488 | Jackson ImmunoResearch | Cat#705-545-147, RRID: AB_2336933 |
| Anti-Sheep Alexa 647 | Jackson ImmunoResearch | Cat#713-605-147, RRID: AB_2340751 |
| Anti-Goat Alexa 647 | Jackson ImmunoResearch | Cat#705-605-147, RRID: AB_2340437 |
| Anti-Mouse Biotinylated | Vector Laboratories | Cat#BA9200 |
| DAPI | Thermo Fisher | Cat#D3571 |
| Hoechst 33342 | Thermo Fisher | Cat#62249 |
| Chemicals, Peptides, and Recombinant Proteins | | |
| Laminin-521 | BioLamina | Cat#LN521-02 |
| Laminin-111 | BioLamina | Cat#LN111-02 |
| iPS-Brew medium | Miltenyi Biotec | Cat#130-104-368 |
| DMEM/F12, GlutaMAX™ | Thermo Fisher/Gibco | Cat#10565018 |
| MACS Neuro Medium | Miltenyi Biotec | Cat#130-093-570 |

(*Continued on next page*)

| *Continued* | | |
|---|---|---|
| REAGENT or RESOURCE | SOURCE | IDENTIFIER |
| N-2 supplement | Thermo Fisher Scientific | Cat#17502048 |
| GlutaMAX | Thermo Fisher Scientific | Cat#35050061 |
| KnockOut™ Serum | Thermo Fisher Scientific | Cat#10828028 |
| Penicillin–Streptomycin | Gibco | Cat#15070063 |
| Accutase | Thermo Fisher Scientific | Cat#A11105-01 |
| EDTA | Thermo Fisher Scientific | Cat#15575020 |
| Y-27632 (ROCK inhibitor) | Miltenyi Biotec | Cat#130-106-538 |
| SB431542 | Miltenyi Biotec | Cat#130-106-543 |
| Noggin | Miltenyi Biotec | Cat#130-103-456 |
| Ascorbic acid | Sigma-Merck | Cat#A4403-100MG |
| BDNF | Miltenyi Biotec | Cat#130-096-286 |
| db-cAMP | Sigma-Merck | Cat#D0627-1G |
| DAPT | Miltenyi Biotec | Cat#130-110-489 |
| GDNF | Miltenyi Biotec | Cat#130-098-449 |
| CHIR99021 (GSK3 inhibitor) | Miltenyi Biotec | Cat#130-106-539 |
| SHH-C24II | Miltenyi Biotec | Cat#130-095-730 |
| IGFBP-3 | R&D Systems | Cat#675-B3-025 |
| BMP4 | Miltenyi Biotec | Cat#130-111-168 |
| BMP7 | R&D Systems | Cat#354-BP-500/CF |
| FGF1 (recombinant human) | Miltenyi Biotec | Cat#130-095-789 |
| CryoStor | StemCell Technologies | Cat#07930 |
| BrainPhys Imaging Optimized Medium | STEMCELL Technologies | Cat#5796 |
| Calbryte 520 AM | AAT Bioquest | Cat#20650 |
| Pluronic F-127 | Sigma Aldrich | Cat#P2443 |
| HBSS+/+ | Gibco | Cat#14025092 |
| SYBR Green Master Mix | Roche | Cat#04887352001 |
| Leptin | R&D systems | Cat#398-LP |
| GLP-1 | Tocris | Cat#5374 |
| HEPES | Thermo Fisher Scientific | Cat#15630049 |
| Ghrelin | BACHEM | Cat#4033077 |
| LEAP2 | Novo Nordisk | Gift from Novo Nordisk A/S. |
| Streptavidin Cy5 Conjugate | Invitrogen | Cat#SA1011 |
| Lectin from Wisteria floribunda | Sigma Aldrich | Cat#L1516 |
| Critical Commercial Assays | | |
| RNAscope Multiplex Fluorescent Kit v2 | ACD Bio-Techne | Cat#323100 |
| RNeasy Plus Micro Kit | QIAGEN | Cat#74034 |
| Maxima First Strand cDNA Synthesis Kit | Thermo Fisher Scientific | Cat#K1642 |
| Evercode WT v3 kit | Parse Biosciences | https://www.parsebiosciences.com/products/evercode-wt/ |
| Chromium Next GEM Single Cell 3' Kit v3.1 | 10x Genomics | Cat#PN-1000268 |
| AGRP Quantikine ELISA kit | R&D Systems | Cat#DAGR00 |
| HCR RNA-FISH (v3.0) probe sets | Molecular Instruments | N/A |
| Oligonucleotides | | |
| qRT-PCR primers | This paper | See Table S1 for sequences |
| TotalSeq-A anti-Nuclear Hashtags (HTO) | BioLegend | Cat#A0451-A0463 |
| TotalSeq™-A anti-human hashtags | BioLegend | Cat#-A0251-A0262 |
| Hs-RAX-C1 | ACD Bio-Techne | Cat#579951 |
| Hs-TBX3-C2 | ACD Bio-Techne | Cat#557441; |
| Hs-PNOC-C2 | ACD Bio-Techne | Cat#1045241 |

(*Continued on next page*)

***Continued***

| REAGENT or RESOURCE | SOURCE | IDENTIFIER |
|---|---|---|
| Opal dyes 480/570/690 | Akoya Bio | Cat#FP1500001KT; #FP1488001KT; #FP1497001KT |
| Chicken ISL1 HCR probe set | Molecular Instruments | NM 205414.1 |
| Chicken SIX6 HCR probe set | Molecular Instruments | NM 204994.1 |
| Chicken POMC HCR probe set | Molecular Instruments | XM_015285103.2 |
| HCR v3.0 amplifiers (B1-Alexa488; B3-Alexa647; B4-Alexa546) | Molecular Instruments | N/A |
| Deposited Data | | |
| Raw and processed ARC protocol and BMP timing sc/snRNA-seq data | This paper | https://doi.org/10.5281/zenodo.20118475 |
| BMI GWAS data | Yengo et al., 2018[61] | https://giant-consortium.web.broadinstitute.org/index.php/GIANT_consortium_data_files |
| Glucose GWAS data | Qiao et al., 2023[59] | https://cnsgenomics.com/data/qiao_et_al_2023_nc/ |
| T2D GWAS data | Mahajan et al., 2018[62] | http://diagram-consortium.org/downloads.html |
| Height GWAS data | Yengo et al., 2018[61] | https://giant-consortium.web.broadinstitute.org/index.php/GIANT_consortium_data_files |
| Experimental Models: Organisms/Strains | | |
| Rat: Hsd:RH-Foxn1rnu (athymic nude rat) | Inotiv (Prev. Envigo) | N/A |
| Chicken: Bovan Brown embryos | Medeggs Ltd | N/A |
| Experimental Models: Cell Lines | | |
| RC17 hESC line | Roslin Cells | hPSCreg: RCe021-A, RRID: CVCL_L206 |
| KOLF2.1J hiPSC line | hPSCreg | WTSIi018-B-129 |
| BIONi010 hiPSC line | hPSCreg | BIONi010-C-52, RRID: CVCL_C9MC |
| SCTi003-A hiPSC line | STEMCELL Technologies | SCTi003-A, RRID: CVCL_C1W7 |
| Software and Algorithms | | |
| Cell Ranger v7.1.0 | 10x Genomics | https://www.10xgenomics.com/support/software/cell-ranger |
| STARsolo v2.7.10b | Kaminow et al.[85] | https://github.com/alexdobin/STAR?tab=readme-ov-file |
| CITE-seq-Count v1.45 | Stoeckius et al.[86] | https://hoohm.github.io/CITE-seq-Count/ |
| COMMUNEQAID pipeline | CBMR Single-Cell Omics Platform | https://github.com/CBMR-Single-Cell-Omics-Platform/COMUNEQAID |
| Parse Biosciences Pipeline v1.4.1 | Parse Biosciences | https://www.parsebiosciences.com/ |
| scDblFinder v1.16.0 | Germain et al., 2022[87] | https://www.bioconductor.org/packages/release/bioc/html/scDblFinder.html |
| Scanpy v1.9.5 | Wolf et al.[88] | https://scanpy.readthedocs.io/en/latest/index.html |
| Seurat v4.3.0 or v5.3.1 | Hao et al.[89] | https://satijalab.org/seurat/ |
| scVelo v0.3.2 | Bergen et al.[90] | https://scvelo.readthedocs.io |
| Meta[91]Neighbor v1.22.0 | Crow et al.[50] | https://www.bioconductor.org/packages/release/bioc/html/MetaNeighbor.html |
| FastMNN batchelor v1.18.1 | Haghverdi et al.[92] | https://www.bioconductor.org/packages/release/bioc/html/batchelor.html |
| Monocle3 v1.3.7 | Cao et al.[93] | https://www.bioconductor.org/packages/release/bioc/html/monocle.html |
| Custom Python and R scripts | This paper | https://github.com/kirkebylab/In_vitro_ARC_VMH_analysis |

(*Continued on next page*)

| Continued | | |
|---|---|---|
| REAGENT or RESOURCE | SOURCE | IDENTIFIER |
| edgeR v4.0.16 | Chen et al., 2024[94] | https://bioconductor.org/packages/release/bioc/html/edgeR.html |
| Tangram v1.0.4 | Biancalani et al.[95] | https://tangram-sc.readthedocs.io/en/latest/# |
| CELLECT v1.3.0 | Timshel et al.[58] | https://github.com/perslab/CELLECT |
| Cellpose v3.1.1.1 | Stringer and Pachitariu[96] | https://cellpose.readthedocs.io/en/v3.1.1.1/ |
| ImageJ/FIJI v2.1.0 | Schindelin et al., 2012[91] | https://fiji.sc |
| GraphPad Prism v10.2.3 | GraphPad Software | https://www.graphpad.com |
| Adobe Photoshop 2023/2024 | Adobe | https://www.adobe.com/products/photoshop.html |
| Other/Equipment | | |
| Cryostat | Leica Microsystems | Model: CM1950; RRID: |
| Confocal microscope | Leica Microsystems | Model: Stellaris 5; RRID: |
| Confocal microscope | Carl Zeiss | Model: LSM880; RRID: |
| Spinning disk confocal microscope | Nikon / Yokogawa | Model: ECLIPSE Ti2-E with CSU-W1; RRID: |
| Cell sorter | Sony Biotechnologies | Model: SH800S; RRID: |
| High-throughput confocal system | Yokogawa | Model: CV8000; RRID: |
| Sequencing system | Illumina | Model: NovaSeq 6000; RRID: |
| Absorbance microplate reader | Molecular Devices | SpectraMAX iD3 |

## EXPERIMENTAL MODEL AND STUDY PARTICIPANT DETAILS

### Human Pluripotent Stem Cell Lines

Four human pluripotent stem cell lines were used: One hESC, RC17 (Roslin Cells; hPSCreg RCe021-A; sex: female), and 3 human induced pluripotent stem cell lines (hiPSC), KOLF2.1J (hPSCreg WTSIi018-B-129; sex: male), BIONi010 (hPSCreg BIONi010-C-52; sex: male) and SCTi003-A (hPSCreg SCTi003-A; sex: female). All lines were registered in hPSCreg and maintained per supplier guidance. All hPSC lines were obtained from authenticated repositories (hPSCreg/Roslin/Bioneer) with reference identity documentation. No additional short tandem repeat (STR) profiling was performed in this study. Cultures were maintained at 37°C for a maximum of 50 passages and routinely screened for mycoplasma every three months by Eurofins using a standardized qPCR assay.

### Athymic nude rats

Seven female adult (>12 weeks old at beginning of study) athymic nude rats (Hsd:RH-Foxn1rnu (Derived from animals obtained from the Rowett Research Institute, Aberdeen, Scotland) β; Inotiv/Envigo, The Netherlands n = 7, weighing >225 g at the time of surgery were used for unilateral intrastriatal xenotransplantation. All animals were healthy at baseline and had no previous procedures prior to transplantation. Rats were group-housed under controlled temperature and humidity and maintained on a 12:12-hr light:dark cycle with ad libitum access to standard chow and water. Cages contained standard bedding with nesting material, a shelter, and environmental enrichment (wooden stick and paper tunnel). Animals underwent surgery in ID order without blinding, as all received the same cell type. One animal was excluded from analysis because it was sacrificed early due to health issues, which prevented the graft from reaching the same maturation stage as in animals sacrificed later.

All procedures were carried out in agreement with the European Union Directive 2010/63/EU, the ARRIVE (Animal Research: Reporting In Vivo Experiments) guidelines and were approved by the local ethical committee at Lund University and the Swedish Department for Agriculture (Jordbruksverket) (5.2.18-10992/18).

### Avian Embryos

Fertilized Gallus gallus domesticus (Bovan Brown) eggs were obtained from Medeggs Ltd, Norfolk, UK. Embryos were staged according to Hamburger and Hamilton (Hamburger and Hamilton, 1992), and HH17 and HH20 embryos were harvested for Hybridization chain reaction (HCR) analysis. Sex was not determined as the studies were performed on embryos prior to sexual differentiation. Seven embryos were analyzed at each time point. All studies were conducted according to the UK Animals (Scientific Procedures) Act 1986/EU Directive 2010/63/EU, and were approved by the University of Sheffield Local Ethical Review committee. Ethical approval or Home Office licensing was not required for egg work, as eggs were not incubated beyond HH20 (hatching is E21). Named Animal Care and Welfare Officers (NACWOs) had oversight of all incubated eggs.

## METHOD DETAILS

### Ethics declaration

This study uses previously derived hESC line RC17 (hPSCreg RCe021-A) and hiPSC lines KOLF2.1 (hPSCreg WTSIi018-B-129), SCTi003-A (hPSCreg SCTi003-A) and BIONi010 (hPSCreg BIONi010-C-52). All cell lines have been derived under local ethical approval at the original derivation sites and with informed consent from donors. No new hESC or hiPSC lines were derived in this study. The Kirkeby lab holds a relevant ethical approval to work with hPSC lines (H-21043866). Xenotransplantation studies to rats were approved by a local animal research committee and performed under animal ethics license 2022-15-0201-01236.

### Cell culture and neuronal differentiation

Human pluripotent stem cells (hPSC) were maintained and differentiated as previously described with modifications. One hESC line (RC17; Roslin Cells, hPSCreg: RCe021-A) and three hiPSC lines (KOLF2.1J hPSCreg: WTSIi018-B-129; BIONi010; hPSCreg: BIONi010-C-52; SCTi003-A hPSCreg: SCTi003-A) were used in this study. Pluripotent cells were cultured in iPS-Brew medium (Miltenyi Biotec, #130-104-368) on plates coated with 1 μg/cm$^2$ Laminin-521 (Biolamina, #LN521-02) in PBS containing $Mg^{2+}$ and $Ca^{2+}$ (PBS$^{+/+}$). Cells were passaged at 70–90% confluency using 0.5 mM EDTA (75 μL/cm$^2$; Thermo Fisher Scientific, #15575020) for 5–7 min, then dissociated and replated in fresh iPS-Brew supplemented with 10 μM ROCK inhibitor Y-27632 (Miltenyi, #130-106-538) for 24 h. Medium was changed daily thereafter.

Differentiation into regionalized hypothalamic progenitors was based on a protocol adapted from Nolbrant et al.[97] From day 0 (d0) to d11, cells received N2 medium consisting of 50% MACS Neuro Medium and 50% DMEM/F12, supplemented with 1% N-2 (Thermo Fisher Scientific, #17502048), 1% GlutaMAX (Thermo Fisher Scientific, #35050061), and 10 U/mL penicillin/streptomycin (Gibco, #15070063). From d11 onwards, cells were cultured in NB21 medium (MACS Neuro Medium with 2% NB-21 supplement, 1% GlutaMAX, and penicillin/streptomycin). Medium was replaced every 2–3 days. ROCK inhibitor (10 μM) was included following each replating. At d0, hPSC were dissociated as above, washed in DMEM/F12 with 5% KOSR (Gibco, #10828-028), and plated at 10,000 cells/cm$^2$ on plates coated with 2 μg/cm$^2$ Laminin-111 (Biolamina, #LN111-02) in PBS$^{+/+}$. At d11, progenitors were dissociated using Accutase (Thermo Fisher Scientific, #A11105-01) for 10 min and replated at 800,000 cells/cm$^2$ onto Laminin-111–coated plates. At d16, cells were transferred to 2 μg/cm$^2$ Laminin-521–coated plates at a density of 700,000 cells/cm$^2$. All differentiations included 10 μM SB431542 (Miltenyi, #130-106-543) and 100 ng/mL Noggin (Miltenyi, #130-103-456) from d0–9, 0.2 mM ascorbic acid (Sigma-Merck, #A4403-100MG) and 20 μg/mL BDNF (Miltenyi, #130-096-286) from d11, and 500 μM dibutyryl-cAMP (Sigma-Merck, #D0627-1G), 1 μM DAPT (Miltenyi, #130-110-489), and 10 ng/mL GDNF (Miltenyi, #130-098-449) from d16. From d16 onward, this combination of factors (AA, BDNF, db-cAMP, DAPT, GDNF) in NB21 medium was referred to as maturation medium (MM).

ARC differentiation included 0.3 μM CHIR from d2–9, 300 ng/mL SHH from d0–9, and 400 ng/mL IGFBP-3 (R&D Systems, #675-B3-025) from d7–14. BMP4 (50 ng/mL; Miltenyi, #130-111-168) was supplemented at defined time points, and BMP7 (100 ng/mL; R&D Systems, #354-BP-500/CF) was added from d25–50 of maturation in ARC cultures. For 3D spheroid generation, day 16 patterned hypothalamic progenitors were counted, pelleted at 400 × g for 5 min, and resuspended at 250,000 cells/mL in NB21 medium supplemented with 20 ng/mL BDNF, 0.2 mM ascorbic acid, 500 μM db-cAMP, and 1 μM DAPT. 50,000 cells were transferred per well into ultra-low attachment U-bottom 96-well plates (Costar), centrifuged at 100 × g for 5 min, and cultured at 37°C with 5% $CO_2$. 75% of the maturation medium was exchanged every 2–3 days until the endpoint of the experiment.

### Cryopreservation and thawing of progenitors

Day 16 hypothalamic progenitors were dissociated with accutase, resuspended in wash medium, counted, and pelleted. Cells were resuspended in ice-cold CryoStor (StemCell Technologies, Cat#07930) with 10% DMEM/F12, GlutaMAX™ (Thermo Fisher/Gibco; Cat#10565018) and placed in a pre-chilled CoolCell for controlled-rate freezing overnight at −80°C (~1°C/min), then transferred to −140°C liquid nitrogen for long-term storage. For thawing, vials were warmed in a 37°C water bath until a small ice crystal remained, diluted in warm wash medium and centrifuged (400 xg, 5 min). Pellets were resuspended in associated medium with 10 μM ROCK inhibitor and plated onto Laminin-coated plates.

### Xenotransplantation of d16 ARC progenitors

All procedures were conducted in accordance with the European Union Directive (2010/63/EU) and had approval by the local ethical committee at Lund University and the Swedish Department for Agriculture (Jordbruksverket). Seven adult, female, athymic nude rats (Hsd:RH-Foxn1$^{rnu}$, Inotiv (Prev. Envigo), Indiana, USA) were housed on a 12:12-hr light:dark cycle with *ad libitum* access to food and water. ARC cells were prepared for transplantation by thawing day 16 cryopreserved progenitors in wash buffer (0.5% human serum albumin (HSA) in HBSS-/-) and centrifuged at 400 xg at RT. Cells were reconstituted to 167,000 cells/μL in Neurobasal (Thermo Fisher Scientific, #21103049) with 20 U/mL pulmozyme, 1x B27 supplement (Thermo Fisher Scientific, #17504044), 10 μM ROCKi, 0.2 mM ascorbic acid and 20 ng/mL BDNF. We performed transplantations to the striatum due to its surgical accessibility, large volume and absence of endogenous peptidergic neurons, minimizing confoundment of the post-transplantation graft analysis. For unilateral intrastriatal xenotransplantations, the rats (>225 g) underwent general anesthesia (Fentanyl (45 mg kg$^{-1}$)-Domitor (0.03 mg kg$^{-1}$) mix, Apoteksbolaget, i.p.). A glass capillary was inserted into the striatum at the following coordinates for two deposits: $AP_1$: +0.9, $ML_1$: -3.0, $DV_1$: -5.0; $AP_2$: +1.4, $ML_2$: -2.6, $DV_2$: -5.0. The cells were infused in a volume of 0.1 μL per 12 s over 3 min (1.5 μL of 250,000 cells

in total) followed by 2 min diffusion time per deposit. Of the seven transplanted animals, one was sacrificed early due to illness and therefore excluded from the study. Of the remaining animals, 4 were perfused for IHC and brains from 2 animals were snapfrozen for RNAscope. Graft presence was confirmed in all these six animals through human probe RNAscope and staining for human neural cell adhesion molecule (hNCAM).

## RNA Extraction and qRT-PCR

Approximately 500,000 cells or 8-12 spheroids were lysed in 350 μL RLT buffer (QIAGEN, #74034) containing 0.5 mM β-mercaptoethanol (Thermo Fisher Scientific, #31350010), snap frozen, and stored at −80°C. Total RNA was isolated using a QIAGEN QIAcube (SN 48555) instrument and the RNeasy Plus Micro Kit (QIAGEN, #74034). cDNA synthesis was performed with 1 μg RNA using the Maxima First Strand cDNA Synthesis Kit (Thermo Fisher Scientific, #K1642), diluted in 250 μL EB buffer (QIAGEN, #19086), and stored at −20°C. qRT-PCR reactions were prepared using SYBR Green Master Mix (Roche, #04887352001), gene-specific primers (see Table S1), and sample cDNA before being dispensed using an iDOT liquid handler. qRT-PCRs were run on a LightCycler 480 II system (Roche, #05015243001) with 40 cycles: 60°C for 60 s (annealing/extension), and 95°C for 30 s (denaturation). Mean CT values of technical duplicates were used for relative quantification, normalized to the average expression in undifferentiated hPSC. Target genes were normalized to both *GAPDH* and *ACTB*, and fluorescence intensity values were plotted using GraphPad Prism v10.2.3.

## Expression matrix analysis from qPCR data

qRT-PCR expression data from early differentiations were log-normalized prior to principal component analysis. To identify the effects of distinct factors on PC1 embedding, a linear model was constructed to predict PC1 embedding values, including all tested compounds. The resulting model was analyzed using estimated marginal means (EMMs) to identify the average effects of different factors while adjusting for other covariates in the model. To identify genes in early differentiations which are predictive of marker genes in late differentiations, a linear model was constructed to test the relationship between each individual gene and the selected late marker gene. Gene expression values were log normalized and scaled prior to analysis. Only differentiations in which the two genes had been measured at the early and late timepoints were included.

## Immunocytochemistry (ICC)

All 2D cultured cells were fixed in 4% paraformaldehyde for 20 min followed by three washes with phosphate-buffered saline (PBS-/-) and stored at 4°C until staining. Cells were blocked for 1-3 h at room temperature (RT) in blocking buffer (0.1%, Triton X-100 and 5% (vol/vol) donkey serum in PBS-/-). Primary antibodies (see Table S2) were diluted in blocking buffer and incubated at 4°C overnight. Following incubation, cells were washed three times with PBS-/- before incubation with secondary conjugated fluorophores (1:200 or 1:500, see Table S3) and 2 μg/mL DAPI for 2h at RT on a shaker. A final three PBS washes were performed before imaging.

Spheroids were fixed with 4% PFA for 1 hour at RT, washed twice with PBS and blocked for 3-6 h at 4°C with blocking buffer. Then they were incubated with primary antibodies diluted in blocking buffer (200μL/tube) on a shaker at 4°C for 48 h. The primary antibody solution was removed and the samples were washed with 300 μL blocking buffer, once for 2 min and then for 6 h at 4°C. All subsequent steps were performed under the exclusion of light. The secondary antibodies and 2 μg/mL DAPI for nuclear counterstaining were diluted in 200 μL blocking buffer and added to the tissues. The samples were again incubated on a shaker at 4°C for 48 h. Subsequently, the tissues were washed for 2 min and then at 4°C for 6 h on a shaker. After two more washes in PBS, the tissues were transferred to 18-well ibidi chambers (Ibidi, #81816) or 96-well microplates (PhenoPlate, #6055300) and stored at 4°C until visualization.

### *ICC - Wisteria floribunda agglutinin (WFA)*

Spheroids fixed with 4% PFA were incubated with blocking buffer for 1 h at room temperature. Then they were incubated with Lectin from *Wisteria floribunda* (Sigma-Aldrich, Cat#L1516) diluted in blocking buffer at 1:500 (200μL/tube) on a shaker at room temperature for 3h. The solution was removed and the samples were washed with 300 μL blocking buffer, once for 5 min. The spheroids were incubated with Streptavidin Cy5 (Invitrogen, Cat#SA1011) at 1:1000 for 1h at room temperature on a shaker. All subsequent steps were performed under the exclusion of light. Subsequently, the tissues were washed for 1h on a shaker. The spheroids were transferred to 96-well microplates (PhenoPlate, #6055300) and stored at 4°C until visualization.

### *ICC - 3D quantifications*

Quantification of AGRP and POMC (αMSH) positive neurons of day 50 ARC 3D spheroids was performed partially manually and partially using the Imaris image analysis software (Oxford Instruments). Firstly, images of the stained spheres were taken using the Nikon inverted confocal microscope (ECLIPSE Ti2-E, Nikon with CFI Plan Apochromat Lambda 20X/0.75, WD 1.0mm) equipped with a spinning disk module (CSU-W1, Yokogawa). From each sphere, exactly 61 slices with 1μM distance starting from the top of the tissue were included in the analysis. Imaris was used to quantify the number of DAPI positive cells. The number of AGRP and αMSH positive cells were counted manually using ImageJ (FIJI[91]) by three independent people, the values were averaged. Then the ratio of positive neurons to DAPI+ cells was calculated and plotted.

## Multiplexed immunohistochemistry

The nude rats were anaesthetized with Sodium Pentobarbital (250-350 mg/kg, 1.4 mL of 60 mg/mL per rat, Apotek, Sweden) and transcardially perfused with 0.1 M phosphate buffer. The brains were removed, post-fixed in 4% PFA for 24 h at RT and immersed

in 25% sucrose solution until fully dehydrated. For sectioning, the brains were cut into 35 μM coronal sections using a cryostat (Leica Microsystems, CM1950 cryostat) and systematically sampled in series of eight. The sections were stored in cryoprotectant at -20°C until use. Sections encompassing the striatum (site of xenotransplant), and the ARC/PVH (endogenous positive controls) were used for immunostainings.

For immunostainings, the sections were initially rinsed in PBS-/- and subjected to antigen retrieval in pre-heated (80°C) TRIS-EDTA buffer (10 mM Tris Base, 1 mM EDTA Solution, 0.05% Tween-20, pH 9) for 30 min and left to cool to RT before rinsing in PBS-/-. For SG chromogenic staining, endogenous peroxidase activity was blocked by incubation in 1% hydrogen peroxidase ($H_2O_2$) in PBS-/- for 20 min at RT, prior to pre-incubation in blocking buffer (5% donkey or goat serum, 1% bovine serum albumin, 0.3% Triton X-100 (TX) (0.1% TX for SG chromogenic staining) in PBS-/-) for 30 min at RT. Next, the sections were incubated overnight (2 h for anti-hNCAM) at RT in primary antibodies diluted in blocking buffer. After primary antibody incubation, the sections were rinsed in washing buffer (0.25% bovine serum albumin, 0.1% Triton X-100 in PBS) or in 0.1% TX-PBS for SG chromogenic staining, followed by incubation for 1 h at RT in fluorophore-conjugated or biotin-coupled secondary antibodies diluted in washing buffer. See Tables S2 and S3 for information on primary and secondary antibodies, respectively.

For immunofluorescence, the sections were thereafter rinsed once in washing buffer and twice in PBS-/-, mounted on glass slides, and coverslipped using FluorSave™ (Millipore, #345789). For SG chromogenic staining, the sections were rinsed in 0.1% TX-PBS and incubated for 1 h at RT in avidin-biotin complex solution (Vector Laboratories, #AK-5000) diluted in TX-PBS according to manufacturer's instructions. After incubation, the sections were rinsed once in TX-PBS and twice in PBS-/- before color development for 10 min in ImmPACT Vector SG Substrate chromogen solution (Vector Laboratories, #AK-4705) without $H_2O_2$, and for additional 2 min with $H_2O_2$. The sections were next mounted on gelatin-coated microscope glass slides, dehydrated in a series of ethanol (70%, 96%, 99%), cleared in xylene, immediately cover slipped using DPX New mountant (Merck, #1.00579), and left to dry overnight.

## ISH on cell cultures

Cells were fixed for 30 min at RT. *In situ* hybridization was performed using the RNAscope® Multiplex Fluorescent Kit v2 (ACD BioTechne, #323100) according to manufacturer's instructions for cultured adherent cells in 96-well plates. The probes and fluorophores used in this study can be found in Table S4. For combinatory ICC/ISH, the standard ICC protocol was performed after the RNAscope protocol.

## ISH/IF on brain sections

Rats were deeply anaesthetized with $CO_2$ gas and sacrificed by decapitation. The brain was quickly removed and immediately immersed in dry ice and stored at -80°C until sectioning. The brains were sectioned into 12 μM coronal sections using a cryostat (Leica, CM1950 cryostat) and directly mounted onto SuperFrost® Plus glass slides (Thermo Scientific, #J1800AMNZ) and stored at -80°C. ISH was performed according to the RNAscope® Multiplex Fluorescent v2 Assay combined with Immunofluorescence (Doc. No. MK51-150 Rev B/Appendix C-D) with several changes. The sections were first fixed in pre-chilled 4% PFA at 4°C overnight prior to dehydration and incubation in hydrogen peroxide (ACD Bio-Techne, #322330 or 3% $H_2O_2$ in RNAse-free water). Next, the sections were subjected to co-detection target retrieval (#323166, ACD Bio-Techne, Milan, Italy) for 5 min at 98-100°C, rinsed in dd$H_2O$, and transferred to 100% ethanol for 3 min. The sections were then incubated in primary antibodies at 4°C overnight. According to protocol, the sections were rinsed post-fixed and subjected to protease treatment (RNAscope® Protease Plus (ACD Bio-Techne, #322330), 1:5 diluted in RNAse-free water) for 15 min at 40°C. Next, RNA-specific oligonucleotide target probes were hybridized, amplified, and developed using Opal Dye fluorophore 570. Following the RNAscope protocol, the sections were rinsed and incubated in secondary antibodies for 60 min at RT. Lastly, the sections were rinsed and coverslipped using VectaShield Vibrance antifade Mounting medium with DAPI (#H-1800, Vector Laboratories, CA, USA). See Tables S2–S4 for information on primary and secondary antibodies, probes and fluorophores.

## Chick collection and HCR

Fertilized Bovan Brown eggs (Henry Stewart & Co., Norfolk, UK) were used for all experiments. Eggs were incubated and staged according to the Hamburger-Hamilton chick staging system (Hamburger and Hamilton, 1992). Briefly, fertilized eggs were stored at 14°C, then incubated in a humidified incubator at 37°C. Fertilized eggs were incubated for 28–29 h to obtain HH6, 30–32 h to obtain HH8 (3–5 somite embryos), 40–42 h to obtain HH10 embryos (9–12 somites), 54–60 h to obtain HH 13–15 h, 3 days for HH17/18 and 3.5 days for HH20/21.

Hamburger & Hamilton stage 8–20 embryos were harvested and fixed in 4% Paraformaldehyde. HCR v3.0 was performed on embryos and cryosections using reagents and modified protocol from Molecular Instruments, Inc. For wholemount HCR, the fixed embryos were dehydrated in a series of methanol and stored at −20°C. The samples were then rehydrated and washed in PBS + 0.1% Tween (PBST). The samples were treated with Proteinase K (10 μg/mL) for 2–3 min, fixed for 20 min and then washed first with PBST, then with 5xSSC. Samples were then preincubated with a hybridization buffer for 30 min. The probe pairs (4–10nM) were then added and incubated at 37°C overnight. See Table S4 for information on probes used. For HCR on cryosection, the post-fixed slides were treated with acetylation mix (11.2 μL of Triethanolamine +2.5 μL of acetic anhydride per ml) for 10 min, washed with PBST, fixed again for 10 min and then washed first with PBST, then with 5xSSC.and preincubated with a hybridization buffer for 30 min and the probe pairs (4–10 nM) were added and incubated at 37°C overnight. The next day, samples were washed 4 times in the probe wash buffer, then 2 times in the 5 SSC buffer, and then preincubated in Amplification buffer for 5 min. Even and odd hairpins for each of the genes

were snap-cooled by heating at 95°C for 90 s and cooling to RT for 30 min. The hairpins were then added to the amplification buffer and added to the samples and incubated overnight at RT in dark. Samples were then washed in 5x SSC and DAPI was added as a counterstaining.

### Imaging and processing

Imaging of ICC of *in vitro* derived cells was performed on the Leica AF600 widefield epifluorescence microscope (Plan-Fluotar 20x/0.40 Dry) and for the main figures, the confocal microscope Leica Stellaris 5 (Plan-apochromat 40x/1.25 GLYC). All imaging of 3D tissue staining was performed using either the Zeiss LSM880 (EC Plan-Neofluar 20x0.50 WD 2.0mm and C-Plan-Apochromat 63x1.40 Oil UV-IR WD 0.14mm) or an inverted confocal microscope (ECLIPSE Ti2-E, Nikon with CFI Plan Apochromat Lambda 20X/0.75, WD 1.0mm) equipped with a spinning disk module (CSU-W1, Yokogawa). Images were processed using ImageJ (version 2.1.0). Images of xenotransplantation sections (single and stacks) were acquired by confocal fluorescence (Leica Stellaris 5, Plan-Fluotar 10x/0.30 Dry or Plan-apochromat 40x/1.25 GLYC), widefield fluorescence (AF6000, Plan-Fluotar 10x/0.30 Dry or Plan-Fluotar 20x/0.40 Dry) or widefield brightfield microscopy (Leica DM5500, plan-apochromatic 10x/0.40, DFC450 color camera). The images were processed using ImageJ and brightness/contrast were adjusted using Adobe Photoshop 2024. Chicken embryos were imaged using Nikon W1 Spinning Disk Confocal microscope with Nikon software. Images were processed and digitally aligned using ImageJ (FIJI) and Adobe Photoshop 2023.

### AGRP ELISA

To measure the concentration of secreted AGRP in the media of developing ARC neurons, we used the AGRP Quantikine ELISA kit (R&D Systems, #DAGR00), which employs a solid phase sandwich ELISA that contains Sf 21- expressed recombinant human AGRP as the standard calibrator. This assay has previously been shown to quantitate the recombinant factor with an assay range of 7.5-500 pg/mL. For basal secretion, cell culture supernatants were collected every 7 days from day 30 to 70 *in vitro* between 48-72h post media change. For stimulations, 3-6 spheres were pooled in MACS Neuro Medium with NB21 and ascorbic acid. After a 1-hour baseline incubation, the spheroids were stimulated with vehicle, ghrelin (100 nM), LEAP2 (2 μM) or ghrelin and LEAP2 for 4 h. Medium was collected after baseline and stimulation. Optical density was measured in the SpectraMAX iD3 (Molecular Devices), and the data were processed per the manufacturer's guidelines. For the stimulation experiment, each condition was normalized to its baseline.

### FGF1 stimulation experiment

To assess transcriptional responses to fibroblast growth factor 1 (FGF1), day 50 and day 70 ARC cultures were stimulated for 30 minutes with recombinant human FGF1 (Miltenyi, #130-095-789). Accutase was pre-warmed at 37°C for 10 minutes prior to use. Half of the culture medium was removed from each well and replaced with fresh medium containing FGF1 to a final concentration of 50 ng/mL. Control wells received vehicle medium without FGF1.

Following the 30-minute stimulation, cells were washed once with PBS-/- and incubated with 150 μL of warm Accutase at 37°C for 5 minutes. Cells were then collected in ice-cold NB21 medium and kept on ice before centrifugation at 700 × g for 10 minutes. The supernatant was discarded, and the cell pellets were snap frozen on dry ice and stored at −80°C until downstream single-nucleus RNA sequencing (snRNA-seq) analysis.

### Calcium Imaging

Two days before the start of the experiment, the medium of cells cultured in 96-well microplates (PhenoPlate, #6055300) was replaced with BrainPhys Imaging Optimized Medium (STEMCELL Technologies, #5796) supplemented with NB21 and maturation factors (AA, BDNF, db-cAMP, DAPT, GDNF). The day of the experiment, cells were incubated for 45 min at 37°C with 3 μM Calbryte 520 AM calcium indicator (AAT Bioquest, #20650) in BrainPhys Imaging Optimized Medium with 0.02% Pluronic F-127 (Sigma Aldrich, #P2443). Cells were then washed with PBS-/- and left in Hanks' Balanced Salt Solution (HBSS+/+, Gibco #14025092) before being transferred to a high-throughput spinning disk confocal microscope (CV8000, YOKOGAWA) equipped with a pipetting robot and an environmental chamber. The 20x water immersion objective was used for imaging. For the functional characterization of ARC cultures, cells were continuously imaged at 2 Hz for a total of 240 seconds. Baseline images were acquired first and after 20 seconds, leptin (100 ng/mL), GLP-1 (100 nM) or vehicle was added. The corresponding response was recorded for 210 seconds, and then KCl was added to reach a final concentration of 15 mM. To correlate calcium response with cellular identity, cultures were prepared as described in the ICC methods chapter and imaged using the same microscope as for calcium recordings.

### Calcium imaging analysis

Regions of interest were selected manually using Napari (v.0.4.19.post1)[98] or deep learning-based Cellpose (v. 3.1.1.1)[96] segmentation through LUMIN's segmentation pipeline.[99] Fluorescence intensity was extracted for each cell in each frame. The data was $\Delta F/F0$ normalized using the pre-stimulation recording as baseline. Cells unresponsive to KCl were considered non-viable and excluded from the downstream analysis. The response was quantified at a single-cell level using a 100s analysis window starting 30s after stimulation for 100 ng/mL leptin and a 10s analysis window starting 60s after stimulation for 100 nM GLP-1. The area under the curve (AUC) was calculated for each cell, and a cell was classified as responding if its AUC was 2 standard deviations above the mean AUC of the control response. For the ICC correlation analysis, fluorescence ICC images of Hoechst, Calbryte, and the marker channels of interest were merged into a multi-channel image. Cells positive for the marker were manually identified from the corresponding

calcium recording using built-in Napari functionality. The fluorescence intensity values were extracted, traces were normalized and AUC was computed as described above.

### Single-cell/nucleus isolation for sequencing

For the single-cell sequencing experiment, d16 ARC progenitors were thawed in warmed wash media (DMEM/F12 + 5%KOSR). After counting, cells were centrifuged at 500xg for 5 min at 4°C before resuspension at 5 mio cells/mL in staining buffer (PBS-/- with 0.5% BSA). Cells were stained with 0.5 µg of unique TotalSeqTM-A anti-Nuclear Hashtags (HTO) (Biolegend) and incubated for 30 min at 4°C. After antibody tagging, cells were spun down at 500xg for 5 min at 4°C and wash was repeated three times with staining buffer before a final resuspension at 1000 cells/µL.

For single-nuclei sequencing, d16 cryopreserved progenitors or snap-frozen pellets of d50/70/80 ARC cultures were used. The nuclei were thawed on ice for 2 min and lysed with EZ Lysis Buffer (Nuclei EZ Prep nuclei isolation kit, Sigma, #NUC101-1KT). The lysed sample was incubated on ice for 5 min, before filtering through a 40 µm cell strainer (PluriSelect, #43-10040-40) into a fresh 2mL Protein LoBind tube (Sigma, #EP0030108132). Filtered nuclei were centrifuged for 5 min at 500xg before resuspension in nuclei buffer (PBS-/- with 1% BSA, 2.5 mM MgCl (Sigma, #M1028), 0.2U/µL RNase inhibitor (Sigma, #3335399001). For 10X sequenced samples, the nuclei were incubated together with unique TotalSeqTM-A anti-Nuclear Hashtags (HTO) for multiplexing for 30 min. The hashtagged nuclei from each batch were washed twice with Nuclei Buffer and stained with 7-AAD (ThermoFisher Scientific, #00-6993-50).

### 10X barcoding for sc/sn-RNAseq

Single-cell samples were FACS sorted (SONY SH800S cell sorter) with a 70µm sorting chip (Sony Biotechnologies, cat. no.: LE-C3207) and pooled at equal ratios for one 10X lane using the 10X v3.1 chemistry kit. The nuclei samples were also FACS sorted into a 2mL Protein LoBind tube with 18.8µL RT Reagent B (10X Genomics, Chromium Next GEM Single Cell 3' Kit v3.1, cat. no.: PN-1000268). Following sorting, the volume was adjusted to 43.1µL with Nuclei Buffer and the final GEM Master Mix reagents were added as per manufactures procedure, which was followed from then on for library preparation with dual indexing. Each sample was divided into three 10X lanes.

HTO libraries were prepared by following the procedure from BioLegend. In short, for generating GEM cDNA, the reaction was added 1 µL of 0.2µM HTO primer (5'-GTGACTGGAGTTCAGACGTGTGC*T*C) prior to PCR cycling and cleaned up using SPRISelect magnetic beads (Beckman Coulter, #B23318), 80% ethanol, and eluted with Buffer EB (Qiagen, #1014609). The concentration was determined with Qubit (Thermo Fisher Scientific) and Qubit dsDNA HS Assay Kit (Thermo Fisher Scientific, #Q32854). Libraries reactions were generated with the following mixture: 20ng HTO cDNA, 2.5µL 10µM SI PCR primer (5'-AATG ATACGGCGACCACCGAGATCTACACTCTTTCCCTACACGACGC*T*C), 2.5µL 10µM TruSeq D7XX_s (5'- CAAGCAGAAGACG GCATACGAGAT[8X]GTGACTGGAGTTCAGACGTGT*G*C), 50µL KAPA HiFi HotStart ReadyMix (Roche, #KK2602), nuclease free water to 100µL. The following PCR reaction was used: 98°C, 2 min; 15x[98°C, 20 sec; 64°C, 30 sec; 72°C, 20 sec], 72°C, 5 min. Cleanup and quantification was as above. The libraries were quantified by Qubit and TapeStation (Agilent TapeStation 4200 System) with TapeStation High Sensitivity D1000 DNA (Agilent, #5067-5584 and #5067-5585).

All 10X sc/snRNA samples were sequenced on SP/S2 flow cells (Illumina) on NovaSeq6000. All libraries were sequenced using recommended settings aiming for a depth of ~50k reads per cell (28bp R1; 10bp I1; 10bp I2; 90bp R2).

### Parse barcoding for snRNA-seq

After nuclei isolation, nuclei were counted and fixed using the EvercodeTM Nuclei Fixation v3 kit (Parse Biosciences) before cryopreserving and storing them at -80°C. Thawed nuclei (n=28 samples) were loaded onto to the EvercodeTM WT Barcoding Plate configuration and split-pool barcoding was performed with the EvercodeTM WT v3 kit (Parse Biosciences) according to manufacturer instructions. Both initial DNA amplification and round 4 barcoding PCR were run with 7 cycles. The libraries were sequenced with an average depth of ~20k reads per cell.

### Sequence alignment and sample assignment

Raw cDNA libraries from 10x single-cell/nucleus samples (day 16, 25, 50, and 70) were processed with CellRanger[100] v7.1.0 using a human reference genome with optimized annotations[4]. The gene count matrices were corrected with CellBender[101] v0.3.0 to remove ambient RNA. The CellRanger BAM file for d16 data was processed with STARsolo[85] (STAR, v2.7.10b) to generate intronic and exonic counts. CITE-seq-Count v1.45[86] was used to identify the hashtag oligo (HTO) tags from the hashing FASTQ files. Followed by sample demultiplexing which was performed using an optimized cell classification strategy (based on the HTODemux Seurat function) implemented in the COMMUNEQAID pipeline (https://github.com/CBMR-Single-Cell-Omics-Platform/COMUNEQAID).

The raw reads from samples profiled using Parse were processed and aligned against the human GRCh38 genome using the Parse Biosciences Pipeline (v1.4.1).

### sc/snRNA-seq processing and clustering

From non-hashed Parse data doublets were identified and removed with scDblFinder (v1.16.0).[87] From 10x data, cells classified as doublets or negatives in the sample demultiplexing step were excluded. The resulting count matrices were filtered and processed with Scanpy[88] v1.9.5. Genes occurring in less than three cells, and cells with high mitochondrial content or outliers in gene counts,

were removed. Ribosomal (RPS/RPL) and mitochondrial (MT) genes were excluded to minimize metabolism-related bias. The resulting data was log-normalized and organized into three different 10x datasets d16, d25, and d50+70, and two Parse datasets d16_bmp and d50+80+d110_bmp, each processed and analyzed independently.

The d16 data underwent variable gene selection (n_top_genes=2000), dimensionality reduction (PCA and UMAP), and clustering (leidenalg v0.9.0). The d25, d50+70, d16_bmp and d50+80+d110_bmp datasets were processed with Seurat[89] v4.3.0, including variable gene selection, integration (RPCA), clustering, and annotation. To investigate the neuronal heterogeneity in d50+70 data, the cluster annotated as ARC neurons was subtracted and processed with Seurat as described above. Cluster annotations (except for d50+80+d110_bmp) were assigned based on prior knowledge and literature markers. The d50+80+d110_bmp data was annotated by first integrating it with annotated d50+70 data into a shared PCA space using Seurat and then predicting labels using K-nearest neighbor classification and pairwise cell distances (Scikit-learn, v1.6.0), followed by manual correction. Clusters deemed to be transcriptionally similar were merged. Consult the available code for the exact steps and parameters used in the steps described above. These processed datasets (d16, d25, d50+70, d50+70 neurons, d16_bmp, and d50+80+d110_bmp) formed the basis for downstream analysis (Tables S5 and S6).

### Trajectory inference

To assess the differentiation direction in d16 cells, we conducted RNA velocity analysis using the stochastic model from scVelo[90] v0.3.2. We used MetaNeighbor[50] (v1.22.0) to infer ARC neuronal lineages by linking clusters from d16 to d25 and d25 to d50+70. Only correlations with AUROC > 0.7 between adjacent timepoints were visualized in a Sankey plot, where branch color represents the AUROC values. To examine neuronal and tanycyte lineage separation, we integrated d16 cells with tanycytes from d25 and d50+70 using FastMNN[92] (batchelor v1.18.1). Followed by Monocle3[93] v1.3.7 to infer pseudotime lineages of developing tanycytes. Gene expression dynamics along pseudotime were modeled using polynomial fitting (NumPy v1.26.4, polyfit) and visualized using a heatmap.

### Differential gene expression analysis

We performed differential gene expression analysis using edgeR[94] v4.0.16. To analyze the response for FGF1 stimulation a pseudo-bulk gene expression matrix was generated by summing the transcript counts for all cells within the same cell type, differentiation, and treatment combination. Respectively, to identify genes with differential expression between cell types, we summed transcript counts within each cell type and differentiation batch. The pseudo-bulk gene expression matrix was then filtered and normalized. We used a generalized linear model implemented in glmQLFTest function (edgeR) to test for differential expression including treatment group and differentiation batch, or cell type and differentiation batch as variables in the design matrix. To highlight spatially relevant genes in the POMC subtype volcano plot, we used Seurat's FindAllMarkers to identify differentially expressed genes between ARC and VMH regions in the spatial human HYPOMAP.

### Transcriptomic comparison with human data

We used two publicly available sc/snRNA-seq datasets[20,39] from prenatal (gestational weeks 6 to 25) and adult human hypothalamus to explore transcriptional similarity and to compare gene expression profiles with our *in vitro* data. To assess similarity between our d50+70 dataset and the human hypothalamus, we reprocessed fetal and adult datasets, by removing remaining outlier cells using median absolute deviation (MAD) (SciPy v1.11.2) thresholding (consult the available code for MAD cutoffs), and excluding genes occurring in fewer than three cells, as well as ribosomal (RPS/RPL), and mitochondrial genes (MT). Fetal neuronal annotations were refined by aligning them with those from the adult human HYPOMAP, by leveraging the subset of adult cells shared across both studies, we integrated the adult and fetal neurons into a common PCA space with Seurat and applied a K-nearest neighbors classifier (Scikit-learn) to predict regional identities using adult data as a reference. Followed by integration and clustering of the fetal data, and manual correction of the predicted annotations.

To predict nuclei and cell type identities of our ARC cultures (d50+70 data), we used Seurat reference mapping approach, where the reprocessed fetal hypothalamic dataset was integrated, followed by transferring the cell type annotations to our data. Results were visualized with a bar plot showing the percentage of predicted cell types and a Sankey plot to illustrate the correspondence between our data and human fetal hypothalamus.

MetaNeighbor was used to assess similarity between our AGRP+/OTP+, GHRH+/PNOC+, and POMC+/TBX3+/NR5A2+ clusters (d50+70 neurons) and their corresponding subtypes in reprocessed fetal and adult human hypothalamus datasets. Missing subtype annotations in the fetal data were introduced through integration and clustering using Seurat. We also used MetaNeighbor to unravel how closely the two POMC subtypes (d50+80+d110_bmp) resemble ARC and VMH and various POMC clusters from fetal and adult hypothalamus.[39,69] Two additional human fetal hypothalamic clusters, POMC+/NR5A1+/TBX3- and POMC+/TBX3+/NR5A2-, were identified from developing human brain dataset using Gaussian mixture model (scikit-learn) based co-expression analysis of the respective markers and included in this analysis.

To resolve the spatial location of our d50+80+d110 bmp clusters we used Tangram (v1.0.4)[95] to project the clusters into a spatial section from human HYPOMAP spatial transcriptomic data. Clusters named telencephalic neurons, astrocytes and optic area neurons were excluded from the analysis. Tangram was executed in 'clusters' mode, using differentially expressed genes (identified via EdgeR, as described above) that were shared between the d50+80+d110 single-cell and spatial HYPOMAP datasets. To focus on the most confident predictions the top 20% of predictions scores were included to the visualization.

### Analysis of fetal mouse hypothalamic data

To explore POMC gene expression patterns across developing neuronal lineages in the mouse hypothalamus (E11-P8), we used publicly available scRNA-seq data.[71] For this analysis, the dataset underwent quality control to remove remaining low-quality cells, as well as ribosomal and mitochondrial genes. The neuronal lineages were subsetted and integrated using the Seurat (v5.3.1) workflow as described above. Gene expression patterns were visualized using UMAP.

### CELLECT analysis

To identify possible causal links between cell types generated here and metabolic traits, CELLECT[58] was used to integrate our d50+70 snRNA-seq with three relevant GWAS data sets and a GWAS for height as a negative control. For this purpose, we computed a cell type gene expression specificity matrix using CELLEX. Briefly, this algorithm calculates robust cell type specificity scores by averaging several different specificity metrics. The specificity matrix was then integrated with GWAS summary statistics using CELLECT (v 1.3.0)[58] under default parameters. This tool computes cell type prioritization scores by converting the specificity matrix into a genomic annotation, used as input for S-LDSC[102] which estimates GWAS enrichment in said annotations. We report, for each trait, a per cell type p-value of enrichment, corrected for multiple testing by the Benjamini–Hochberg procedure.

### Statistical analysis

All statistical tests were performed using a significance threshold of $\alpha = 0.05$. Graphs with error bars represent mean±SEM values, and statistical significance is indicated by asterisks corresponding to the test used. Data normality was assessed using the Shapiro-Wilk test. For normally distributed datasets, one-way ANOVA followed by Tukey's multiple comparisons test was applied. If variance was unequal, a Brown-Forsythe ANOVA with Dunnett's T3 multiple comparisons test was used. For non-normally distributed data, the Kruskal-Wallis test followed by Dunn's multiple comparisons test was performed. For pairwise comparisons, paired Student's t-tests were used for normally and log-normally distributed data, where the latter was log-transformed for the t-test. The Wilcoxon signed-rank test was used when distributions were non-normal. The statistical test used and p values are reported in the corresponding figure legends.

Cell Stem Cell, Volume *33*

## Supplemental Information

# Generation of human appetite-regulating neurons and tanycytes from pluripotent stem cells

**Zehra Abay-Nørgaard, Anika K. Mueller, Erno Hänninen, Dylan Rausch, Louise Piilgaard, Lucía Sena Trujillo, Lorenzo Fedrizzi, Jens Bager Christensen, Alison Salvador, Alrik L. Schörling, Noah Wulff Mottelson, Qiuyu Qin, Shruthi Sampath, Bob Hersbach, Jonas Henkenjohann, Sofie Peeters, Viktoriia Nikulina, Charlotte Høy Kruse, Yuan Li, Kavitha Chinnaiya, Marysia Placzek, Janko Kajtez, Tune H. Pers, and Agnete Kirkeby**

# Supplementary figures

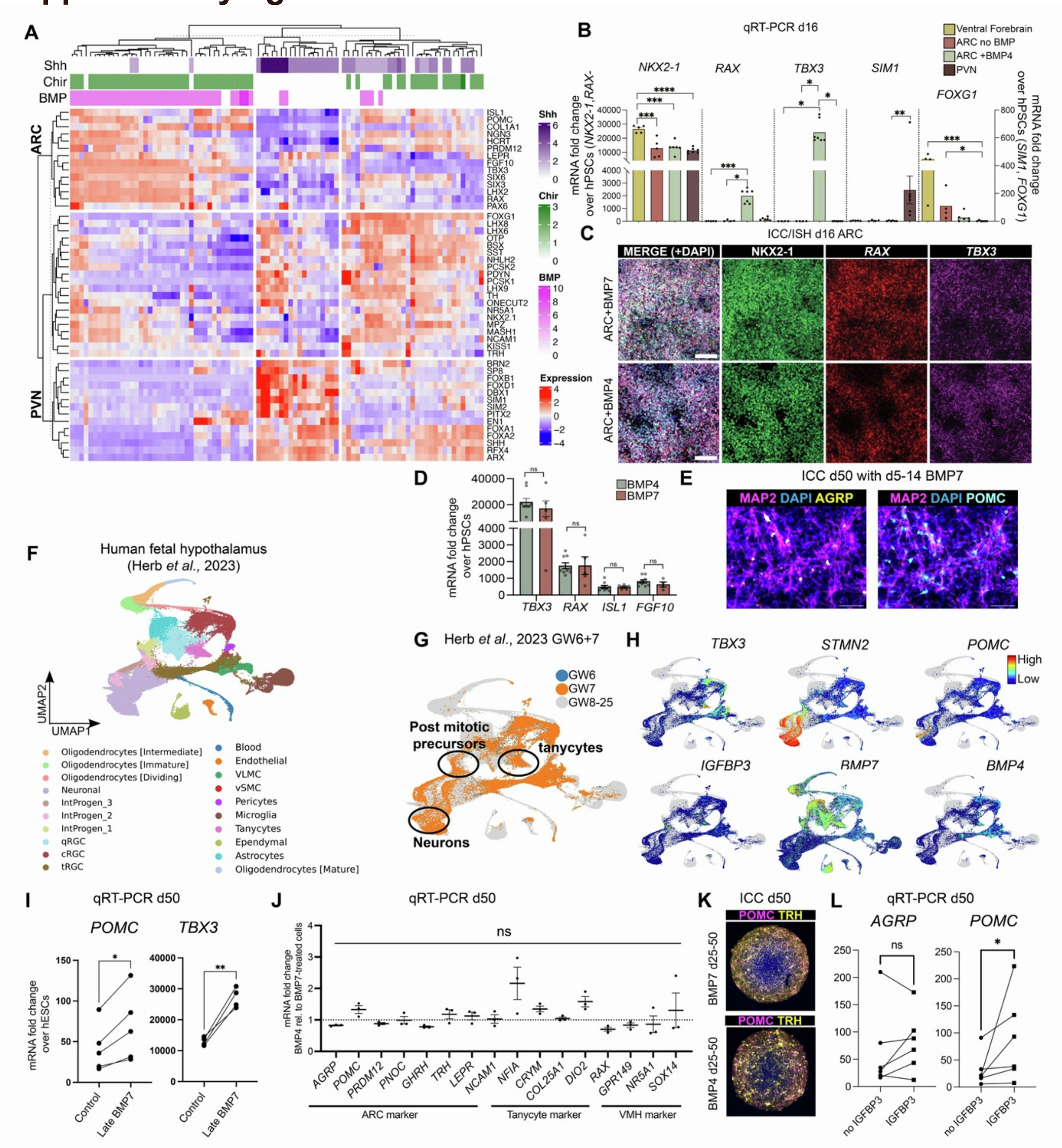


**Supplementary Figure 1. BMP4 modulates ARC and PVN differentiation profiles *in vitro*, related to Figure 1.**

(A) Unsupervised clustering of heatmap of d16 qRT-PCR of conditions with different morphogen timings of SHH, CHIR (Wnt agonist) and BMP (related to Figure 1B).

(B) qRT-PCR of d16 differentiations comparing PVN and ARC conditions with or without BMP4, and a ventral forebrain (VFB) differentiation as a reference, depicted as mean.

(C) ICC/ISH d16 of ARC cultures under BMP4 versus BMP7 treatment. Scale bars: 100 µm.

(D) qRT-PCR of d16 ARC treated with BMP4 or BMP7, mean ± SEM.

(E) ICC in d50 ARC cultures patterned with BMP7.

(F) UMAP of human fetal hypothalamic reference with annotated clusters[1].

(G) UMAP of scRNA-seq from gestational weeks 6–25 showing the contribution from early (GW6–7) samples[1].

(H) Feature plots of key hypothalamic markers from fetal human reference data[1].

(I) qRT-PCR of d50 ARC cultures ± BMP7.

(J) qRT-PCR and (K) ICC of d50 ARC with d25-50 BMP4 or BMP7 (n=3 RC17 hESC). Dotted line is average of BMP7-treated samples, mean ± SEM.

(L) qRT-PCR of day 50 ARC cultures ± IGFBP3.
Statistics: (B) *NKX2-1* (One-way ANOVA with Tukey's test): VFB vs. No BMP p = 0.0002; VFB vs. BMP4 p = 0.0003; VFB vs. PVN p < 0.0001, *RAX* (Kruskal-Wallis with Dunn's test): VFB vs. BMP p = 0.0006; No BMP vs. BMP p = 0.0156, *TBX3*: VFB vs. BMP p = 0.0280; No BMP vs. BMP p = 0.0341; BMP vs. PVN p = 0.0225, *SIM1*: BMP4 vs. PVN p = 0.0079. *FOXG1*: VFB vs. PVN p = 0.0010; No BMP vs. PVN p = 0.0466
(D) BMP4 vs. BMP7: Unpaired t-test; ns, not significant (I) *POMC*: Paired t-test, p = 0.0154; n = 5, TBX3: Paired t-test, p = 0.0043; n = 4 (J) Wilcoxon matched-pairs signed rank test with two-stage step up of Benjamini, Krieger and Yekutieli; all ns. (L) *AGRP*: Wilcoxon matched-pairs signed rank test, n = 6, p = 0.0563 POMC: p = 0.0312

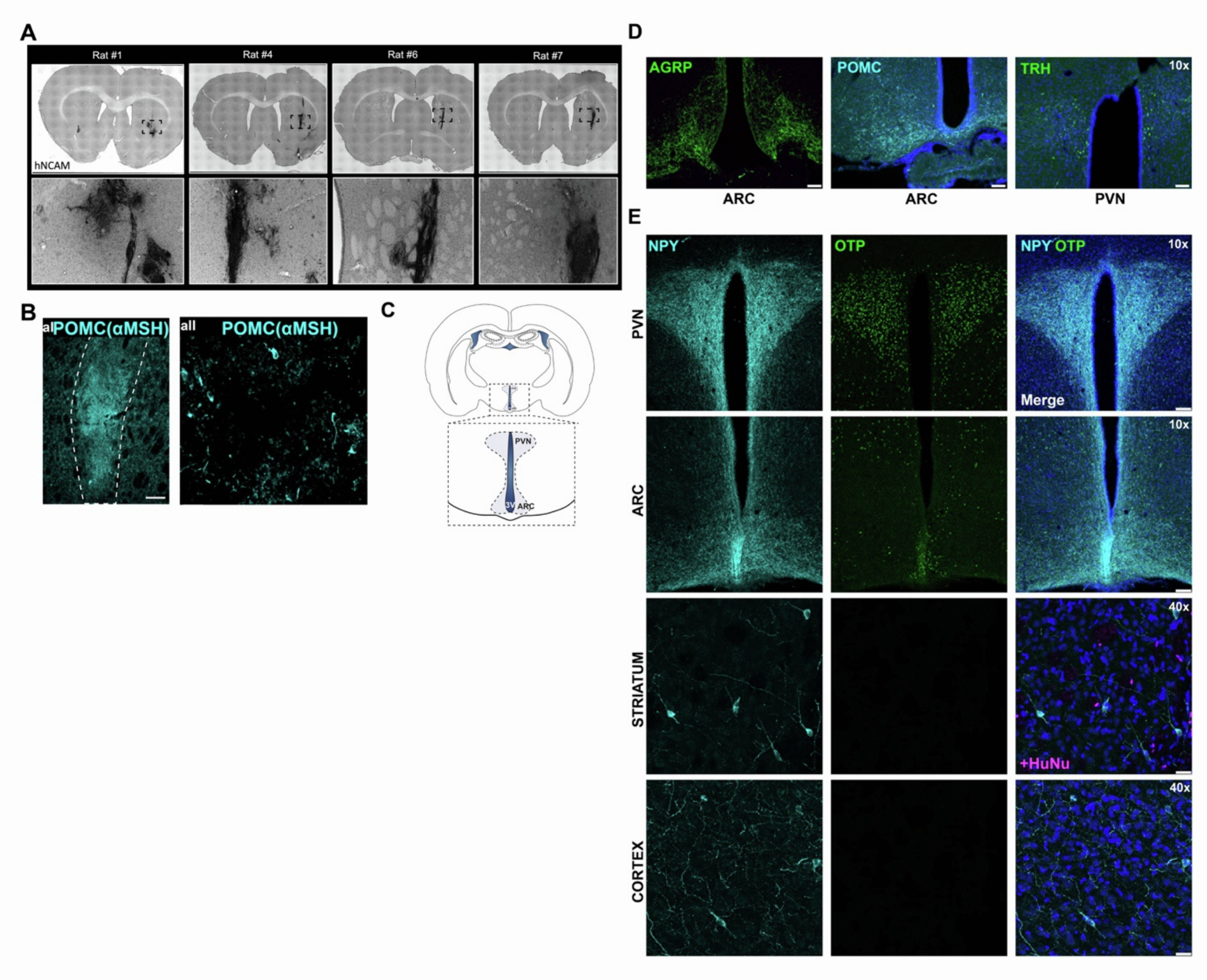


**Supplementary Figure 2. Validation of ARC graft identity and NPY expression *in vivo*, related to Figure 1.**

(A) Xenografts fixed for IHC from n=4 animals showing hNCAM labelling human neurons in the rat striatum.

(B) ICC showing POMC (αMSH) cells within the xenograft (aI) and at higher magnification (aII). Dotted line denotes graft boundary. Scale bars: 200 μm (aI), 25 μm (aII).

(C) Schematic of a coronal rat brain section depicting the anatomical positions of the ARC and PVN.

(D) Antibody staining for AGRP, POMC, and TRH in native rat hypothalamic sections to validate antibody specificity. Scale bars: 100 μm.

(E) Antibody staining for NPY and OTP in rat brain sections. ARC neurons show distinct NPY localization and morphology compared to NPY

interneurons in the striatum and cortex. Scale bars: 200 μm (ARC, PVN), 50 μm (striatum, cortex)

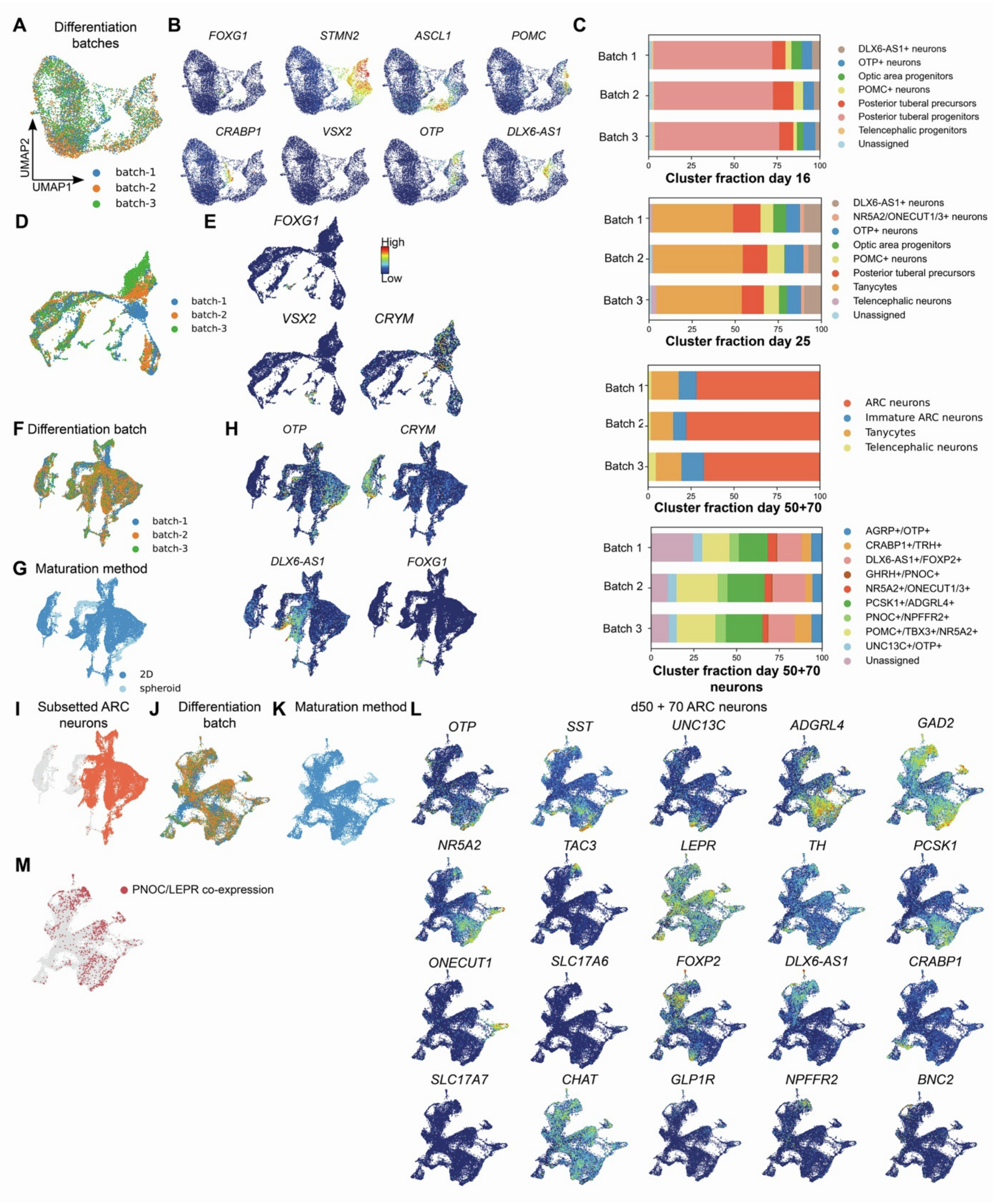


**Supplementary Figure 3. Single-cell and single-nucleus profiling of ARC cultures across developmental timepoints, related to Figure 2.**
(A) UMAP of scRNA-seq data from d16 ARC cultures (n = 3; RC17) colored by replicates (batches).
(B) Feature plots of key marker genes in the d16 dataset.
(C) Bar plots showing the distribution of cell types across d16 (top), and later timepoints (d25, d50+70, day 50+70 neurons; bottom) (n = 3; RC17).
(D) UMAP of d25 snRNA-seq data colored by differentiation batch (n = 3; RC17).
(E) Feature plots of markers for tanycytes, telencephalic, and eye field progenitors.
(F) UMAP of d50+70 snRNA-seq data colored by differentiation batch (n = 3; RC17).
(G) UMAP of d50+70 snRNA-seq data colored by 2D versus 3D maturation conditions.
(H) Feature plots of key markers from d50+70 ARC snRNA-seq data.
(I) UMAP showing ARC neuron cluster subsetted from d50+70 dataset.
(J) UMAP of ARC neurons at d50+70 colored by differentiation batch (n = 3; RC17).

(K) UMAP of ARC neurons at day 50+70 colored by maturation condition (2D vs. 3D).
(L) Feature plots of selected marker genes in day 50+70 ARC neurons.
(M) UMAP of neuronal cells co-expressing *PNOC* and *LEPR* in the day 50+70 dataset.

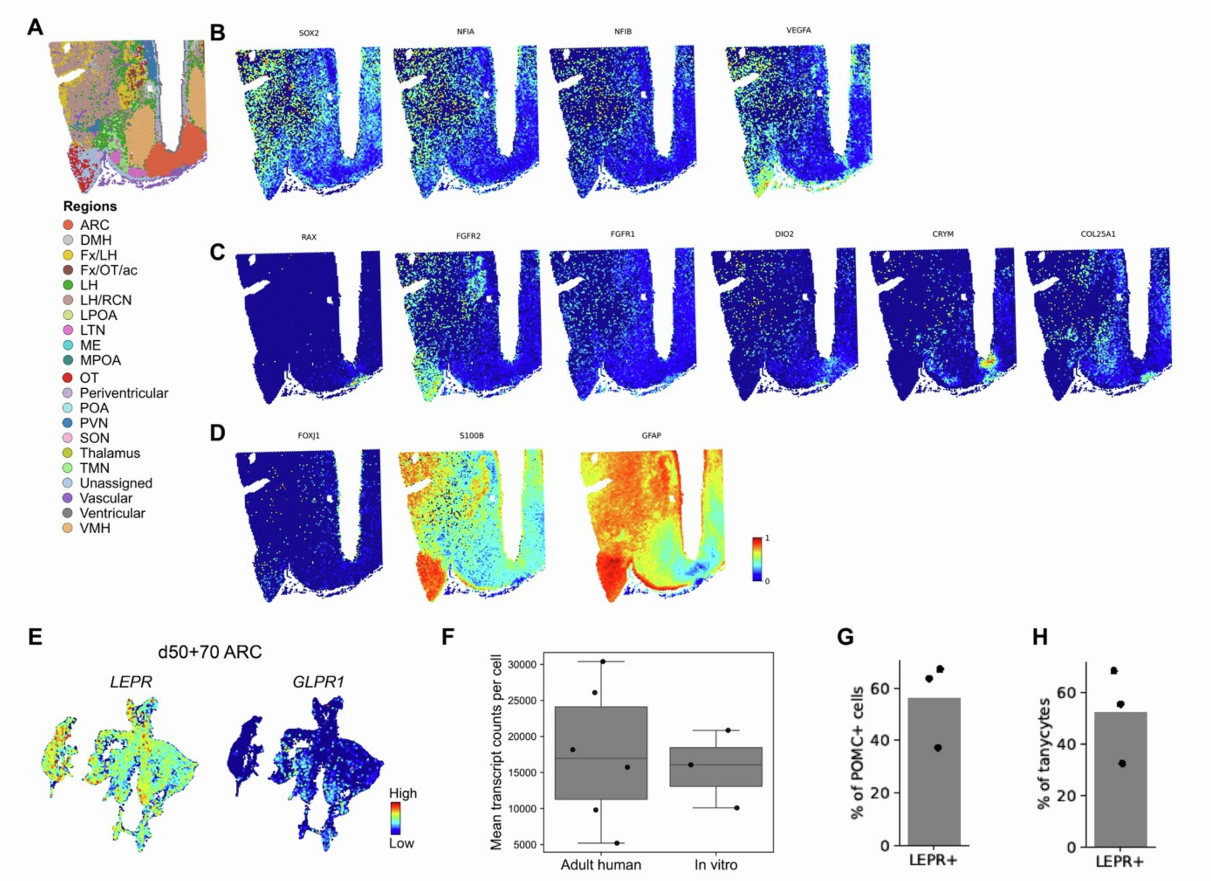


**Supplementary Figure 4. Spatial transcriptomics of tanycyte marker and cluster-specific *LEPR* expression, related to Figure 4.**

(A) Spatial transcriptomics tissue-section plot from adult human HYPOMAP[2] showing regional annotations.

(B-D) Spatial HYPOMAP[2] showing expression patterns for general (B), β (C), and α (D) tanycyte markers.

(E) UMAPs of *LEPR* and *GLP1R* at d50+70, annotations in Figure 2E.

(F) Tukey's box plot comparing mean transcript counts per cell between adult human HYPOMAP[2] and d50+70 ARC scRNA-seq data (from Figure 2E). Each dot represents a donor in HYPOMAP data and a differentiation batch in *in vitro data.*

(G) Bar plot quantifying co-expression of POMC and LEPR in POMC+ neurons (data from Figure 2G). Each dot represents a differentiation batch.

(H) Bar plot quantifying LEPR expression in tanycytes (data from Figure 2E). Each dot represents a differentiation batch.

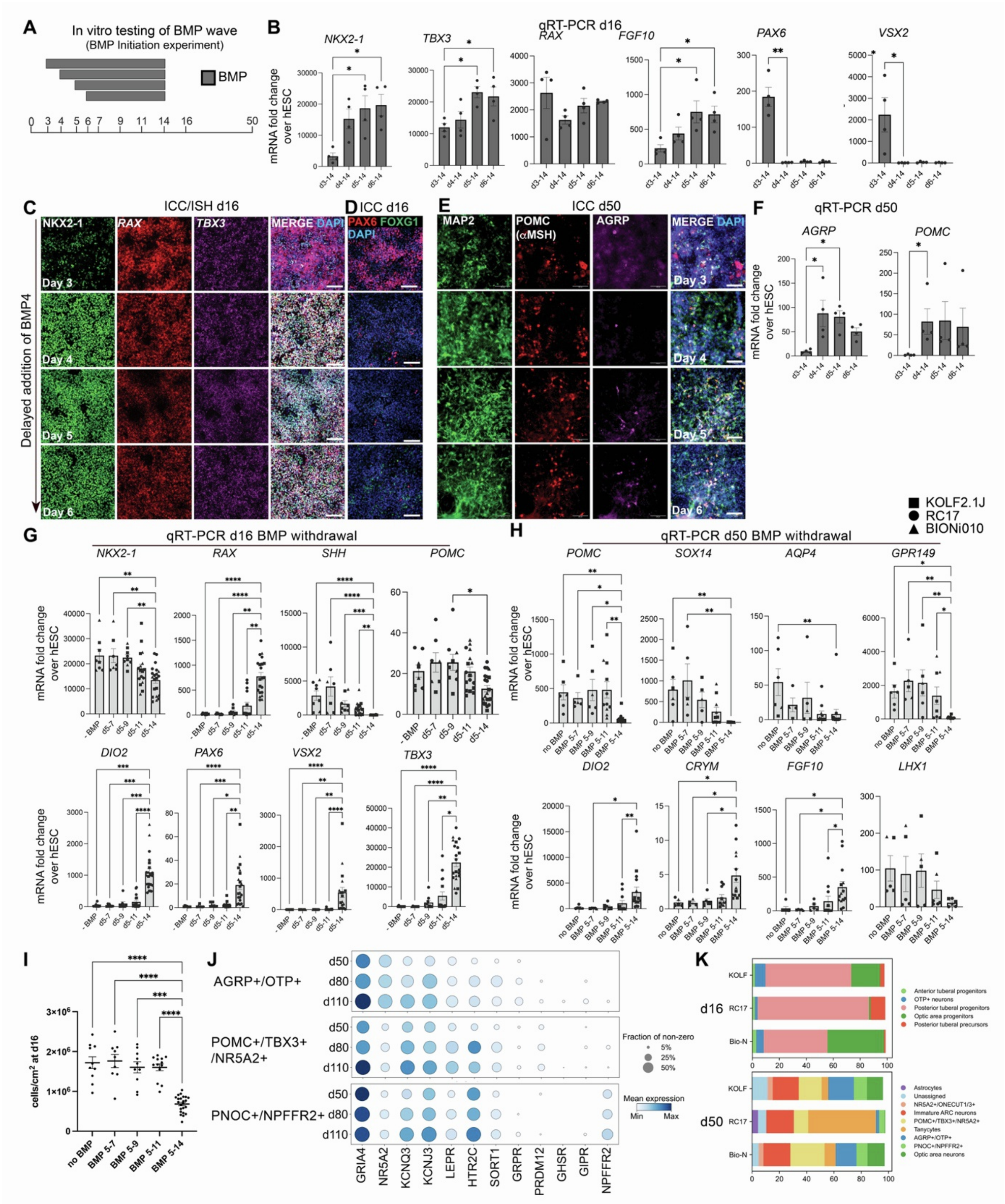


**Supplementary Figure 5. Early BMP4 exposure timing modulates ARC patterning and generated neurons, related to Figure 5.**

(A) Experimental timeline of BMP4 addition from either day 3, 4, 5, or 6 until day 14.

(B) qRT-PCR analysis of day 16 cultures (n = 4) patterned with different BMP4 start times.

(C) ICC for NKX2-1 and ISH for *RAX* and *TBX3* on day 16 ARC cultures exposed to BMP4 at different initiation points. Scale bar: 100 μm.

(D) ICC of FOXG1 and PAX6 expression at day 16 under various BMP4 exposure timings. Scale bar: 100 μm.

(E) ICC of POMC (αMSH), AGRP, and MAP2 expression in day 50 ARC cultures treated with different BMP4 start times. Scale bar: 100 μm.

(F) qRT-PCR of day 50 ARC cultures (n = 4) treated with BMP4 from day 3–6 until day 14.

(G) qRT-PCR of three cell lines at day 16 with BMP4 added from day 5 to day 7, 9, 11, or 14.

(H) qRT-PCR of three cell lines at day 50 under same BMP4 conditions.

(I) Cell counts of differentiations (RC17, KOLF2.1J, BIONi010) after dissociation at d16 treated with BMP4 for different timepoints.
(J) Dot plots showing trend towards increased expression of selected genes in ARC clusters over a time course spanning three timepoints from day 50 to 110 (n=1, RC17 hESC, BMP4 5-14).
(K) Bar plots showing distribution of cell types across cell lines in BMP4 5-14 snRNA-seq data (from Figure 5F-G) at d16 and d50.
Statistics: All bar plots and dot plots represent mean ± SEM. (B) One-way ANOVA with Tukey's test: NKX2-1: d3–14 vs. d5–14 p = 0.0211; d3–14 vs. d6–14 p = 0.0141. *TBX3*: d3–14 vs. d5–14 p = 0.0208; d3–14 vs. d6–14 p = 0.0432. *FGF10*: d3–14 vs. d5–14 p = 0.0293; d3–14 vs. d6–14 p = 0.0437. Kruskal-Wallis: *RAX*: ns; PAX6: d3–14 vs. d4–14 p = 0.0050; *VSX2*: d3–14 vs. d4–14 p = 0.0140 (F) *AGRP* (ANOVA): d3–14 vs. d4–14 p = 0.0190; d3–14 vs. d5–14 p = 0.0332.*POMC* (Kruskal-Wallis): d3–14 vs. d4–14 p = 0.0227. (G) *NKX2-1* (ANOVA): No BMP vs. d5–14 p = 0.0038; d5–7 vs. d5–14 p = 0.0069; d5–9 vs. d5–14 p = 0.0067 Kruskal-Wallis: *RAX*: No BMP vs. d5–14 p < 0.0001; d5–7 vs. d5–14 p = 0.0004; d5–9 vs. d5–14 p = 0.0055; d5–11 vs. d5–14 p = 0.0074. *SHH*: No BMP vs. d5–14 p < 0.0001; d5–7 vs. d5–14 p < 0.0001; d5–9 vs. d5–14 p = 0.0006; d5–11 vs. d5–14 p = 0.0016. *POMC*: d5–9 vs. d5–14 p = 0.0273. *DIO2*: No BMP vs. d5–14 p = 0.0002; d5–7 vs. d5–14 p = 0.0001; d5–9 vs. d5–14 p = 0.0002; d5–11 vs. d5–14 p < 0.0001. *PAX6*: No BMP vs. d5–14 p < 0.0001; d5–7 vs. d5–14 p = 0.0004; d5–9 vs. d5–14 p = 0.0352; d5–11 vs. d5–14 p = 0.0018. *VSX2*: No BMP vs. d5–14 p < 0.0001; d5–7 vs. d5–14 p = 0.0013; d5–9 vs. d5–14 p = 0.0011; d5–11 vs. d5–14 p < 0.0001. (H) Kruskal-Wallis: *POMC*: No BMP vs. d5–14 p = 0.0068; d5–7 vs. d5–14 p = 0.0205; d5–9 vs. d5–14 p = 0.0124; d5–11 vs. d5–14 p = 0.0012. *SOX14*: No BMP vs. d5–14 p = 0.0025; d5–7 vs. d5–14 p = 0.0045. *AQP4*: No BMP vs. d5–14 p = 0.0060. *GPR149*: No BMP vs. d5–14 p = 0.0108; d5–7 vs. d5–14 p = 0.0045; d5–9 vs. d5–14 p = 0.0078; d5–11 vs. d5–14 p = 0.0454. *DIO2*: d5–7 vs. d5–14 p = 0.0268; d5–11 vs. d5–14 p = 0.0070. *CRYM*: No BMP vs. d5–14 p = 0.0140; d5–7 vs. d5–14 p = 0.0354; d5–9 vs. d5–14 p = 0.0364. *FGF10*: No BMP vs. d5–14 p = 0.0139; d5–7 vs. d5–14 p = 0.0362; d5–11 vs. d5–14 p = 0.0229. (I) Kruskal-Wallis: no BMP bs. BMP5-14 p<0.0001; BMP5-7 vs. BMP5-14 p<0.0001; BMP5-9 vs. BMP5-14 p = 0.0003; BMP5-11 vs. BMP5-14 p<0.0001.

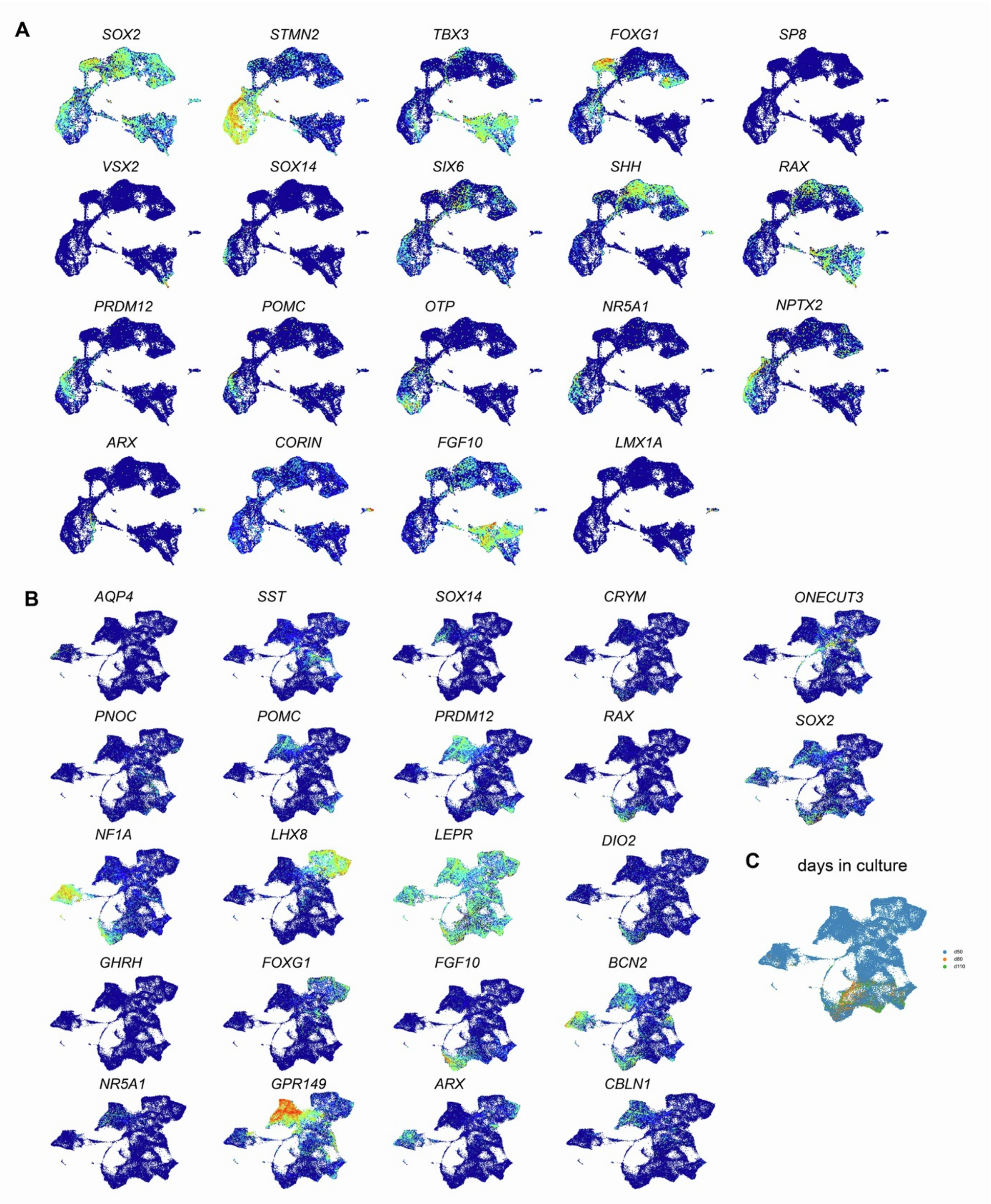


**Supplementary Figure 6. Single-cell transcriptomic analysis of BMP4 withdrawal on ARC cultures, related to Figure 5.**

(A) Feature plots of key hypothalamic patterning markers in the d16 dataset under different BMP4 conditions.

(B) Feature plots of relevant hypothalamic and lineage-specific markers in d50+80+110 ARC cultures patterned under different BMP4 regimens.

(C) UMAP of d50+80+110 cultures colored by timepoint.

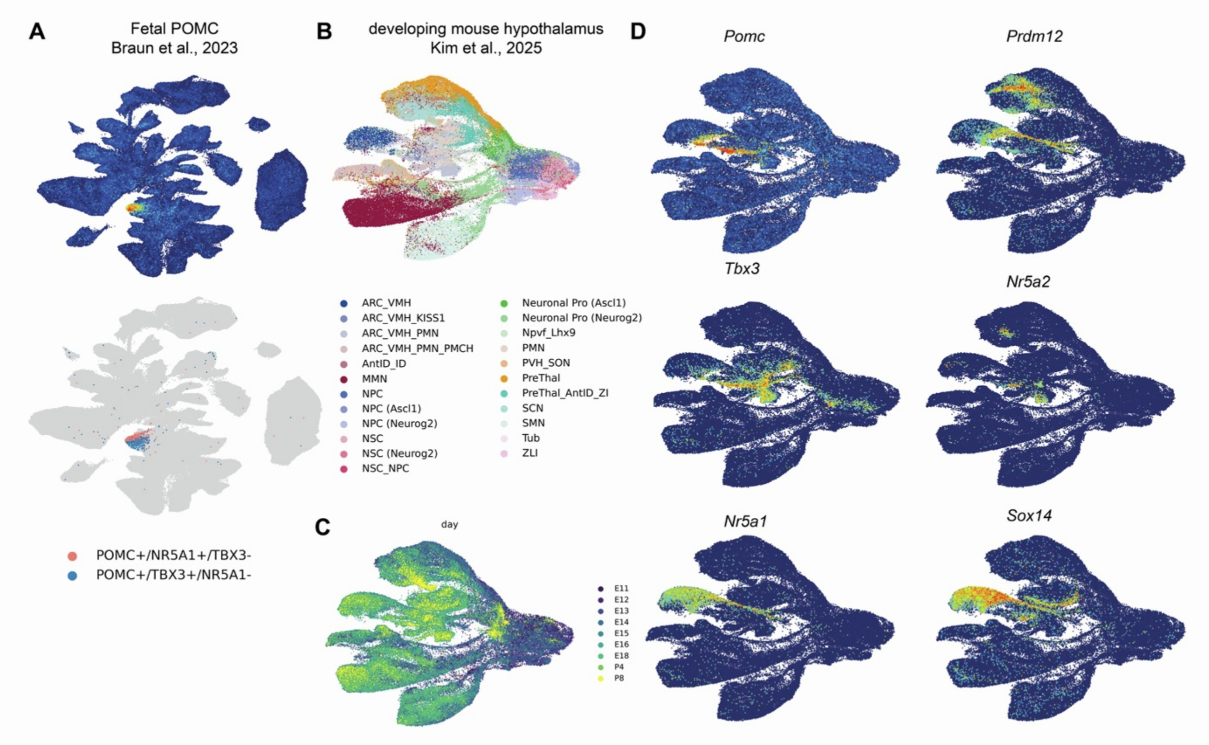


**Supplementary Figure 7. Developmental origin of distinct POMC clusters, related to Figure 6.**
(A) Top: UMAP of the developing human brain atlas (trimester 1) from Braun *et al.*, 2023[3] showing spatial expression of POMC across early brain regions. Bottom: UMAP showing two distinct POMC-expressing clusters identified by co-expression analysis in the fetal brain dataset, corresponding to ARC- and VMH-like populations.
(B) UMAP showing cluster annotations in the developing mouse hypothalamus[3].
(C) UMAP showing developmental time points in the developing mouse hypothalamus.
(D) UMAP showing gene expression patterns of POMC and regional ARC and VMH markers in the developing mouse hypothalamus.

## Supplementary tables

| Gene symbol | Gene name | Forward primer sequence | Reverse primer sequence |
|---|---|---|---|
| *ACTB* | Actin beta | ATGTGGCCGAGGACTTTGATTG | ATGGCAAGGGACTTCCTGTAAC |
| *AGRP* | Agouti related neuropeptide | TCCCTGTCCTGTGGAAATTTGT | TCTCCAATTTGGGGTGAGGTTT |
| *AQP4* | Aquaporin 4 | TTGGACCTGCAGTTATCATGGG | CTATGATGGGCCCAACCCAATA |
| *ARX* | Aristaless related homeobox | CCTGAGCACTTTCCTCGGAGCG | TGGAAAAGAGCCTGCCGAATGCC |
| *BRN2* | POU3F2, POU class 3 homeobox 2 | ATGCGCGGCTCCTTTAACCGG | TTAGACGCTGCGGTCGCCATG |
| *BSX* | Brain specific homeobox | GAGAAGAGGTTCGAGATCCAGC | GTTCTGGAACCACGTTTTCACC |
| *CART* | CART prepropeptide | GCGTCCATTCTCCTCCATACAT | GTTGCTTAAGCCAAACTCCAGG |
| *CRH* | Corticotropin releasing hormone | CTGTACCATAGCGCTGCTCTTA | TTGTGCATGCTAAGTAAGGGGT |
| *CITED1* | Cbp/p300 interacting transactivator with Glu/Asp rich carboxy-terminal domain 1 | AGTGGATGAGGAAGTGCTGATG | AAGTCAAACTCATTCTGCCCCA |
| *COL1A1* | Collagen type I alpha 1 chain | CTTATGAAACCCCAATGCTGCC | GGGAGACAGATTTGGGAAGGAG |
| *COL25A1* | Collagen type XXV alpha 1 chain | GACCCAGGTATGACAGGTGAAAA | CATTCCATTGGCTCCCGGTAA |
| *CRYM* | Crystallin mu | ACAGAGCCCATTTTGTTTGGTG | CATCCAGTTCTCTCCAGTCAGG |
| *DBX1* | Developing brain homeobox 1 | GAAGTTTGGAGTGAACGCCATC | GACCCTTCGAAGTAGGGAAAGG |
| *DIO2* | Iodothyronine Deiodinase 2 | GTTATAAGGCAACCCCCGGTAT | AACCTCAGCTAATGGGACCAAG |
| *DLX2* | Distal-less homeobox 2 | ACCAGACCTCGGGATCCGCC | CTGCGGGGTCTGAGTGGGGT |
| *EN1* | Engrailed homeobox 1 | CGTGGCTTACTCCCCATTTA | TCTCGCTGTCTCTCCCTCTC |
| *FEZF1* | FEZ family zinc finger 1 | GGTACATTCCACATTCGTGAGC | TCACGTGCAATAATCAAAACCA |
| *FGF10* | Fibroblast growth factor 10 | GTTGCTGTTCTTGGTGTCTTCC | TGACACCATGTCCTGACCAAG |
| *FOXA1* | Forkhead box A1 | GGGCAGGGTGGCTCCAGGAT | TGCTGACCGGGACGGAGGAG |
| *FOXA2* | Forkhead box A2 | CCGTTCTCCATCAACAACCT | GGGGTAGTGCATCACCTGTT |
| *FOXB1* | Forkhead box B1 | GTGGTCGGACTTAAGCACCTT | GTGGTCGGACTTAAGCACCTT |
| *FOXD1* | Forkhead box D1 | CCTGTCCAGTGTCGAGAACTTT | AACCACCAAGACGAGAAAAGGA |
| *FOXG1* | Forkhead box G1 | TCAACGGCATCTACGAGTTCAT | AAGCACTTGTTGAGGGACAGAT |

| | | | |
|---|---|---|---|
| *GAPDH* | Glyceraldehyde-3-phosphate dehydrogenase | TTGAGGTCAATGAAGGGGTC | GAAGGTGAAGGTCGGAGTCA |
| *GBX2* | Gastrulation brain homeobox 2 | GTTCCCGCCGTCGCTGATGAT | GCCGGTGTAGACGAAATGGCCG |
| *GHRH* | Growth hormone releasing hormone | AGGAACTCCCAGGGATGAAGAT | CAGTTGCATTTTGGCTACAGGT |
| *GRH* | Growth hormone receptor | ACTAGCAATGGTGGTACAGTGG | TCAGTAAAGTCCAGTTGAGGGC |
| *GPR149* | G protein-coupled receptor 149 | GACTGGGAGTGGTGTAGGAGTA | GGCATAACCGGAACGCTGAC |
| *HCRT* | Hypocretin neuropeptide predursor | CCTCAAGGTTCCTGGCTTTTTG | GGAAGGAAGGTTCATGGTGTCT |
| *ISL1* | ISL LIM homeobox 1 | AAGCGCAGGAAGAGAGACTG | CCAAGAGACCCAGGATTTCA |
| *KISS1* | KiSS-1 metastasis suppressor | TGAACTTCAGACCCCAAAGGAG | TCTTTTATTGCCTCGGGTTGGA |
| *LEPR* | Leprin receptor | TACTGTTACGGTTCTGGCCATC | TGCTCATAGGCCATGAAAAGGT |
| *LHX2* | LIM homeobox 2 | GGGCGACCACTTCGGCATGAA | CGTCGGCATGGTTGAAGTGTGC |
| *LHX6* | LIM Homeobox 6 | AGGCAAGAACATCTGCTCCAG | GCCAGATGAGGTTGTTGACCTT |
| *LHX8* | LIM Homeobox 6 | AGGCAAGAACATCTGCTCCAG | GCCAGATGAGGTTGTTGACCTT |
| *LHX9* | LIM Homeobox 9 | TGCCTGAAGTGCTGTGAATGTA | TTGCAGTAAATGCTACCGTCCT |
| *MASH1* | ASCL1, achaete-scute family bHLH transcription factor 1 | CTAAAGATGCAGGTTGTGCG | GGAGCTTCTCGACTTCACCA |
| *MC4R* | Melancortin 4 receptor | CTTTTTCATCTGCAGCTTGGCT | TGTGAAACTCTGTGCATCCGTA |
| *MCH* | Melanin concentrating hormone | TACATTCAGGTTGGGGAAAGGC | CCAGGGAAGGAGCAATAACTGA |
| *MPZ* | Myelin protein zero | CTCAGGTCACGCTGTATGTCTT | GAACCACGTAGAAAAGCAGCAG |
| *NCAM1* | Neural cell adhesion molecule 1 | GTCAGAGGCCACCGTCAACGTG | CTTCCCCCTCCCGGAACTCCTG |
| *NGN3* | Neurogenin 3 | CTGAACTTGGCGACCAGAAGC | TTGAGGCGTCATCCTTTCTACC |
| *NHLH2* | Nescient helix-loop-helix 2 | ACCCACTGGAGACTTTGAGTTC | GCATACTCTGAACTTCTGCCCT |
| *NKX2.1* | NK2 homeobox 1 | AGGGCGGGGCACAGATTGGA | GCTGGCAGAGTGTGCCCAGA |
| *NPY* | Neuropeptide Y | GAAAATGTTCCCAGAACTCGGC | TAGGAAAAGGCCAGAGAGCAAG |
| *NR5A1* | Nuclear receptor subfamily 5 group A member 1 | GGAACAAGTTTGGGCCGATGTA | GTGCCTTCTTCTGCTGTTTCAG |
| *NROB1* | Nuclear receptor subfamily 0 group B member 1 | GCCATCAAGTGCTTTCTTTCCA | TAGGCGTACTCCTTGGTACTGA |
| *NTS* | Homo sapiens neurotensin | GCTCAAAGTACTACAGCAAAGCC | GGCGCTATTACTTTGTTTTGGGT |
| *ONECUT2* | One cut homeobox 2 | AAGGGGTAGAGCTGGTGTATCT | TGTTGTTTCAGGGGTGACTTGA |

| | | | |
|---|---|---|---|
| *OTP* | Orthopedia homeobox | TAGAAGGGAAGGC TTCTCAGGA | GTCAGATCACCTCTTC CTCGTC |
| *OTX2* | Orthodenticle homeobox 2 | ACAAGTGGCCAAT TCACTCC | GAGGTGGACAAGGGA TCTGA |
| *PAX6* | Paired box 6 | TGGTATTCTCTCC CCCTCCT | TAAGGATGTTGAACGG GCAG |
| *PCSK1* | Proprotein convertase subtilisin/kexin type 1 | GCAATGCCCGTAA TGCTTAGAG | TTCCAAGGACAGAGT GATTCCG |
| *PCSK2* | Proprotein convertase subtilisin/kexin type 2 | GATTGACTATCTCC ACCCGGAC | TCTGTGTACCGAGGG TAAGGAT |
| *PDYN* | Prodynorphin | CTCATTCCCAGGC ACTCTCTTT | TTTCCTCTCCTATCCA GCCTCA |
| *PITX2* | Paired like homeodomain 2 | AACTCTATGAACG TCAACCCCC | CGACATGCTCATGGA CGAGATA |
| *POMC* | Proopiomelanocortin | TTTCATGACCTCC GAGAAGAGC | GATGATGGCGTTTTTG AACAGC |
| *PNOC* | Prepronociceptin | AGTGTGTTCAGCA GTTGTCAGA | ACCTTCTCTTCACACT CGAGGA |
| *PRDM12* | PR/SET domain 12 | GTGGGAGGTGTTC AATGAGGAT | GTTCGTTACGTGCACA CTTGAT |
| *PVALB* | Parvalbumin | GGACAAGGACAAA AGTGGCTTC | CAGCCATCAGCATCTT GGTTTC |
| *RAX* | Retina and anterior neural fold homebox | CCTCTCAGTTCAC CAAGCAGAT | TGATCAACCTTGGGT GTTAGGG |
| *RFX4* | Regulatory factor X4 | ACCTTGCCATCTG TCTTGTCAT | ATAGGGATGGTACCAC CAGGAA |
| *SHH* | Sonic hedgehog | CCAATTACAACCC CGACATC | AGTTTCACTCCTGGC CACTG |
| *SIM1* | Single-minded family bHLH transcription factor 1 | AAAGGGGGCCAA ATCCCGGC | TCCGCCCCACTGGCT GTCAT |
| *SIM2* | SIM bHLH transcription factor 2 | GGCTACTTGAAGA TCAGGCAGT | ATCTGGTAGCAGGAG TCGTACA |
| *SIX3* | SIX homeobox 3 | ACCGGCCTCACTC CCACACA | CGCTCGGTCCAATGG CCTGG |
| *SIX6* | SIX homeobox 6 | CTCAACAAGAATG AGTCGGTGC | ACTCCTTGGTGAACTT GTGGTT |
| *SOX14* | SRY-box 14 | CATACATCGATGAA GCCAAGCG | CTGTCCTTCTTGAGCA GGTTCT |
| *SP8* | Sp8 transcription factor | CCTGTCTGTCCGG ACTTCAA | AGGGGCAGAAACAGA AAGAGAC |
| *SP9* | Sp9 transcription factor | TCTGGCCCCAACG ACTCTTAG | CTCGTTCGTTCTCGGT GTCTC |
| *SST* | Somatostatin | GGAACCTGAAGAT CTGTCCCAG | ATAGCCGGGTTTGAGT TAGCAG |
| *TBX3* | T-box transcription factor 3 | GGGGGTAGGAGTT CCAACATTT | GCACTGAGGGAGATG TCTTTGA |
| *TH* | Tyrosine hydroxylase | CGGGCTTCTCGGA CCAGGTGTA | CTCCTCGGCGGTGTA CTCCACA |
| *TRH* | Thyrotropin releasing hormone | TCCTGGATGACCT GAGTAGGAG | TCAGGGAAAACTGGG TTCACTC |
| *VSX2* | Unc-5 netrin receptor D | CCTCTGCCCTCTG TAAATGTGT | AGAGACCTCTGCGAG AACTTTG |
| | | | |

**Supplementary Table 1:** List of qRT-PCR primers, related to STAR methods.

| Antigen | Host species | Dilution | Manufacturer (#Cat) | Reacts with human? | Validated in rat in-house? |
|---|---|---|---|---|---|
| NKX2-1 | Rabbit | 1:100-200 | Abcam (#ab1333737) | yes | |
| PAX6 | Mouse | 1:1000 | Sigma-Merck (#AMAb91372) | yes | |
| AGRP | Rabbit | 1:1000 | Phoenix Pharma (#H-003-53) – discontinued | yes | yes |
| AGRP | Goat | 1:100-200 | R&D Systems (#AF634) | | yes |
| POMC (aMSH) | Sheep | 1:1000 | Millipore (#AB5087) | Unknown, "mammals" | yes |
| MAP2 | Mouse | 1:1000 | Sigma (#M1406) | yes | |
| TRH | Rabbit | 1:1000 | Thermo Fisher (PA5-57331) | yes | yes |
| TH | Mouse | 1:1000 | Immunostar (173-22941) | yes | yes |
| GHRH | Rabbit | 1:200 | Abcam (ab18751) | yes | |
| OTP | Rabbit | 1:500 | GeneTex (GTX119601) | yes | yes |
| FOXG1 | Rabbit | 1:500 | Abcam (#ab18259) | yes | |
| CRH | Rabbit | 1:1000 | Proteintech (10944-1-AP) | yes | |
| AQP4 | Rabbit | 1:1000 | Sigma-Merck (#HPA014784) | yes | yes |
| hNCAM1 | Mouse | 1:1000 | Santa Cruz Biotechnology #SC-106 | yes | |
| HuNu | Mouse | IF: 1:1000 ISH: 1:200 | Millipore #AB1281 | yes | yes |
| NPY | Sheep | 1:500 | Millipore #AB1583 | | yes |
| S100b | Mouse | 1:1000 | Sigma (#S2532) | yes | yes |
| Somatostatin | Mouse coupled-Alexa Fluor 488 | 1:500 | BD Biosciences (#566032) | yes | yes |
| NFIA | Rabbit | 1:200 | Abcam (#ab228897) | yes | yes |
| Vimentin | Chicken | 1:500 | Millipore (#AB5733) | yes | |
| NR5A1 | Mouse | 1:1000 | Thermo Fisher (#434200) | yes | yes |

| | | | | |
|---|---|---|---|---|
| HuC/D | Mouse | 1:400 | Invitrogen (A-21271) | yes |
| bMSH | Rabbit | | LSBio #LS-C183969-50) | yes |

**Supplementary Table 2:** List of primary antibodies, related to STAR methods.

| ANTIGEN | FLUOROPHORE | DILUTION | MANUFACTURER (#CAT) |
|---|---|---|---|
| RABBIT | Alexa488 | 1:200-500 | Jackson Imm. Res. (#711-545-152) |
| MOUSE | Alexa647 | 1:200-500 | Jackson Imm. Res. (#711-605-151) |
| SHEEP | Alexa Cy3 | 1:200-500 | Jackson Imm. Res. (#713-165-147) |
| MOUSE | Alexa Cy3 | 1:200 | Jackson Imm. Res. (#715-165-151) |
| MOUSE | Alexa 488 | 1:200-500 | Jackson Imm. Res. (#715-545-150) |
| RABBIT | Alexa CY3 | 1:200 | Jackson Imm. Res. (#711-165-152) |
| GOAT | Alexa488 | 1:200-500 | Jackson Imm. Res. (#705-545-147) |
| SHEEP | Alexa647 | 1:200-500 | Jackson Imm. Res. (#713-605-147) |
| GOAT | Alexa647 | 1:200 | Jackson Imm. Res. (#705-605-147) |
| MOUSE | Biotinylated | 1:500 | Vector Laboratories #BA9200 |
| DAPI | - | 1:500 | Thermo (#D3571) |

**Supplementary table 3.** List of secondary antibodies, related to STAR methods.

| Probe | Host species | Dilution | Manufacturer (#Cat) |
|---|---|---|---|
| Hs-RAX-C1 | Homo sapiens | Ready-to-use | ACD Bio-Techne (#579951) |
| Hs-TBX3-C2 | Homo sapiens | 1:50 | ACD Bio-Techne (#557441) |
| Hs-PNOC-C2 | Homo sapiens | 1:50 | ACD Bio-Techne (#1045241) |
| ISL1 | Chicken | 4-10nM | Molecular Instruments |
| SIX6 | Chicken | 4-10nM | Molecular Instruments |
| POMC | Chicken | 4-10nM | Molecular Instruments |
| **Fluorophore** | | | |
| Opal *480* | 1:500 | Akoya Bio FP1500001KT | |
| Opal *570* | | 1:500 | Akoya Bio FP1488001KT |
| Opal *690* | | 1:500 | Akoya Bio FP1497001KT |
| *HCR v3.0 amplifier B1* | | | Molecular Instruments Alexa Fluor 488 |
| *HCR v3.0 amplifier B3* | | | Molecular Instruments Alexa Fluor 647 |
| *HCR v3.0 amplifier B4* | | | Molecular Instruments Alexa Fluor 546 |

**Supplementary table 4.** List of RNAscope probes and fluorophores, related to STAR methods.

| 10X reaction ID | Approach | Sample | Day | Cells before filtering | Cells after filtering |
|---|---|---|---|---|---|
| D4 | scRNA-seq | BATCH-0 ARC d16, BATCH-1 ARC d16, BATCH-2 ARC d16, BATCH-3 ARC d16 | day 16 | 10939 | 8392 |
| D1 | snRNA-seq | BATCH-0_D25, BATCH-1_D25, BATCH-2_D25, BATCH-3_D25, BATCH-0_D50, BATCH-0_D70, BATCH-0_D50_organoid, BATCH-0_D70_organoid | day 25, 50, 70 | 5458 | 3639 |
| E1 | snRNA-seq | BATCH-0_D25, BATCH-1_D25, BATCH-2_D25, BATCH-3_D25, BATCH-0_D50, BATCH-0_D70, BATCH-0_D50_organoid, BATCH-0_D70_organoid | day 25, 50, 70 | 6036 | 3905 |
| F1 | snRNA-seq | BATCH-0_D25, BATCH-1_D25, BATCH-2_D25, BATCH-3_D25, BATCH-0_D50, BATCH-0_D70, BATCH-0_D50_organoid, BATCH-0_D70_organoid | day 25, 50, 70 | 5534 | 3646 |
| G1 | snRNA-seq | BATCH-1_D50_CNT, BATCH-1_D50_control1, | day 50 | 7523 | 4637 |

| | | | | | |
|---|---|---|---|---|---|
| | | BATCH-1_D50_control2, BATCH-1_D50_control3, BATCH-1_D50_control4, BATCH-1_D50_FGF1, BATCH-1_D50_organoid | | | |
| H1 | snRNA-seq | BATCH-1_D50_CNT, BATCH-1_D50_control1, BATCH-1_D50_control2, BATCH-1_D50_control3, BATCH-1_D50_control4, BATCH-1_D50_FGF1, BATCH-1_D50_organoid | day 50 | 7231 | 4763 |
| C2 | snRNA-seq | BATCH-1_D50_CNT, BATCH-1_D50_control1, BATCH-1_D50_control2, BATCH-1_D50_control3, BATCH-1_D50_control4, BATCH-1_D50_FGF1, BATCH-1_D50_organoid | day 50 | 6838 | 4348 |
| D2 | snRNA-seq | BATCH-2_D50_CNT, BATCH-2_D50_control1, BATCH-2_D50_control2, BATCH-2_D50_control3, BATCH-2_D50_control4, BATCH-2_D50_FGF1, BATCH-2_D50_organoid | day 50 | 5771 | 3232 |
| E2 | snRNA-seq | BATCH-2_D50_CNT, | day 50 | 6620 | 3041 |

| | | | | | |
|---|---|---|---|---|---|
| | | BATCH-2_D50_control1, BATCH-2_D50_control2, BATCH-2_D50_control3, BATCH-2_D50_control4, BATCH-2_D50_FGF1, BATCH-2_D50_organoid | | | |
| F2 | snRNA-seq | BATCH-2_D50_CNT, BATCH-2_D50_control1, BATCH-2_D50_control2, BATCH-2_D50_control3, BATCH-2_D50_control4, BATCH-2_D50_FGF1, BATCH-2_D50_organoid | day 50 | 6432 | 3514 |
| G2 | snRNA-seq | BATCH-3_D50_CNT, BATCH-3_D50_control1, BATCH-3_D50_control2, BATCH-3_D50_control3, BATCH-3_D50_control4, BATCH-3_D50_FGF1, BATCH-3_D50_organoid | day 50 | 5173 | 1691 |
| H2 | snRNA-seq | BATCH-3_D50_CNT, BATCH-3_D50_control1, BATCH-3_D50_control2, BATCH-3_D50_control3, BATCH-3_D50_control4, BATCH-3_D50_FGF1, BATCH-3_D50_organoid | day 50 | 4194 | 1557 |

| | | | | | |
|---|---|---|---|---|---|
| A6 | snRNA-seq | BATCH-1_D70_CNT, BATCH-1_D70_control1, BATCH-1_D70_control2, BATCH-1_D70_control3, BATCH-1_D70_control4, BATCH-1_D70_FGF1, BATCH-1_D70_organoid | day 70 | 4845 | 1043 |
| A7 | snRNA-seq | BATCH-1_D70_CNT, BATCH-1_D70_control1, BATCH-1_D70_control2, BATCH-1_D70_control3, BATCH-1_D70_control4, BATCH-1_D70_FGF1, BATCH-1_D70_organoid | day 70 | 4075 | 1018 |
| A9 | snRNA-seq | BATCH-1_D70_CNT, BATCH-1_D70_control1, BATCH-1_D70_control2, BATCH-1_D70_control3, BATCH-1_D70_control4, BATCH-1_D70_FGF1, BATCH-1_D70_organoid | day 70 | 3910 | 958 |
| A11 | snRNA-seq | BATCH-2_D70_CNT, BATCH-2_D70_control1, BATCH-2_D70_control2, BATCH-2_D70_control3, BATCH-2_D70_FGF1, BATCH-2_D70_organoid | day 70 | 2683 | 1018 |

| | | | | | |
|---|---|---|---|---|---|
| B5 | snRNA-seq | BATCH-2_D70_CNT, BATCH-2_D70_control1, BATCH-2_D70_control2, BATCH-2_D70_control3, BATCH-2_D70_FGF1, BATCH-2_D70_organoid | day 70 | 2449 | 970 |
| B6 | snRNA-seq | BATCH-2_D70_CNT, BATCH-2_D70_control1, BATCH-2_D70_control2, BATCH-2_D70_control3, BATCH-2_D70_FGF1, BATCH-2_D70_organoid | day 70 | 2461 | 1184 |
| B7 | snRNA-seq | BATCH-3_D70_CNT, BATCH-3_D70_control1, BATCH-3_D70_control2, BATCH-3_D70_control3, BATCH-3_D70_FGF1, BATCH-3_D70_organoid | day 70 | 3330 | 231 |
| B8 | snRNA-seq | BATCH-3_D70_CNT, BATCH-3_D70_control1, BATCH-3_D70_control2, BATCH-3_D70_control3, BATCH-3_D70_FGF1 | day 70 | 2277 | 192 |
| B10 | snRNA-seq | BATCH-3_D70_control1, BATCH-3_D70_control2, BATCH-3_D70_control3, BATCH-3_D70_FGF1 | day 70 | 995 | 89 |

**Supplementary table 5.** Overview of scRNAseq and snRNAseq 10x experiments with RC17 as cell line and BMP4 5-14 as BMP timing, related to STAR methods**.**

| Sample | Approach | Bmp timing | Cell line | Day | Cells before filtering | Cells after filtering |
|---|---|---|---|---|---|---|
| AM30E_d16 | snRNA-seq | BMP4 5-7 | RC17 | day 16 | 4885 | 3755 |
| AM30C_d16 | snRNA-seq | BMP4 5-11 | RC17 | day 16 | 4579 | 3499 |
| AM34E_d16 | snRNA-seq | BMP4 5-7 | KOLF | day 16 | 3495 | 2909 |
| AM30D_d16 | snRNA-seq | BMP4 5-9 | RC17 | day 16 | 3504 | 2781 |
| AM34D_d16 | snRNA-seq | BMP4 5-9 | KOLF | day 16 | 3294 | 2713 |
| AM30B_d16 | snRNA-seq | BMP4 5-14 | RC17 | day 16 | 3343 | 2678 |
| AM34C_d16 | snRNA-seq | BMP4 5-11 | KOLF | day 16 | 2936 | 2403 |
| AM34K_d16 | snRNA-seq | BMP4 5-7 | BIOi010 | day 16 | 2864 | 2317 |
| AM34H_d16 | snRNA-seq | BMP4 5-11 | BIOi010 | day 16 | 2817 | 2290 |
| AM34F_d16 | snRNA-seq | no BMP4 | BIOi010 | day 16 | 2814 | 2189 |
| AM34B_d16 | snRNA-seq | BMP4 5-14 | KOLF | day 16 | 2639 | 2167 |
| AM34I_d16 | snRNA-seq | BMP4 5-9 | BIOi010 | day 16 | 2623 | 2138 |
| AM34G_d16 | snRNA-seq | BMP4 5-14 | BIOi010 | day 16 | 2477 | 2015 |
| AM34A_d16 | snRNA-seq | no BMP4 | KOLF | day 16 | 1951 | 1614 |
| AM30A_d16 | snRNA-seq | no BMP4 | RC17 | day 16 | 1021 | 804 |
| AM34D_d60 | snRNA-seq | BMP4 5-9 | KOLF | day 50 | 5053 | 3747 |
| XAM11_1_B_d50 | snRNA-seq | no BMP4 | RC17 | day 50 | 4877 | 3744 |
| AM34K_d50 | snRNA-seq | BMP4 5-7 | BIOi010 | day 50 | 5055 | 3681 |
| AM34B_d50 | snRNA-seq | BMP4 5-14 | KOLF | day 50 | 4529 | 3311 |
| XAM11_2_A_d50 | snRNA-seq | no BMP4 or 7 | KOLF | day 50 | 4406 | 3240 |
| AM34G_d50 | snRNA-seq | BMP4 5-14 | BIOi010 | day 50 | 3762 | 2986 |
| AM34I_d50 | snRNA-seq | BMP4 5-9 | BIOi010 | day 50 | 3978 | 2979 |
| AM34E_d60 | snRNA-seq | BMP4 5-7 | KOLF | day 50 | 3745 | 2912 |
| XAM11_2_B_d50 | snRNA-seq | no BMP4 or 7 | RC17 | day 50 | 3951 | 2910 |
| XAM11_2_C_d50 | snRNA-seq | no BMP4 or 7 | BIOi010 | day 50 | 3863 | 2892 |
| AM30D_d50 | snRNA-seq | BMP4 5-9 | RC17 | day 50 | 3559 | 2689 |
| AM30C_d50 | snRNA-seq | BMP4 5-11 | RC17 | day 50 | 5357 | 2683 |

| | | | | | | |
|---|---|---|---|---|---|---|
| AM34H_d50 | snRNA-seq | BMP4 5-11 | BIOi01<br>0 | day 50 | 3547 | 2660 |
| AM30E_d50 | snRNA-seq | BMP4 5-7 | RC17 | day 50 | 3242 | 2491 |
| AM30B_d80 | snRNA-seq | BMP4 5-14 | RC17 | day 80 | 3049 | 2319 |
| AM39_d110 | snRNA-seq | BMP4 5-14 | RC17 | day 110 | 2742 | 2253 |
| XAM11_1_A_d50 | snRNA-seq | no BMP4 | KOLF | day 50 | 2687 | 2098 |
| AM34C_d50 | snRNA-seq | BMP4 5-11 | KOLF | day 50 | 2572 | 1908 |
| AM30B_d50 | snRNA-seq | BMP4 5-14 | RC17 | day 50 | 2046 | 1174 |
| XAM11_1_C_d50 | snRNA-seq | no BMP4 | BIOi01<br>0 | day 50 | 706 | 529 |

**Supplementary table 6.** Overview of snRNAseq Parse Biosciences experiments, related to STAR methods**.**

## Supplemental references


1. Herb, B.R., Glover, H.J., Bhaduri, A., Colantuoni, C., Bale, T.L., Siletti, K., Hodge, R., Lein, E., Kriegstein, A.R., Doege, C.A., et al. (2023). Single-cell genomics reveals region-specific developmental trajectories underlying neuronal diversity in the human hypothalamus. Sci. Adv. *9*, eadf6251. https://doi.org/10.1126/sciadv.adf6251.
2. Tadross, J.A., Steuernagel, L., Dowsett, G.K.C., Kentistou, K.A., Lundh, S., Porniece, M., Klemm, P., Rainbow, K., Hvid, H., Kania, K., et al. (2025). A comprehensive spatio-cellular map of the human hypothalamus. Nature *639*, 708–716. https://doi.org/10.1038/s41586-024-08504-8.
3. Braun, E., Danan-Gotthold, M., Borm, L.E., Lee, K.W., Vinsland, E., Lönnerberg, P., Hu, L., Li, X., He, X., Andrusivová, Ž., et al. (2023). Comprehensive cell atlas of the first-trimester developing human brain. Science (1979). *382*. https://doi.org/10.1126/science.adf1226.
